# Physician burnout in the Eastern Mediterranean region: influence of gender and related factors – Systematic review and meta-analysis

**DOI:** 10.7189/jogh.11.04043

**Published:** 2021-07-17

**Authors:** Sathyanarayanan Doraiswamy, Karima Chaabna, Anupama Jithesh, Ravinder Mamtani, Sohaila Cheema

**Affiliations:** Institute for Population Health, Weill Cornell Medicine – Qatar

## Abstract

**Background:**

While recent reviews highlight high burnout prevalence among physicians in the World Health Organization’s (WHO) Eastern Mediterranean Region (EMR), there has been a limited exploration into the role of gender and related factors in this problem.

**Methods:**

We conducted a systematic review and meta-analysis of studies on the prevalence of physician burnout and its relationship to gender, physician specialties, and age in the WHO’s EMR based on the Cochrane Handbook for Systematic Reviews. We searched PubMed, Embase, PsycINFO, Google Scholar, and Al Manhal databases and synthesized the findings from the included studies.

**Results:**

Among the 78 studies included, data was available from 16/22 (72.7%) countries and territories in the EMR covering a total of 16 016 physicians. The pooled prevalence of overall burnout among physicians in the region was estimated to be 24.5%. Among the sub-components of burnout, we estimated a high pooled prevalence of 44.26% for emotional exhaustion followed by 37.83% for depersonalization and 36.57% for low personal achievement. There was a statistically significant difference in the prevalence across the countries in the EMR and among the sub-categories of specialist medical practitioners. There was no statistically significant difference across the two genders at a regionally aggregated level.

**Conclusions:**

The levels of physician burnout including the three sub-components in EMR are high by any standards. Based on our review of available studies, it is difficult to ascertain gender differences with certainty in burnout levels among physicians in the EMR nations. There is a need for better quality studies in this area.

Occupational burnout has been evident for several decades in persons working in demanding professions. Though the term burnout was coined in 1974 by Freudenberger [1], it was Maslach who differentiated burnout into the dimensions of emotional exhaustion: an emotionally drained state, depersonalization; dehumanized and cynical attitudes towards clients; and a diminished sense of personal accomplishment, alongside a decline in feelings of competence and ability to success in work [2]. Healthcare workers (HCWs) are identified as a high-risk group for burnout given that high pressure is inherent to health care settings associated with challenges of clinical practice, litigious environments, heavy workload, and non-commensurate income compensation [3,4]. Among HCWs, physicians are more prone to burnout than others, given the added stress associated with administrative decision making, particularly in resource limited contexts [3]. Burnout among physicians impacts not only their personal, physical, mental, and social health, but can also translate to poor clinical decision making and medical errors that may compromise patient safety [5,6].

While substantial evidence exists highlighting burnout prevalence among physicians, there has been limited exploration of the gender dimension [7]. Studies exploring gender have demonstrated inconsistent findings and are suggestive of geographical differences [5] – in North America, for instance, female physicians have a higher burnout prevalence than male physicians [8], whereas the opposite exists in Europe [9]. Two recent reviews on the World Health Organisation (WHO) Eastern Mediterranean Region (EMR) demonstrate a high burnout prevalence among HCWs, [10,11] with one systematic review estimating that 40%-60% of HCWs in the region could be suffering from burnout at a given point in time [11]. Although not a primary outcome in these reviews, the female gender has been identified as a factor associated with burnout [10,11]. However, the reviews combined all categories of HCWs and included physicians only as a subgroup, making it difficult to draw specific conclusions. Age is another factor that has been associated with the prevalence of burnout – some studies report that younger physicians are more prone to burnout, [12] while other studies identify two peaks of high prevalence among the youngest and the oldest cohorts [11,13]. A global review reported that gender differences for burnout among physicians were inconsistent and often disappeared after adjustment for age and other factors [14].

Varying burnout prevalence among the different medicine specialties has been previously established in published literature. However, there is considerable variation among countries on the type of specialties more prone to burnout [15]. It is well known that specialty choice in medicine is guided by gender-related considerations [16] – in the United Kingdom (UK), men are generally more likely to become specialists. Gender, age, and specialties can be independent factors for burnout but can also be inter-related as studies have shown [7,17].

The emergence of burnout among physicians in the EMR as a public health concern and the possible role of gender and related factors has been identified in previous systematic reviews. We aimed to conduct a systematic review synthesizing available evidence on the differences in the burnout prevalence among physicians in the EMR countries associated to gender, age, and physician categories/specialties. We recognize that the devastating impact of the COVID-19 pandemic on physicians is likely to skew the burnout levels among physicians [18]. The data generated from the review can subsequently provide a baseline to better understand the impact of the pandemic on physician burnout in the region.

## METHODS

### Overview

We conducted a systematic review and meta-analysis of primary studies on the prevalence of burnout and the influence of gender, physician categories and specialties, and age in the WHO EMR based on the Cochrane Handbook for Systematic Reviews [19]. The protocol was registered on PROSPERO, [20] with the identification number CRD42020187512. The systematic review and meta-analysis are reported using the Preferred Reporting Items for Systematic Reviews and Meta-Analyses (PRISMA) guidelines [21,22] and the PRISMA for Abstracts Checklist [23]. The PRISMA checklists for the review and the abstracts have been enclosed as Table S1 in the **Online Supplementary Document**. This systematic review and meta-analysis form one part of a research project aiming to synthesize the scholarly literature on population health issues in the EMR [24-33].

### Definitions

Our definitions for burnout and its sub-components (emotional exhaustion [EE], depersonalization [DP], and a diminished sense of (low) personal accomplishment [PA]) were based on the Maslach Burnout Inventory-Human Services Survey instrument (MBI-HSS) [34]. The MBI-HSS defines high EE as score ≥27, high DP as >10, and low PA as <33 [2,34]. Overall burnout is defined inconsistently in studies using varying combinations of high EE, high DP, and low PA (ranging from any of the three to all three) [35,36]. We identified eight possible combinations for the definition of overall burnout (ranging from high scores in all three sub-components to a high score in at least any one sub-component). We grouped the studies based on the definition and factored this in our qualitative and quantitative synthesis. When the same study had used more than one definition for overall burnout, we included the most used definition only (high EE, high DP, and low PA) for the meta-analysis.

We categorized physician specialties using the Organization for Economic Co-operation and Development (OECD) Health Statistics classification. This classification includes three main specialty categories (*generalist medical practitioners, specialist medical practitioners, and medical doctors not further defined*) and eight subcategories (*general practitioners, other generalists, general pediatricians, obstetricians and gynecologists, psychiatrists, medical group of specialists, surgical group of specialists, and other specialists not elsewhere defined*). Two of the sub-categories include medical and surgical group of specialists, which respectively include 20 (*eg, internal medicine, cardiology, endocrinology, etc.*) and 11 specialties (*e.g. general surgery, neurological surgery, plastic surgery, etc.*) [37].

### Search strategy

SD and AJ searched PubMed, Embase, PsycINFO, and Google Scholar search engines for all relevant primary studies published until June 2020 with no time and language restrictions. June 2020 was chosen as the cut-off point as we wanted to restrict our review to studies conducted before the COVID-19 pandemic. Given the time taken to publish articles and the lag associated with indexing, it is highly likely that most studies conducted in 2019 would have been published by June 2020. Al Manhal database was also searched for grey literature from the region. The chosen databases will provide the most comprehensive collection of relevant articles for this review. The term ‘burnout’ was searched using several keywords and MeSH terms in various combinations, such as ‘burnout’ or ‘emotional exhaustion’ or ‘depersonalization’ or ‘personal accomplishment’, along with ‘Arab’ or ‘Middle East’ or ‘Eastern Mediterranean’ or ‘Gulf Cooperation Council’ and similar words to describe the EMR countries. The search terms were modified for all databases and the search strategy for each database is included in Appendix S2 in the **Online Supplementary Document**. We also manually searched reference lists of all included studies for additional primary studies. KC, SC, and RM have extensive experience in publishing Systematic Reviews and Metanalysis. SD and AJ have also published systematic reviews with the other authors. Search strategy was discussed and agreed between all authors and endorsed by a senior Librarian.

### Eligibility criteria

#### Inclusion

The population in this systematic review and meta-analysis included general physicians and those in any medical specialty from the EMR. We included studies conducted in Palestine and the 21 member states in the EMR, namely Afghanistan, Bahrain, Djibouti, Egypt, Iran, Iraq, Jordan, Kuwait, Lebanon, Libya, Morocco, Oman, Pakistan, Qatar, Saudi Arabia, Somalia, Sudan, Syria, Tunisia, United Arab Emirates (UAE), and Yemen [38].

We aimed to enhance comparability by including only studies using the same tool for burnout assessment. Since MBI-HSS is the most common standardized tool used for burnout assessment, this review therefore includes only those primary studies in which MBI-HSS was used to measure burnout prevalence. Studies using cross-sectional, case-control, and cohort study designs were included. We included studies with published abstracts or articles in English, Arabic, and French (articles in other languages were included only if the abstract were in any of the aforementioned three languages).

#### Exclusion

Experimental, interventional, and qualitative studies as well as studies conducted in countries outside the EMR region were excluded. Systematic reviews were excluded; however, all the primary studies included in the identified systematic reviews that met the eligibility criteria were included. We also excluded studies that did not use the MBI-HSS tool or used the less standardized/abbreviated form of MBI-HSS to assess burnout prevalence. Studies without published abstracts in English, Arabic, or French were excluded. Studies reporting burnout prevalence in a group of HCWs without clearly identifying the prevalence among physicians were also excluded.

#### Study selection

The online software Rayyan (Rayyan Systems Inc., Cambridge, Massachusetts, United States of America) [39] was used by AJ to remove all duplicates. Title/abstract screening was conducted by AJ, and SD checked all the excluded articles. Articles were then screened for eligibility by both AJ and SD independently reading the full text. Discrepancies were resolved in consultation with KC. Articles in Arabic and French and those in Persian published with an English abstract were screened by KC. Disagreements at each stage were resolved through discussion between the three reviewers and SC under supervision of RM.

#### Data extraction

Data was extracted from the English articles by AJ and checked by SD. The articles in Arabic, French, and Persian published with an English abstract were extracted by KC. The data extraction database was developed post-piloting in a small study sample. The following information was extracted from each article: the study design, study period, study setting including country, study population-specific medical specialty where available, participant demographics, sample size, and study response/completion rate, burnout prevalence results using MBI-HSS, and limitations. In addition, we extracted factors associated with a high burnout prevalence and also any recommendations provided in the articles.

### Quality assessment

The risk of bias for individual studies were assessed using the tools recommended by the National Heart, Lung, and Blood Institute (NHLBI) [40]. The studies were categorized as good, fair, or poor depending on whether the studies had: (i) a clearly defined study objective/ research question, (ii) a clear description of the study population, (iii) participation rate (>50%), (iv) clearly defined inclusion and exclusion criteria, (v) sample justification or power description or variance and effect, (vi) study population that was recruited from a similar population during the same period.

### Qualitative synthesis

We qualitatively synthesized the findings from all the included primary studies. The characteristics of the included studies are summarized in Table S3 of the **Online Supplementary Document**. The list of the excluded studies at the end of full text screening is provided in Appendix S4 of the **Online Supplementary Document**. The reference list in the main manuscript includes only select studies which provided substantive contribution to the qualitative synthesis.

### Quantitative synthesis

We computed pooled prevalence estimates and their 95% confidence intervals (95% CI) using meta-analysis based on the random-effects model. Using the ‘R’ software, pooling was conducted with the ‘PLOGIT’ transformation method, which uses the logit transformation of the proportion. Studies reporting burnout prevalence were eligible to be included in our meta-analysis if their sample size was equal to or more than 20.

The heterogeneity between studies was assessed using the I^2^ statistic, which describes the percentage of variation across studies that is due to heterogeneity rather than chance. The heterogeneity was considered as insignificant when I^2^<50%. To explore heterogeneity between studies, subgroup meta-analysis was conducted to estimate pool prevalence of overall burnout and the three sub-components (EE, PA, and DP) by country, gender, professional category and subcategory, specialization, study quality assessment level (poor, fair, good, and unclear), overall burnout definition used, type of instrument’s language (MBI-HSS, MBI Arabic, MBI in other languages) and instrument’s cut-off, (standard, non-standard, and unclear).

We conducted a sub-group analysis and calculated pooled prevalence only when there were two or more prevalence rates (referred to as ‘prevalence measures’ in the manuscript) for that sub-group. Differences between subgroups and their ranking were assessed using the Q test and Kruskal-Wallis test respectively. We plotted study size against log odds as this method has been established as a more accurate way of assessing publication bias [41]. Egger’s test was used to determine significance in publication bias among the included studies. Statistical significance was considered at *P*-value ≤0.05.

## RESULTS

### Characteristics of included studies

Full text screening identified 42 primary studies of relevance to our topic. An additional 36 primary studies were identified from gray literature and reference list checking. At the end of the screening process, we included 78 eligible primary studies in our systematic review (Figure S5 of the **Online Supplementary Document**). Among the 78 studies, prevalence of burnout measures that met our eligibility criteria for inclusion in the meta-analysis for overall burnout were found in 48 studies (61.5%) and one or more of its subcomponents in 67 studies (67%).

The studies included in our review were all conducted between 2003 and 2019. These included data from 16/22 (72.7%) countries and territories in the EMR, covering a total of 16 016 physicians. The studies had a sample size range of 31-3350 and the response rates varied from 25%-100%. The mean age range of the physicians included in the studies was 26.7-47.4 years. Nearly all studies (75/78; 96.1%) included both genders, one study included males only, one study included females only, and in one study the physician gender was not reported.

The majority of studies (69/78; 88.5%) included in our review used the English version of the MBI-HSS and nine studies used MBI-HSS in other languages including Arabic (5/10), French (3/10), and Persian (1/10). Eleven of the 78 studies (14.1%) used cut-off definitions for the burnout subcomponents that were based on the standardized definitions recommended by MBI-HSS. Of the remaining studies, 40/78 (51.3%) used different cut-offs for one or more of the sub-components and mentioned them explicitly, whereas in 27/78 (34.6%) studies the cut offs used were not mentioned or unclear.

### Quality of included studies

Quality assessment of the studies using the adapted NHLBI quality criteria for observation studies found that 14/78 (17.9%) studies were of good quality, 15/78 (19.2%) were of fair quality, and 44/78 (56.4%) were rated as poor quality. On the specific criterion used for quality assessment, we found that 78/78 (100%) of the studies stated a clear research question and had a well-defined study population. While 62/78 (79.5%) of the studies had a response rate greater than 50%, only 27/78 (34.6%) clearly mentioned the inclusion and exclusion criterion for the sample. In most of the studies (76/78; 97.4%), participants were recruited from the same setting (same geography or same workplace). Only 14/78 (17.9%) studies provided a justification for the sample size, power description, or variance and effect size estimates. The quality assessment of the individual studies is included in Table S6 of the **Online Supplementary Document**.

### Prevalence of overall and sub-components of burnout

#### Overall burnout

There were 54 prevalence measures of overall burnout included in our meta-analysis covering 11 381 physicians from 48 studies conducted in EMR. Among the included studies, there was wide variation in the definition of overall burnout. The most commonly used (25/54; 46%) prevalence measure was the conservative burnout definition of high scores in EE and DP and low score in PA. Estimated pooled overall burnout prevalence in the EMR using this definition was 24.5% (95% CI: 15.24-36.94). The more inclusive definition of abnormal scores in ‘any of the three sub-components’ prevalence measures was the second most commonly used in the included studies reporting prevalence measures in the EMR (6/54; 11%), reporting an estimated pooled overall burnout prevalence of 35.6% (95% CI: 12.25-68.55). Four of 54 (7%) studies used the definition ‘a combination of any of the two sub-components’ and reported pooled prevalence measures of 41.6% (95% CI: 18.84-68.71). Considering all the eight definitions used in the included studies, pooled prevalence was significantly different between the various overall burnout definitions utilized (*P* < 0.0001). Estimated pooled prevalence computed from studies using the standard instrument cut-off was significantly higher than pooled prevalence computed from studies using non-standard or unclear cut-off (59.5% vs 25.5% and 46.9%, *P* < 0.05). There were no significant differences in the prevalence based on the language of the MBI-HSS tool and the quality of studies. The relevant statistics are shown in **Table 1**.

**Table 1:** Overall physician burnout prevalence in EMR by definition, instruments, instrument's cut off, and quality assessment

	Years of data collection	Number of prevalence measures	Total sample size		Prevalence range (%)		Effect size		Subgroup comparison	Heterogeneity between studies
							Weighted average prevalence (%) *	**95% CI**	**Q between subgroup tests *P*-value**	***I^2^* (%)**
**Overall burnout definition:**										
High EE and High DP and Low PA		2006-2019	25	7359		0-89	24.51	15.24-36.94	<0.0001	98.60
Any of the 3		2010-2019	6	946		5-89	35.56	12.25-68.55		98.30
High EE + Low PA		2013	1	239		23	22.59	17.73-28.33		N/A
High EE + High DP		2013-2014	1	181		81	81.22	74.86-86.26		N/A
High DP or Low PA		-	-	-		-	-	-		-
High EE + (High DP or Low PA)		NR	2	171		47-58	52.05	44.57-59.43		0.00
Low PA		2018	1	31		35	35.48	20.86-53.44		N/A
High EE		2014	2	641		35-60	48.90	32.22-65.83		85.20
High DP			0	-		-	-	-		-
2 of the 3		2012-2017	4	416		16-76	41.65	18.84-68.71		95.80
Unclear		2009-2014	12	1397		4-92	54.62	35.05-72.86		97.10
**Instrument’s language:**										
MBI Arabic		2006-2017	3	1286		12-78	46.79	15.48-80.84	>0.05	99.10
MBI other languages		2011-2014	2	533		27-32	29.37	26.10-33.85		0
MBI-HSS (English)		2010-2019	49	9562		0-92	35.66	26.43- 46.10		98.40
**Instrument's cut off:**										
Not standard		2006-2019	29	7822		0-89	25.46	17.28-35.84	<0.05	98.30
Standard		2010-2017	7	824		0-92	59.46	27.50-85.01		97.60
Unclear		2010-2019	18	2735		4-85	46.92	32.97-58.98		97.60
**Quality assessment:**										
Fair		2012-2018	12	4373		10-89	38.96	21.92-59.20	>0.05	98.30
Good		2010-2020	11	984		0-70	26.45	14.22-43.83		98.60
Poor		2006-2020	28	3350		3-92	40.22	28.15-53.60		97.80
Unclear		Unclear	3	513		4-71	27.80	5.62-71.36		98.10

EE – emotional exhaustion; DP – depersonalization; PA – personal accomplishment; EMR – Eastern Mediterranean Region, CI – confidence interval, NR – not reported

*Calculated only when two or more data points available.

At study level, we observed a prevalence range of 0% in a sample of 51 physicians in Lebanon [42] to 91.6% among 299 physicians in the UAE [43]. Estimated pooled prevalence of overall burnout, which was computed with at least two prevalence measures, ranged from 10.2% in Oman to 91.6% in the UAE (**Table 2**). Statistically significant differences in the prevalence of overall burnout were identified between the 15 EMR countries (15/16 countries had data that were eligible to be included in the meta-analysis) that reported at least one prevalence measure of overall burnout.

**Table 2:** Overall physician burnout prevalence in EMR by country, profession category, subcategory and specialty

	Years of data collection	Number of prevalence measures	Total sample size	Prevalence range (%)	Effect size		Subgroup comparison	Heterogeneity between studies
					Weighted average prevalence (%)*	**95% CI**	**Q between subgroup tests *P*-value**	***I^2^* (%)**
All countries	2006-2019	54	11 381	0-92	35.8	27.73-44.77	N/A	98.40
**Countries:**								
Egypt	2012-2018	8	961	22-77	60.59	47.31-72.47	<0.0001	92.60
Iran	2018	2	170	71-89	81.14	64.46-91.07		73.50
Iraq	2014-2017	4	1006	4-60	25.52	8.93-54.49		97.30
Kuwait	2010-2011	1	200	21	20.5	15.46-26.66		N/A
Lebanon	NR	1	51	0	0	0.0-100		N/A
Morocco	2018	3	445	32-85	63.39	35.07-84.73		96.50
Oman	2017-2019	4	533	5-16	10.25	5.91-17.20		59.30
Pakistan	2013-2017	6	768	12-58	24.45	14.24-38.68		91.90
Palestine	2013	1	142	10	9.86	5.93-15.96		N/A
Qatar	NR	2	209	16-35	23.87	13.04-39.60		78.90
Saudi Arabia	2010-2019	15	2090	3-89	37.98	22.86-55.85		97.70
Syria	NR	1	3350	19	19.28	17.98-20.65		N/A
Tunisia	2009-2011	3	604	17-34	26.14	19.37-34.28		73.70
United Arab Emirates	2016	2	302	89-92	91.64	87.92-94.29		0.00
Yemen	2006-2007	1	563	12	11.72	9.32-14.65		N/A
**Profession category:**								
General medical practitioners	2010-2019	21	5932	3-89	30.01	18.18-45.28	>0.05	98.60
Specialized medical practitioners	2010-2019	44	2491	0-95	38.69	29.25-49.07		95.3
**Profession cub-category:**								
General practitioner	2010-2019	20	5932	3-89	30.08	17.72-46.23	<0.01	98.5
General pediatricians	2010-2019	6	376	13-87	47.09	22.03-73.71		95.0
Medical interns	2011	1	342	342	28.65	24.11-33.68		N/A
Medical specialists	2010-2019	11	658	0-85	37.06	16.17-64.26		97.00
Obstetrics and Gynecology	2010-2019	5	220	22-86	46.60	20.56-74.64		92.4
Other specialists not classified elsewhere	2016-2017	1	44	7	6.82	2.22-19.11		N/A
Psychiatrists	2017	1	29	29	3.45	0.48-20.79		N/A
Surgical specialists	2010-2019	20	1164	10-95	40.09	28.26-53.2		93.1
**Profession specialty:**								
Anesthesiology	2016-2019	3	77	14-36	24.68	16.33-35.48	>0.05	0
Emergency medicine	2010-2017	6	376	10-80	36.57	18.32-59.71		92.1
General surgery	2010-2017	3	126	14-95	67.9	16.85-95.67		93.2
Intensive care physicians	2017	2	212	35-78	58.29	27.80-83.53		93.9
Internal medicine	2010-2019	4	241	25-85	64.18	38.66-83.58		91.9
Not reported	2010-2019	13	669	3-87	37.00	19.30-59.05		95.2
Oncology	2016-2018	3	206	0-85	18.11	00.30-94.12		98.8
Ophthalmology	2018	1	117	41	41.03	32.49-50.14		N/A
Orthopedics	2018-2019	2	149	19-57	38.7	15.58-68.36		78.2
Oto-rhino-laryngology	2010-2019	3	191	5-45	25.87	09.78-52.92		89.2
Plastic surgery	2015-2018	3	107	18-47	32.99	20.43-48.57		58.8
Radiology	2018-2019	1	20	15	15	04.92-37.58		N/A

EMR – Eastern Mediterranean Region, CI – confidence interval, NR – not reported

*Calculated only when two or more data points available.

**Figure 1:**
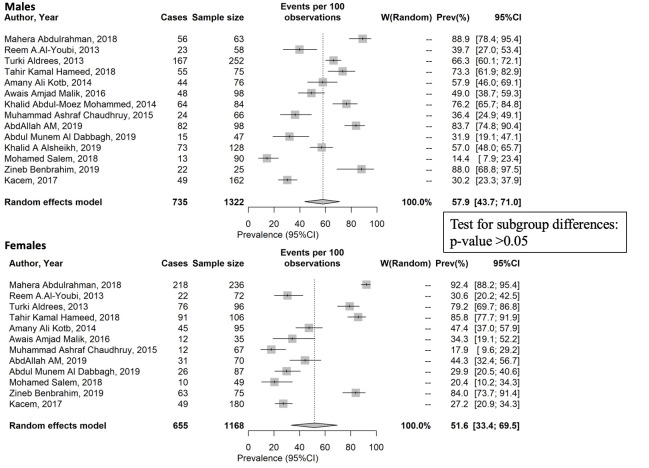
Gender differences in the prevalence of overall physician burnout.

**Figure 2:**
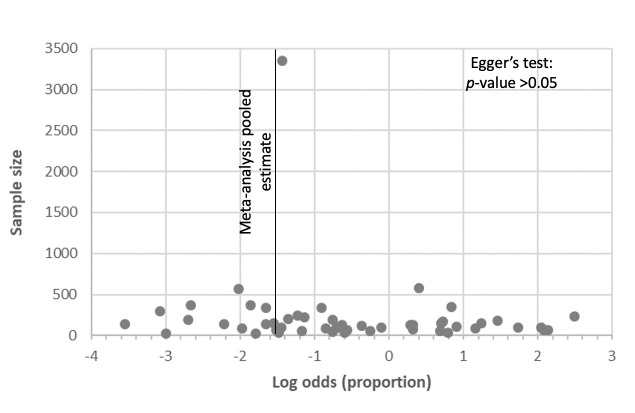
Publication bias assessment in all included studies.

**Figure 3:**
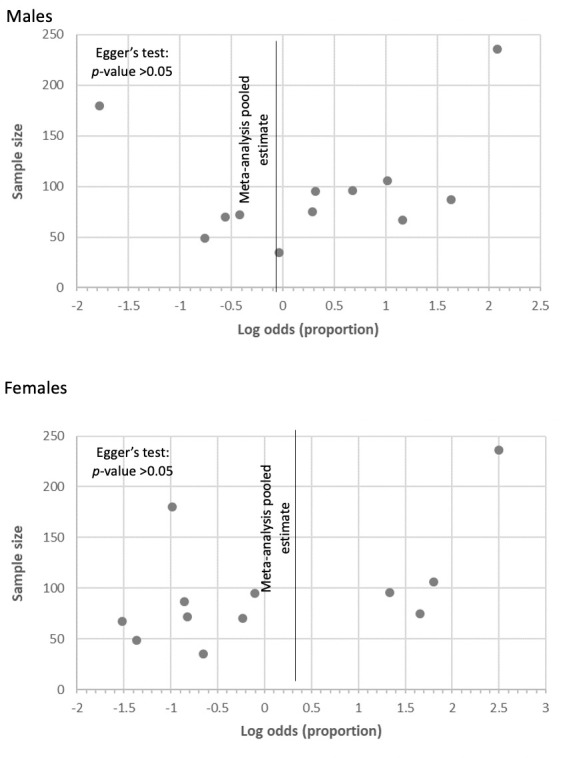
Publication bias assessment in included studies stratified by gender.

Data on overall burnout stratified by gender was reported in eight countries: Egypt, Iraq, Morocco, Pakistan, Qatar, Saudi Arabia, Tunisia, and the UAE. At a regional level, the difference in the estimated pooled prevalence between males (57.9%) and females (51.6%) was not statistically significant (**Figure 1**). At a country level, when gender subgroup analysis was feasible to consider the significant heterogeneity between countries, differences between males and females was detected. Estimated prevalence of overall burnout was significantly higher in males than in females in Egypt (73.8% vs 46.0%, *P* < 0.001) and Pakistan (43.6% vs 24.2%, *P* < 0.05). In Saudi Arabia, no statistically significant difference between males and females was detected. The country level differences are shown in **Table 3**. Egger’s test for publication bias in studies at country level and across gender did not reveal significant publication bias (Figures 2 and 3 respectively). The forest plot showing pooled prevalence across countries is provided in Figure S7 of the **Online Supplementary Document**. Detailed gender-wise prevalence for each country is provided in Table S8 of the **Online Supplementary Document**.

**Table 3:** Overall physician burnout prevalence in each EMR country by gender

	Years of data collection	Number of prevalence measures	Total sample size	Prevalence range (%)	Effect size		Subgroup comparison	Heterogeneity between studies
					Weighted average prevalence (%)*	**95% CI**	**Q between subgroup tests *P*-value**	***I^2^* (%)**
**Egypt:**								
Male	2012-2017	3	258	58-84	73.84	60.14-84.08	<0.001	79.10
Female	2012-2017	2	165	44-47	46.06	38.60-53.70		0
**Iraq:**								
Male	2017	1	47	32	31.91	19.09-47.12	N/A	N/A
Female	2017	1	87	30	29.89	20.54-40.65		N/A
**Morocco:**								
Male	2018	1	25	88	88	68.78-97.45	N/A	N/A
Female	2018	1	75	84	84	73.72-91.45		N/A
**Pakistan**								
Male	2014	2	164	36-49	43.64	35.10-52.57	<0.05	19.40
Female	2014	2	102	18-34	24.18	14.80-36.92		39.6
**Qatar:**								
Male	Not Reported	1	90	14	14.44	7.92-23.43	N/A	N/A
Female	Not Reported	1	49	20	20.41	10.24-34.34		N/A
**Saudi Arabia:**								
Male	2010-2018	4	513	40-73	59.98	47.92-70.95	>0.05	83.90
Female	2010-2018	3	274	31-86	68.57	37.03-89.0		94.9
**Tunisia:**								
Male	Not Reported	1	162	30	30.25	23.29-37.95	N/A	N/A
Female	Not Reported	1	180	27	27.22	20.87-34.34		N/A
**United Arab Emirates:**								
Male	2016	1	63	89	88.89	78.44-95.41	N/A	N/A
Female	2016	1	236	92	92.37	88.21-95.42		N/A

EMR – Eastern Mediterranean Region, N/A – not applicable, CI – confidence interval

*Calculated only when two or more data points available.

While no differences were detected between the main categories of generalist and specialist medical practitioners, we identified significant differences in the prevalence of overall burnout between the different professional subcategories (*P* < 0.05). The highest pooled prevalence of over 40% was observed among general pediatricians (47.09%), obstetrician and gynecologists (46.6%), and surgical specialists (40.1%). The lowest pooled prevalence was observed among the general practitioners (30.08%). No statistically significant differences were detected between the specialties. Kruskal-Wallis rank test did not reveal any statistically significant difference across any gradient of sub-categories and specialties.

We computed the pooled prevalence of overall burnout among physician cohorts with mean age lower and higher than 35 years old. Though the relatively older physician cohort had a lower prevalence of overall burnout (14.91%) when compared to the younger cohort (33.69%), we did not detect any statistical significance.

#### Sub-components of burnout

The definitions of the sub-components of burnout (EE, DP, and PA) were from MBI-HSS in all included primary studies. The pooled prevalence of these subcomponents in the EMR varied: EE was 44.26% (70 prevalence measures, sample size of 14 294 physicians); DP was 37.83% (70 prevalence measures, sample size of 14 277 physicians), and PA was 36.57% (70 prevalence measures, sample size of 14 291 physicians). In each of the three sub-components, the differences between the estimated pooled prevalence observed according to the instrument’s cutoff used by the primary studies were not statistically significant. The detailed meta-analysis outputs of the three sub-components are provided in Appendix S9 of **the** **Online Supplementary Document**. The prevalence of EE, DP, and PA in the EMR, as well as gender and profession category are provided in **Table 4**.

**Table 4:** Prevalence of physician burnout subcomponents: EE, DP and PA in EMR by country, gender, and profession category

	Years of data collection	Number of prevalence measures	Total sample size	Prevalence range (%)	Effect size				Subgroup comparison	Heterogeneity between studies
					Weighted average prevalence (%)*	**95% CI**			**Q between subgroup tests *P* value**	**I^2^ (%)**
**EE**										
**EMR**										
All countries	2003-2019	70	14 294	0-84	44.26	37.99-50.72			<0.0001	97.90
**Gender**										
Male	2008-2017	14	3364	4-83	46.66	32.32-61.57			>0.05	97.7
Female	2008-2017	13	2414	6-78	48.26	35.12-61.66				96.00
**Profession category**										
General medical practitioner	2009-2018	25	6800	0-86	41.79	27.54-57.56			>0.05	99.10
Specialized medical practitioner	2003-2019	55	7328	10-89	49.78	43.58-55.99				95.5
**DP**										
**EMR:**										
All countries	2003-2019	70	14 277	3-95	37.83	31.83-44.22			<0.0001	97.90
**Gender**										
Male	2008-2017	13	3237	15-78	40.34	28.14-53.88			>0.05	97.00
Female	2008-2017	12	2176	11-78	34.48	22.03-49.50				95.6
**Profession category**										
General medical practitioner	2009-2018	32	13 479	3-95	41.78	31.85-52.44			>0.05	99.20
Specialized medical practitioner	2003-2019	66	9206	5-87	42.82	37.82-47.96				95.3
**PA**										
**EMR:**										
All countries	2003-2019	70	14 291	10-99	36.57		31.50-41.95	<0.0001		96.9
**Gender:**										
Male	2008-2017	13	3237	15-54	32.85		25.55-41.09	>0.05		91.9
Female	2008-2017	12	2176	11-56	32.08		22.97-42.79			92.5
**Profession category:**										
General medical practitioner	2009-2018	31	13 418	10-74	28.29		22.79-34.52	>0.05		97.2
Specialized medical practitioner	2003-2019	61	9080	3-86	29.94		24.64-35.84			96.6

EE – emotional exhaustion; DP – depersonalization; PA – personal accomplishment; EMR – Eastern Mediterranean Region

*Calculated only when two or more data points available.

#### Emotional exhaustion

Statistically significant differences in the prevalence of the EE sub-component (*P* < 0.0001) were identified between the 17 EMR countries reporting at least one prevalence measure of EE (Output EE in Appendix S9 of the **Online Supplementary Document**). Pooled prevalence measures of EE ranged from 7.78% in Iraq to 64.97% in Morocco.

Data on EE stratified by gender was reported in seven countries: Bahrain, Egypt, Iran, Lebanon, Pakistan, Saudi Arabia, and Syria. The difference in pooled prevalence between males (46.66%) and females (48.26%) was not statistically significant. Gender subgroup analysis considering the variability between countries, when feasible, demonstrated that prevalence of EE among males and females respectively in Egypt (68.88% vs 65.88%), Iran (18.35% vs 3.7%), Pakistan (33.15% vs 39.12%), Saudi Arabia (39.79% vs 38.27%), and Lebanon (47.39% vs 61.18%) were not significantly different.

Like the overall burnout prevalence, no difference was detected between the generalist and specialist medical practitioners (41.79% and 49.78%). However, we found significant differences in the prevalence of EE between the professional subcategories (*P* < 0.001). The highest pooled prevalence was 61.57% among the general pediatricians. The lowest pooled prevalence (40.76%) was observed among the general practitioners. Notably, statistically significant differences were detected between the specialties (*P* < 0.0001). Pooled prevalence ranged between 39.46% in anesthesiologists and 59.26% in the emergency medicine physicians.

#### Depersonalization

Statistically significant differences in the prevalence of DP (*P* < 0.0001) were identified between the 17 EMR countries reporting at least one prevalence measure of DP (Output DP in Appendix S9 of the **Online Supplementary Document**). Pooled prevalence of DP, which was computed with at least three prevalence measures, ranged from 20.81% in Iran to 57.82% in Egypt.

Data on DP by gender was reported in seven countries: Bahrain, Egypt, Iran, Lebanon, Pakistan, Saudi Arabia, and Syria. The difference in pooled prevalence between males (40.34%) and females (34.48%) was not statistically significant. Gender subgroup analysis considering variability between countries, when feasible, demonstrated that prevalence of DP was not statistically different between males and females respectively in Egypt (61.04% vs 58.63%), Iran (16.67% vs 13.18), Pakistan (40.07% vs 19.34%), and Saudi Arabia (26.52% vs 21.78%).

Like the overall burnout and the EE domains, no differences were detected between the general and specialized medical practitioners (41.78% and 42.82%). However, we identified significant differences in the prevalence of DP between the profession subcategories (*P* < 0.05). The highest pooled prevalence was at 44.15% for the general pediatricians. The lowest pooled prevalence (36.41%) was observed among the obstetricians and gynecologists. Notably, statistically significant differences were detected between the specialties (*P* < 0.0001). Pooled prevalence ranged between 32.26% in emergency medicine physicians and 50.96% in the anesthesiologists.

#### Personal accomplishment

Statistically significant differences in the prevalence of PA (*P* < 0.0001) were identified between the 17 EMR countries reporting at least one prevalence measure of PA (Output PA in Appendix S9 of the **Online Supplementary Document**). Pooled prevalence of low PA, which was computed with at least three prevalence measures, ranged from 23.42% in Morocco to 46.14% in Egypt.

Similar, to the overall burnout and the EE and DP domains, no differences were detected between the generalist and specialist medical practitioners (28.29% and 29.94%). However, we identified significant differences in the prevalence of the PA domain between the profession subcategories (*P* < 0.0001). The highest pooled prevalence was at 39.84% among the surgical specialists. The lowest pooled prevalence was observed among the medical specialists and the general pediatricians (18.85% and 19.14%). Statistically significant differences were detected between the specialties (*P* < 0.0001). Pooled prevalence ranged between 14.72% in internal medicine specialists and 46.21% in the anesthesiologists.

Data on the PA domain by gender was reported in seven countries: Bahrain, Egypt, Iran, Lebanon, Pakistan, Saudi Arabia, and Syria. The difference in pooled prevalence between males (32.85%) and females (32.08%) was not statistically significant. Gender subgroup analysis conducted for each country, when feasible, demonstrated that prevalence of burnout’s PA domain was not statistically different between males and females respectively in Egypt (41.35% vs 41.05%), Iran (38.89% vs 35.41%), Lebanon (22.36% vs 24.92%), Pakistan (48.35% vs 51.35%), and Saudi Arabia (20.97% vs 26.02%).

## DISCUSSION

The pooled prevalence of physicians in the EMR revealed that one in four physicians showed signs of all three burnout sub-components (EE, DP, and PA). Over one third of the physicians showed signs of at least one of the burnout sub-components. We observed a pattern in some of the key findings: 1) there was a statistically significant difference in the prevalence of overall burnout and the three sub-components across the countries in the EMR; 2) there was no statistically significant difference in the pooled prevalence of overall burnout and the three sub-components across males and females at a regionally aggregated level; 3) there was no statistically significant difference in prevalence between the main generalist and specialist medical categories across overall burnout and the three sub-components; 4) there was a statistically significant difference in the prevalence of overall burnout, EE, DP, and low PA among the sub-categories of specialist medical practitioners. In the gender sub-group analysis in each of the countries in the EMR, there was a statistically significant difference in the prevalence of overall burnout in Egypt and Pakistan with male physicians having a higher prevalence than their female counterparts. We also found statistically significant differences in the prevalence of EE, DP, and low PA among the different medical and surgical specialties, but there was variation in the specific specialties that had the highest prevalence across the sub-components.

A recently published global meta-analysis [35] observed substantial variability in estimating burnout prevalence among physicians. This is likely explained by marked variation in assessment methods, burnout definition, and study quality, which prevented the interpretation of the global estimate of pooled burnout prevalence [35]. We identified similar methodological issues when conducting our meta-analysis for the EMR. We were able to minimize variability due to the assessment methods and overall burnout definition by restricting our analysis to the most used tool in assessment (MBI-HSS) and by factoring in all definitions possible for overall burnout in our sub-group analysis. In our meta-analysis, study quality did not appear to significantly contribute to the variability between studies. Therefore, using the most common definition for overall burnout, which is also the most conservative one (abnormal scores in all three sub-components – also referred to as severe burnout in the literature [44]), we estimated a pooled overall burnout prevalence of 24.5% among physicians in the EMR. This prevalence is much higher when compared to the pooled prevalence of 5% among French [44] and 7.5% among German physicians [45]. When comparing overall burnout prevalence based on the more inclusive definition of abnormal scores in any of the three sub-components, the prevalence of 35.6% computed by our meta-analysis was lesser than the prevalence among the physicians in France (49%) [44] and the US (over 50%) [46]. Hence, while burnout in the EMR appears to be less frequent than in other regions, severe burnout appears to be more frequent. This anomaly of higher prevalence of severe burnout but a lower prevalence of abnormal scores in any of the three sub-components in the region needs further investigation. One possible explanation is the development and implementation of mechanisms for early identification of burnout and the provision of coping support in other regions, which may be currently lacking in the EMR [11].

The pooled prevalence of 44.26% for emotional exhaustion (EE), 37.83% for depersonalization (DP), and 36.57% for low personal accomplishment (PA) found in our review was somewhat similar to levels among residents from the US reported in a global systematic review (EE – 38.9%; DP – 43.6%; PA – 34.3%) [47]. These estimates, however, were higher than the levels reported in physicians in France (EE – 21%; DP – 29%; PA – 29%) [44] and also higher than the prevalence among German physicians (EE – 34.1%; DP – 29%; PA – 21.5%) [45]. While there is no benchmark to assess acceptable levels of burnout globally, with over one third of the physicians in the EMR showing signs of burnout and one quarter showing signs of severe burnout, this is a matter of serious concern.

Chemali et.al (2019) describe the Middle East region as a ‘complex health care environment.’ In their systematic review of burnout among health care workers in the region, the authors attribute the high prevalence of burnout (40%-60% in their review) to a) harsh work conditions b) demanding work schedules/stress, and c) exposure to violence and conflict. In discussing additional reasons for health care worker burnout, the authors contrast the Middle East countries to other high-income countries and highlight the lack of attention to burnout. Among other stressors currently facing the health care community, burnout is attributed to “the ever-increasing burden of caring for major public health threats, amidst ongoing regional conflicts and refugee crises with a paucity of resources and shortage of support” [11].

Our review did not find a statistically significant difference in the prevalence of overall burnout, EE, DP, and PA between male and female physicians. A meta-analysis conducted for Latin America also found no gender differences pertaining to burnout [48]. In our sub-group analysis across countries in EMR, we found a higher prevalence among male physicians when compared to females in Pakistan and Egypt. A male preponderance for burnout has been found in European family doctors [9]. None of the studies included in our review were designed to identify gender differences in burnout but included gender as one of the variables while exploring factors associated with burnout. This makes the interpretation of the findings difficult. Though significant gender differences were not consistently identified in our meta-analysis, we cannot conclude that there is an absence of gender differences because there is a lack of well-designed studies in the region exploring this. Well-designed studies providing gender stratified data should be conducted in each EMR country across different specialties, as the predisposition to burnout and its sub-components varies across different specialties. Understanding gender differences is critical, as issues such as gender discrimination, lower salary, and sexual harassment in the workplace could affect female physicians more than male physicians.[7,49] On the other hand, male physicians in some settings have been shown to experience more physical violence than their female counterparts [50].

We did not find any statistically significant difference in the pooled prevalence of the overall burnout across the two age groups we stratified (<35 years, 33.69%; >35 years, 14.91%). Very few studies included physicians aged over 45 years. This could have been due to the unwillingness of senior physicians to participate in burnout-related studies. Given the lack of studies with an adequate inclusion of the higher age group, a prevalence of 33.69% among the <35 age group is similar to recent studies that have debunked the myth that burnout is a late career manifestation [51]. Studies have shown that younger physicians may have higher stress compared to their older colleagues and the residency period itself could herald the onset of burnout [52].

Though our review found a higher prevalence of burnout and its sub-components among the specialist medical practitioners than generalist medical practitioners, we did not identify a statistically significant difference. However, the difference in the prevalence among the sub-categories of specialist medical practitioners and the various specialties under it, signifies an important finding on the predisposition of certain specialties for burnout and its sub-components. Interestingly in our study, we found the general pediatricians to have a higher prevalence of overall burnout, higher EE, higher DP, but not low PA. Where we were able to compute a pooled prevalence, we observed that emergency medicine physicians had very high prevalence of EE, while anesthesiologists had very high prevalence of DP and low PA. The differences we see in the higher predisposition of various specialties to the different components of burnout signify a need to analyze burnout and critically look at each sub-component among the different specialty groups [3]. The higher preponderance of specialist physicians to burnout in general and some specialties over others has been documented in other reviews [53,54]. Given that there was no pattern in the prevalence of burnout and its sub-components among specific specialties, a gender pattern based on a gender’s dominance in that specialty cannot be made. This lack of pattern could also have been due to the lack of adequate studies to find pooled prevalence among the different specialties. We see an important role for professional societies/ physician groups of the different medical specialties in conducting further research needed to address known gaps in their respective specialties. The positive effect of group memberships and the active role that professional societies in addressing physician burnout has been demonstrated in other studies [55,56].

This review reinforces a substantial prevalence of burnout and its sub-components among physicians in the EMR and is a wakeup call for action. A recent systematic review comparing different interventions to address burnout among physicians found greater benefit from organizational interventions (improving teamwork, enhancing job control, and increasing levels of decision making etc.) than physician-directed interventions (mindfulness, cognitive behavioural techniques, improving personal communication, etc.) [57]. Given the increasing risk of burnout among physicians during the COVID-19 pandemic, [58] the implications of this study highlighting the pre-existing magnitude of burnout is a call for urgent action to halt its further increase during the pandemic and beyond.

Our review has strengths in having been designed in line with the Cochrane Handbook for Systematic Reviews, including registration of the ‘a priori’ protocol and addressing variability between studies identified in previously published meta-analysis, [35] but it is also likely affected by the substantial level of heterogeneity found between the studies. As observed in the meta-analysis of observational studies, this heterogeneity could be attributed to several study-related factors, as well as to different health systems, types of training, and salary structures in the region. Though we did our best to extract data from the English abstracts of the studies published in Persian, we could have missed some important information from these studies and any others that did not have an English abstract. The decision to include only studies that used MBI-HSS helped homogenization and comparability but the differing cut-off points and the lack of consensus regarding the definition of overall burnout in these studies compromised the level of comparison possible [59]. Though other tools are less standardized and popular than MBI-HSS, the fact that we did not include studies which used tools other than MBI-HSS for measuring burnout could also be seen as limiting comprehensiveness.

## CONCLUSION

Physician burnout can have serious consequences at individual, health system, societal, and national levels. By any standards, the levels of physician burnout in the EMR are high, and this includes three sub-components of burnout – emotional exhaustion, depersonalization, and low personal accomplishment. Poor quality studies fail to provide certainty when exploring gender-related differences in burnout levels among physicians within the region. Given the variation in health care systems among EMR countries, each country should address physician burnout at national and sub-national levels by better understanding of the drivers of burnout and its sub-components in each setting and implementing evidence-based prevention and remedial measures. Professional societies of physicians and specialists, physician support groups and licensing boards can play an important role in helping better understand the burnout problem in various specialties and provide appropriate guidance to the physicians. They can collaborate to support and guide the development and implementation of evidence-based, specialty-specific physician friendly policies and programmes. The COVID-19 pandemic is a stark reminder of the protective role played by physicians, and it is surely reasonable to demand that the protectors of population health are themselves safeguarded from burnout while doing that.

## Additional material

Online Supplementary Document
