# Supplementary material for: Physician burnout in the Eastern Mediterranean region: influence of gender and related factors – Systematic review and meta-analysis
Source: J Glob Health. 2021 Jul 17;11:04043. doi: 10.7189/jogh.11.04043 (PMC8285757; doi:10.7189/jogh.11.04043)
Supplement: Online Supplementary Document [file jogh-11-04043-s001.pdf]

Table S1: PRISMA 2009 Checklist

Section/topic	#	Checklist item	Reported on page #
		TITLE	
Title	1	Identify the report as a systematic review, meta-analysis, or both.	1
		ABSTRACT	
Structured summary	2	Provide a structured summary including, as applicable: background; objectives; data sources; study eligibility criteria, participants, and interventions; study appraisal and synthesis methods; results; limitations; conclusions and implications of key findings; systematic review registration number.	1
		INTRODUCTION	
Rationale	3	Describe the rationale for the review in the context of what is already known.	2-3
Objectives	4	Provide an explicit statement of questions being addressed with reference to participants, interventions, comparisons, outcomes, and study design (PICOS).	3-5
		METHODS	
Protocol and registration	5	Indicate if a review protocol exists, if and where it can be accessed (e.g., Web address), and, if available, provide registration information including registration number.	3
Eligibility criteria	6	Specify study characteristics (e.g., PICOS, length of follow-up) and report characteristics (e.g., years considered, language, publication status) used as criteria for eligibility, giving rationale.	4-5
Information sources	7	Describe all information sources (e.g., databases with dates of coverage, contact with study authors to identify additional studies) in the search and date last searched.	4-5
Search	8	Present full electronic search strategy for at least one database, including any limits used, such that it could be repeated.	Supplementary material (SM) 2
Study selection	9	State the process for selecting studies (i.e., screening, eligibility, included in systematic review, and, if applicable, included in the meta-analysis).	5-6
Data collection process	10	Describe method of data extraction from reports (e.g., piloted forms, independently, in duplicate) and any processes for obtaining and confirming data from investigators.	6
Data items	11	List and define all variables for which data were sought (e.g., PICOS, funding sources) and any assumptions and simplifications made.	6
Risk of bias in individual studies	12	Describe methods used for assessing risk of bias of individual studies (including specification of whether this was done at the study or outcome level), and how this information is to be used in any data synthesis.	6
Summary measures	13	State the principal summary measures (e.g., risk ratio, difference in means).	6-7
Synthesis of results	14	Describe the methods of handling data and combining results of studies, if done, including measures of consistency (e.g., I^2) for each meta-analysis.	6-7
Risk of bias across studies	15	Specify any assessment of risk of bias that may affect the cumulative evidence (e.g., publication bias, selective reporting within studies).	6

Additional analyses	16	Describe methods of additional analyses (e.g., sensitivity or subgroup analyses, meta-regression), if done, indicating which were pre-specified.	6-7
RESULTS			
Study selection	17	Give numbers of studies screened, assessed for eligibility, and included in the review, with reasons for exclusions at each stage, ideally with a flow diagram.	Figure 1/ SM 4
Study characteristics	18	For each study, present characteristics for which data were extracted (e.g., study size, PICOS, follow-up period) and provide the citations.	Tables 1-3; Figure 2 and SM 3,6&7
Risk of bias within studies	19	Present data on risk of bias of each study and, if available, any outcome level assessment (see item 12).	SM 5
Results of individual studies	20	For all outcomes considered (benefits or harms), present, for each study: (a) simple summary data for each intervention group (b) effect estimates and confidence intervals, ideally with a forest plot.	Tables 1-3
Synthesis of results	21	Present results of each meta-analysis done, including confidence intervals and measures of consistency.	Not applicable
Risk of bias across studies	22	Present results of any assessment of risk of bias across studies (see Item 15).	SM 5
Additional analysis	23	Give results of additional analyses, if done (e.g., sensitivity or subgroup analyses, meta-regression [see Item 16]).	SM 7
DISCUSSION			
Summary of evidence	24	Summarize the main findings including the strength of evidence for each main outcome; consider their relevance to key groups (e.g., healthcare providers, users, and policy makers).	19-22
Limitations	25	Discuss limitations at study and outcome level (e.g., risk of bias), and at review-level (e.g., incomplete retrieval of identified research, reporting bias).	22-23
Conclusions	26	Provide a general interpretation of the results in the context of other evidence, and implications for future research.	23
FUNDING			
Funding	27	Describe sources of funding for the systematic review and other support (e.g., supply of data); role of funders for the systematic review.	Not applicable

The PRISMA for Abstracts checklist

TITLE	CHECKLIST ITEM	REPORTED ON PAGE #
1. Title:	Identify the report as a systematic review, meta-analysis, or both.	1
BACKGROUND		
2. Objectives:	The research question including components such as participants, interventions, comparators, and outcomes.	1
METHODS		
3. Eligibility criteria:	Study and report characteristics used as criteria for inclusion.	1
4. Information sources:	Key databases searched and search dates.	1
5. Risk of bias:	Methods of assessing risk of bias.	1
RESULTS		
6. Included studies:	Number and type of included studies and participants and relevant characteristics of studies.	1
7. Synthesis of results:	Results for main outcomes (benefits and harms), preferably indicating the number of studies and participants for each. If meta-analysis was done, include summary measures and confidence intervals.	1
8. Description of the effect:	Direction of the effect (i.e. which group is favoured) and size of the effect in terms meaningful to clinicians and patients.	Not applicable
DISCUSSION		
9. Strengths and Limitations of evidence:	Brief summary of strengths and limitations of evidence (e.g. inconsistency, imprecision, indirectness, or risk of bias, other supporting or conflicting evidence)	1
10. Interpretation:	General interpretation of the results and important implications	1
OTHER		
11. Funding:	Primary source of funding for the review.	None
12. Registration:	Registration number and registry name.	PROSPERO CRD42017076736

Appendix S2: Search strategy

P (population) – All physicians in the Eastern Mediterranean Region; I (intervention) – Not applicable; C (comparator) – Not applicable; O (outcome) – Burnout and its sub-components as measured by Maslach Burnout Inventory; T (timing) – up to December 2019; S (setting) – Any setting in any of the Eastern Mediterranean Region countries.

Search strategy for Pubmed 30 June 2020

Records identified: 502 – No filter / No language and time restrictions

((("Burnout, Psychological"[Mesh] OR Burn-out[Text Word] OR "Burn out"[Text Word] OR Burnout[Text Word] OR "Caregiver Exhaustion"[Text Word] OR depersonalisation[Text Word] OR "personal accomplishment"[Text Word] OR "emotional exhaustion"[Text Word])) AND (("Qatar"[Mesh] OR "Bahrain"[Mesh] OR "Oman"[Mesh] OR "Saudi Arabia"[Mesh] OR "Kuwait"[Mesh] OR "United Arab Emirates"[Mesh] OR "Yemen"[Mesh] OR "Egypt"[Mesh] OR "Jordan"[Mesh] OR "Lebanon"[Mesh] OR "Syria"[Mesh] OR "Iraq"[Mesh] OR "Iran"[Mesh] OR "Somalia"[Mesh] OR "Afghanistan"[Mesh] OR "Libya"[Mesh] OR "Morocco"[Mesh] OR "Tunisia"[Mesh] OR "Djibouti"[Mesh] OR "Sudan"[Mesh] OR "South Sudan"[Mesh] OR "Pakistan"[Mesh] OR "Africa, Northern"[Mesh] OR "Africa, Eastern"[Mesh] OR "middle east"[Mesh] OR "Arabs"[Mesh] OR "UAE"[Text Word] OR "U.A.E"[Text Word] OR Emirat*[Text Word] OR "United Arab Emirates"[Text Word] OR Qatar*[Text Word] OR Oman*[Text Word] OR (Saudi[Text Word] AND Arabia*[Text Word]) OR Saudi*[Text Word] OR Kuwait*[Text Word] OR Bahrain*[Text Word] OR Yemen*[Text Word] OR Egypt*[Text Word] OR Jordan*[Text Word] OR Leban*[Text Word] OR Syria*[Text Word] OR Iraq*[Text Word] OR "West Bank"[Text Word] OR Gaza*[Text Word] OR Palestin*[Text Word] OR Iran*[Text Word] OR Somali*[Text Word] OR Afghan*[Text Word] OR Libya*[Text Word] OR Morocc*[Text Word] OR Tunis*[Text Word] OR Djibouti*[Text Word] OR Sudan*[Text Word] OR South Sudan*[Text Word] OR Pakistan*[Text Word] OR (North[Text Word] AND Africa*[Text Word]) OR North-Africa*[Text Word] OR ("Africa"[Text Word] AND "Northern"[Text Word]) OR "Northern Africa"[Text Word] OR "East Africa"[Text Word] OR ("Africa"[Text Word] AND "Eastern"[Text Word]) OR "Maghreb"[Text Word] OR "Maghrib"[Text Word] OR Arab*[Text Word] OR Bedouin*[Text Word] OR "Gulf Cooperation Council"[Text Word] OR "GCC"[Text Word] OR "Middle East"[Text Word])))

Search strategy for Embase 30 June 2020

Records identified: - 523 No filter / No language and time restrictions

(Burnout.mp. or exp burnout/ or Burn-out.mp. Or Burn out.mp. Or Caregiver Exhaustion.mp. Or depersonalisation.mp. Or personal accomplishment.mp. Or emotional exhaustion.mp.) AND (exp Middle East/ or exp North Africa/ or exp Arab/ or exp Djibouti/ or exp Pakistan/ or exp Sudan/ or exp South Sudan/ or exp Iran/ or exp Afghanistan/ or exp Somalia/ or exp Bahrain/ or exp Egypt/ or exp Jordan/ or exp Kuwait/ or exp Lebanon/ or exp Libya/ or exp Iraq/ or exp Morocco/ or exp Oman / or exp Qatar/ or exp Saudi Arabia/ or exp Syria / or exp Tunisia / or exp United Arab Emirates/ or exp Yemen/ or exp Palestine or Middle East.mp. or North Africa.mp. or EMRO.mp. or Eastern Mediterranean.mp. or Arab.mp. or Arabs.mp. or Arab World.mp. or Iran*.mp. or Afghan*.mp. or Somali*.mp. or Bahrain*.mp. or Djibouti.mp. or Egypt*.mp. or Jordan*.mp. or Kuwait*.mp. or Leban*.mp. or Libya*.mp. or Iraq*.mp. or Morocc*.mp. or Oman*.mp. or Pakistan*.mp. or Qatar*.mp. or Saudi*.mp. or Sudan*.mp. or Syria*.mp. or Tunisia*.mp. or United Arab Emirates.mp. or Emirat*.mp. or West Bank.mp. or Ghaza*.mp. or Gaza*.mp. or Palestin*.mp. or Yemen*.mp. or UAE.mp. or KSA.mp.)

Search strategy for Psycinfo 30 June 2020

Records identified 598: – No filter / No language and time restrictions

(DE "Arabs" OR MA qatar OR bahrain OR oman OR "Saudi arabia" OR saudi OR Kuwait OR Arabia* OR "united arab emirates" OR yemen OR egypt OR jordan OR lebanon OR syria OR Iraq OR libya OR morocco OR tunisia OR djibouti OR sudan OR "South sudan" OR pakistan OR "North africa" OR "northern africa" OR "east africa" OR "eastern africa" OR bedouin OR maghreb OR maghrib OR "gulf cooperation council" OR GCC OR SU qatar OR bahrain OR oman OR "Saudi arabia" OR saudi OR Kuwait OR Arabia* OR "united arab emirates" OR yemen OR egypt OR jordan OR lebanon OR syria OR Iraq OR Iran OR Afghanistan OR Somalia OR libya OR morocco OR tunisia OR djibouti OR sudan OR "South sudan" OR pakistan OR "North africa" OR "northern africa" OR "east africa" OR "eastern africa" OR bedouin OR maghreb OR maghrib OR "gulf cooperation council" OR GCC) OR (TX UAE OR TX U.A.E OR TX Emirat* OR TX United Arab Emirates OR TX Qatar* OR TX Oman* OR TX Saudi Arabia* OR TX Saudi* OR TX Kuwait* OR TX Bahrain* OR TX Yemen* OR TX Egypt* OR TX Jordan* OR TX Leban* OR TX Syria* OR TX Iraq* OR TX West Bank OR TX Gaza* OR TX Palestin* OR TX Iran* OR TX Somali* OR TX Afghan* OR TX Libya* OR TX Morocc* OR TX Tunis* OR TX Djibouti* OR TX Sudan* OR TX South Sudan* OR TX Pakistan* OR TX North Africa* OR TX North-Africa* OR TX "Northern Africa" OR TX "East Africa" OR TX "Eastern Africa" OR TX Maghreb OR TX Maghrib OR TX Arab* OR TX Bedouin* OR TX "Gulf Cooperation Council" OR TX GCC OR TX "Middle East") OR (TX UAE OR TX U.A.E OR TX Emirat* OR TX United Arab Emirates OR TX Qatar* OR TX Oman* OR TX Saudi Arabia* OR TX Saudi* OR TX Kuwait* OR TX Bahrain* OR TX Yemen* OR TX Egypt* OR TX Jordan* OR TX Leban* OR TX Syria* OR TX Iraq* OR TX West Bank OR TX Gaza* OR TX Palestin* OR TX Libya* OR TX Morocc* OR TX Tunis* OR TX Djibouti* OR TX Sudan* OR TX South Sudan* OR TX Pakistan* OR TX North Africa* OR TX North-Africa* OR TX "Northern Africa" OR RX "East Africa" OR TX "Eastern Africa" OR TX Maghreb OR TX Maghrib OR TX Arab* OR TX Bedouin* OR TX "Gulf Cooperation Council" OR TX GCC OR TX "Middle East") AND (DE "Occupational Stress" OR DE "Compassion Fatigue" OR TX "Compassion fatigue" OR burnout OR "burn out" OR burn-out OR "burn-out" OR "Caregiver Exhaustion" OR depersonalisation OR depersonalization OR "personal accomplishment" OR "emotional exhaustion")

Table S3: Characteristics of included studies

Study No	Author Name	Year of Publication	Study Quality	Country	Study Period	Response Rate (%)	Sample Size	Gender (%)	Mean Age (yrs)
1	Abbas, Ahmad [1]	2019	Good	Egypt	1 April - 30 Oct 2017	NA	147	NA	29.47± 5.49
2	AbdAllah, AM [2]	2019	Poor	Egypt	Jan 2017 - Apr 2018.	NA	168	M: 58.3<br>F: 41.7	27.46 ± 0.82
3	Abdo, SA [3]	2016	Good	Egypt	01 Nov- 30 Apr 2013	89.8	239	M: 27.7<br>F: 72.3	31.0 ± 6.0
4	Abdulaziz, Salman [4]	2009	Poor	Saudi Arabia	May-2015	NA	205	M: 65.85<br>F: 34.15	NA
5	Abdulrahman, Mahera [5]	2018	Poor	United Arab Emirates	May -Dec 2016	68	302	M: 21<br>F: 79	NA
6	Abyad, Abdulrazak [6]	2016	Poor	Middle East (Iran, Saudi Arabia, Kuwait, Lebanon, Iraq,)	NA	71	500	M: 54<br>F: 46	47.4
7	Agha, Adnan [7]	2014	Fair	Saudi Arabia	Aug -Oct 2012	NR	96	M: 66.7<br>F: 33.3	NA
8	Ahmadpanah, Mohammad [8]	2015	Fair	Iran	Jan-Dec 2011	NA	100	M: 71<br>F: 29	32±5.06

9	Al Dabbagh, Abdul Munem [9]	2019	Fair	Iraq	Apr-June 2017	95.7	134	M: 35.1<br>F: 64.8	NA
10	A Al Subhi, Abdulrahman [10]	2020	Good	Oman	Apr 2018 -Apr 2019	100	343	M: 37.9<br>F: 61.8	29
11	Al-Dubai,Sami Abdo Radman [11]	2010	Fair	Yemen	Dec 2006- July 2007	70.4	563	M: 59.5<br>F: 40.5	33.3 ±5.7
12	AL-Haddad, Ahmed [12]	2020	Fair	Saudi Arabia	Mar 2018-Aug 2019	80.7	226	M: 68.1<br>F: 31.9	NA
13	Al-Hashemi, Tharaya [13]	2019	Poor	Oman	Mar-May 2017	96	190	M: 21.6<br>F: 78.4	NA
14	Al-Sareai, N.S [14]	2013	Poor	Saudi Arabia	Oct 2010- Jun 2011	94.9	370	M: 81.9<br>F: 18.1	39.8
15	Al-Youbi, Reem A [15]	2013	Poor	Saudi Arabia	3 consecutive months in 2010	65	130	F: 55<br>M: 58	30 ± 5
16	Aldrees, Turki [16]	2013	Good	Saudi Arabia	Oct-Nov 2010	74	348	M: 72<br>F: 28	35
17	Aldrees, Turki [17]	2015	Fair	Saudi Arabia	Mar-May 2013	69	85	M: 67<br>F: 33	29

18	Aldress, Turki [18]	2017	Fair	Saudi Arabia	Apr-2015	60	38	M: 74<br>F: 26	28 ±1.9
19	Alhaffar, Bahaa Aldin [19]	2019	Fair	Syria	NA	87.3	3350	F: 44.10<br>M: 55.9	NA
20	Alotaibi, Abdulaziz Khalid [20]	2019	Poor	Saudi Arabia	Jan-2018	70	117	M: 53.85<br>F: 46.15	NA
21	Alqahtani, Abdulghani M [21]	2019	Poor	Saudi Arabia	NA	NA	95	NA	NA
22	Alsaawi, Abdulmoshen [22]	2014	Fair	Saudi Arabia	NA	74	53	M: 85<br>F: 15	NA
23	Alsaawi, Abdulmoshen [23]	2019	Fair	Saudi Arabia	2015	60.8	265	M: 84.2<br>F: 15.8	NA
24	Alsheikh, Khalid A [24]	2019	Poor	Saudi Arabia	Sept- Oct 2018	47.2	142	M: 90.1<br>F: 9.9	28.4 ±2.5
25	Alyamani, Alwaleed [25]	2018	Poor	Saudi Arabia	15 May 2018-31 May 2018	66.67	200	M: 60<br>F: 40	28
26	Arvandi, Zeinab [26]	2016	Poor	Iran	NA	NA	143	M: 50.3<br>F: 49.6	46.7±6.7
27	Ashkar, Khalil [27]	2010	Poor	Lebanon	20 May-20 Jun 2008	75	155	M: 55.5<br>F: 44.5	NA
28	Bany Hamdan, Abdullah [28]	2019	Fair	Saudi Arabia	June 2016-June 2017	62.8	55	M: 39.5<br>F: 60.5	NA

29	Belayachi, Jihane [29]	2016	Poor	Morocco	April-Oct 2010	NA	198	M: 41.4<br>F: 58.6	29.3 ±3.2
30	Ben Zid, A; 2018 [30]	2018	Good	Tunisia	<2017	76.8	149	F: 58.4<br>M: 41.6	29
31	Benbrahim, Zineb; 2019 [31]	2019	Poor	Morocco	Jan-18	25	100	M: 25<br>F: 75	NA
32	Bin Dahmash, Abdulmajeed; 2019 [32]	2019	Poor	Saudi Arabia	Mar-18	83.8	31	M: 55.2<br>F: 44.8	NA
33	Chaudhruy,Muhammad Ashraf; 2015 [33]	2015	Poor	Pakistan	Mar - Sep 2014.	NA	133	M: 49.6<br>F: 50.4	35.10 ± 11.16.
34	El Kettani, Assiya; 2017 [34]	2017	Good	Morocco	NA	63.7	191	M: 20.9<br>F: 79.1	26.7
35	Fauzia, A Khan; 2019 [35]	2019	Good	Pakistan	2017	74.5	447	M: 66.9<br>F: 33.1	NA
36	Farahat, Taghreed Mohamed; 2016 [36]	2016	Poor	Egypt	Aug 2014 - Apr 2015	91.6	76	M: 47<br>F: 53	33.7
37	Farahat, Tagraeed Mohamed; 2017 [37]	2017	Poor	Egypt	Aug 2014 -May 2015	91.6	76	M: 52.6<br>F: 47.4	33.7
38	Ghaida M.J, Al-Shoraian; 2011 [38]	2011	Poor	Kuwait	Aug 2010-Mar2011	52.9	200	M: 44<br>F: 56	NA
39	Hamdan, Motasem; 2017 [39]	2017	Fair	Palestine	Jul-Sept 2013	74.5	142	M: 76.8<br>F: 27.3	NA
40	Hameed, Tahir Kamal; 2018 [40]	2018	Fair	Saudi Arabia	2013-2014	43	181	M: 41.4<br>F: 58.6	NA
41	Hasan, Husain Isa; 2015 [41]	2015	Good	Bahrain	NA	87.7	202	M: 60.7<br>F: 39.9	30-40 ±9
42	Hussein, H. Y; 2015 [42]	2015	Poor	United Arab Emirates	NA	NA	102	M: 29.4<br>F: 70.6	NA

43	I, Kacem; 2017[43]	2017	Good	Tunisia	2011	52.6	342	M: 47.4<br>F: 52.6	NA
44	Jadoo, Saad Ahmed Ali [44]	2018	Poor	Iraq	Jan-Jun2014	87.3	576	M: 46.2<br>F: 53.8	40.43 ±8.59
45	Jalili, Mohammad [45]	2013	Poor	Iran	Mar- Apr 2008	88	165	M: 91.5<br>F: 8.5	33.6
46	Jamjoom, Roaa S [46]	2018	Poor	Saudi Arabia	May-16	64	32	M: 6.3<br>F: 93.7	NA
47	Khalil, Ashkar [47]	2018	Poor	Lebanon	20 May-20 Jun 2008	75	155	M: 55.5<br>F: 44.5	NA
48	Kalani, Simin Dokht [48]	2017	Unclear	Iran	NA		104	NA	NA
49	Khan, Khadija Asim [49]	2020	Poor	Pakistan	Jan-Jun 2017	NA	55	M: 7.3<br>F: 92.7	44.89 ± 8.50
50	Kotb, Amany Ali [50]	2014	Poor	Egypt	NA	NA	171	M: 44.44<br>F: 55.56	NA
51	Liaqat, Naeem [51]	2019	Poor	Pakistan	Jan- Feb 2017	36.6	40	M: 85<br>F: 15	44.89 ± 8.50
52	Mohamed, Alorabi [52]	2019	Poor	Egypt	NA	90	47	M: 44.7<br>F: 55.3	NA
53	M, Oumaya [53]	2009	Unclear	Tunisia	NA	85.6	113	NA	NA
54	M, Taei [54]	2010	Unclear	Iran	2009	NA	150	NA	NA
55	Mohammed, Almhana Abdulhussein Abdulzahra [55]	2019	Unclear	Iraq	NA	84	296	NA	NA

56	Malik, Awais Amjad [56]	2016	Poor	Pakistan	NA	66.5	133	M: 73.7<br>F: 26.3	NA
57	Marzouki, Hani [57]	2019	Poor	Saudi Arabia	Decr 2016 - Jun 2017	77	77	NA	43.8 ± 9.2
58	Massou, Salah [58]	2013	Good	Morocco	2009	NA	154	NA	NA
59	Mazhar, Syeda Batool; 2019 [59]	2019	Good	Pakistan	Oct 2016- Jan 2017	100	365	M: 65<br>F: 35	28.68 ± 4.58
60	Mhamdi, Salah; 2018 [60]	2018	Good	Tunisia	2014	72.19	35	NA	40.83
61	Moein, Neda; 2018 [61]	2018	Unclear	Iran	2017-2018	NA	155	M: 29<br>F: 71	35.3±8.5
62	Mohammed,Khalid Abdul-Moez; 2014 [62]	2014	Poor	Egypt	2012	64.6	84	M: 54.8	NA
63	Mohammed, Sura Bahjat; 2018 [63]	2018	Poor	Iraq	2016	92	310	M: 49.7<br>F: 50.3	34.75 ± 7.5
64	Mortada, Eman M; 2012 [64]	2012	Poor	Egypt	Jan- April 2012	NA	250	M: 53.6<br>F: 46.4	36.9 ± 8.9
65	Ok, Abd EL Latief; 2018[65]	2018	Poor	Egypt	Oct- Dec 2016	NA	134	NA	39.45± 10.30
66	Omr, Amr S; 2019 [66]	2019	Poor	Qatar	NA	NA	65	NA	NA
67	Osman, Doaa MM; 2019 [67]	2019	Poor	Egypt	May-July 2017	86*	NA	NA	NA
68	Sadat-Ali, Mir; 2005 [68]	2005	Poor	Saudi Arabia	Sept 2003-Oct 2004	67.6	69	NA	45.72 ± 6.82
69	Salem, Mohamed; 2018 [69]	2018	Poor	Qatar	NA	90	144	M: 64.29<br>F: 35.71	NA

70	Salem, Rana; 2018 [70]	2018	Good	Lebanon	NA	73	51	M: 68.6<br>F: 31.4	NA
71	Selaihem, Ahmed Abdullah [71]	2013	Poor	Saudi Arabia	April 2010.	72	144	M: 61.1<br>F: 38.9	37.08 ± 8.48
72	Shams, Tarek [72]	2013	Poor	Egypt	Mar-June 2011	73	134	M: 73.5<br>F: 26.5	NA
73	Sheikhmoonesi, Fatemeh [73]	2017	Poor	Iran	2012	60	106	M: 50.9<br>F: 49.10	NA
74	Sherbiny, E -E [74]	2017	Poor	Saudi Arabia	July-Aug 2014	80	140	M: 31.4<br>F: 68.6	NA
75	Soltanifar, Atefeh [75]	2018	Fair	Iran	Dec 2016 - Jan 2017	75	77	F: 100	NA
76	Yousef, I. M [76]	2006	Good	Egypt	NA	72.4	84	M: 76.2<br>F: 23.8	NA
77	Zarei, Ehsan [77]	2019	Fair	Iran	2018	NA	66	NA	NA
78	Zubairi, Akbar Jaleel [78]	2016	Poor	Pakistan	1st -31st May 2013	34	82	M:54<br>F: 46	NA

M-Male **F-**Female **N/A**-Not available

Appendix S4: List of excluded studies

1. Abarghouei MR, Sorbi MH, Abarghouei M, Bidaki R, Yazdanpoor S. A study of job stress and burnout and related factors in the hospital personnel of Iran. Electronic physician. 2016;8(7):2625-2632
2. Abdulla L, Al-Qahtani DM, Al-Kuwari MG. Prevalence and determinants of burnout syndrome among primary healthcare physicians in Qatar. South African Family Practice. 2011;53(4):380-383.
3. Abdulghani HM, Al-Harbi MM, Irshad M. Authors' reply: Financial stress during medical residency training: An experience from Iran. Neuropsychiatric Disease and Treatment. 2016;12:489.
4. Abdulghafour YA, Bo-hamra AM, Al-Ri MS, Kamel MI, El-Shazly MK. Burnout syndrome among physicians working in primary health care centers in Kuwait. Alexandria Journal of Medicine. 2011;47(4):351-357.
5. Abu-Mughaiseeb A, Ali MH, Al Zarrad F. Burn-out syndrome Synonym: Al-Ofool syndrome Work- related stress in health workers and others. Arab Journal of Psychiatry. 2002;13(2):118-121.
6. Adler AB, Adrian A, L a, et al. Professional Stress and Burnout in U.S. Military Medical Personnel Deployed to Afghanistan. Military medicine. 2017;182(3):e1669-e1676.
7. Afana A, Ghannam J, Ho EY, Al-Khal A, Al-Arab B, Bylund CL. Burnout and sources of stress among medical residents at Hamad Medical Corporation, Qatar. Eastern Mediterranean health journal = La revue de sante de la Mediterranee orientale = al-Majallah al-sihhiyah li-sharq al-mutawassit. 2017;23(1):40-45.
8. Agoub M, Elyazaji M, Battas O. Épuisement professionnel et sources de stress au travail chez les soignants - Professional burn out and sources of stress among health care workers. Annales Médico-Psychologiques. 2000;158(9):687-692.
9. Ahmad W, Ashraf H, Talat A, et al. Association of burnout with doctor-patient relationship and common stressors among postgraduate trainees and house officers in Lahore-a cross-sectional study. PeerJ. 2018;6:e5519.
10. Ahmadi SAA, Ahmadi F, Tavreh N. The relationship between organizational citizenship behavior and burnout in public organization in west Azarbayejan province. Interdisciplinary Journal of Contemporary Research in Business. 2011;2(11):147-156.
11. Ahmady S, Changiz T, Masiello I, Brommels M. Organizational role stress among medical school faculty members in Iran: dealing with role conflict. BMC medical education. 2007;7:14.
12. Ahmed I, Banu H, Al-Fageer R, Al-Suwaidi R. Cognitive emotions: depression and anxiety in medical students and staff. Journal of critical care. 2009;24(3):e1-7.
13. Aksoy DY, Durusu Tanriover M, Unal S, et al. Burnout syndrome during residency in internal medicine and pediatrics in a country without working time directive. International journal of health care quality assurance. 2014;27(3):223-230.
14. Al Breiki MH, Al Muqbali M. Well-being among medical students in clinical years at a private college in Oman: Cross sectional study (7526). Swiss Archives of Neurology, Psychiatry and Psychotherapy. 2019;70:50S
15. Alkhalifa F, Abdul Raheem F, Hassan Y, Al-Saddah J, Alabbad J. Comparison of burnout among surgery residents and attending surgeons. Diseases of the Colon and Rectum. 2019;62(6):e113-e114
16. Al Nuhait M, Al Harbi K, Al Jarboa A, et al. Sickness presenteeism among health care providers in an academic tertiary care center in Riyadh. Journal of infection and public health. 2017;10(6):711-715.
17. Al Ubaidi et al. A Study on the Prevalence of Burnout Among Primary Care Physicians on the Kingdom of Bahrain. Journal of the Bahrain Medical Society. 2020; 32(2):8-16

18. Al-Alawi M, Al-Sinawi H, Al-Qubtan A, et al. Prevalence and determinants of burnout Syndrome and Depression among medical students at Sultan Qaboos University: A cross-sectional analytical study from Oman. Archives of environmental & occupational health. 2019;74(3):130-139.
19. Al-Dubai SAR, Rampal KG. Psychological morbidity and sources of job stress among doctors in Yemen. ASEAN Journal of Psychiatry. 2012;13(1):20-30.
20. Al-Farah A.Psychological burnout among professionals serving individuals with special needs in the state of Qatar. Dirasat: Educational Sciences. 2001;28(2):247-271.
21. Al-Janabi O, Barber J, Boggess B, O’Connor K, et al. Burnout rate among Iraqi neurology residents. Neurology. 2019;92(15).
22. Al-Kuwari RS. Nationality, gender, and sector differences on work-related stress in white-collar employees in Qatar, ProQuest Information & Learning; 2015.
23. Al-lozi M, Al-Hunitie N. The Effect of Professional and Personal Factors on Occupational Stress at Governmental Hospitals in Jordan. Dirasat: Administrative Sciences. 2003;30(2):350-370.
24. Al-Mendalawi MD. AO - Al-Mendalawi MD. Assessment of pediatric residents burnout in a tertiary academic centre(1). Saudi Medical Journal. 2018;39(5):528.
25. Al-Otaibi AG. The effects of job stress on psychosomatic disorders and absenteeism among government employees in Kuwait. Journal of the Social Sciences. 1997;25(2):177-201.
26. Al-Rabiah FA. Sources of counselor burnout in Saudi Arabian mental health settings, ProQuest Information & Learning; 1988.
27. Al-Shammari SA, Khoja TA, Al-Subaie AS. Job satisfaction and occupational stress among primary health care center doctors. International Journal of Mental Health. 1996;24(4):85-95.
28. Al-Youbi RA, Kan MM. Burnout syndrome in pediatric practice. Annals of Neurology. 2013;74:S136.
29. Alameddine M, Baroud M, Kharroubi S, et al. Investigating the job satisfaction of healthcare providers at primary healthcare centres in Lebanon: A national cross-sectional study. Health & social care in the community. 2017;25(6):1805-1816.
30. Alameddine M, Kazzi A, El-Jardali F, Dimassi H, Maalouf S. Occupational violence at Lebanese emergency departments: prevalence, characteristics and associated factors. Journal of occupational health. 2011;53(6):455-464.
31. Alameddine M, Saleh S, El-Jardali F, Dimassi H, Mourad Y. The retention of health human resources in primary healthcare centers in Lebanon: a national survey. BMC health services research. 2012;12:419.
32. Ali FS, Zuberi BF, Rasheed T, Shaikh MA. Why doctors are not satisfied with their job-current status in tertiary care hospitals. Pakistan journal of medical sciences. 2019;35(1):205-210.
33. Almadi T, Cathers I, Mansour AMH, Chow CM. The association between work stress and inflammatory biomarkers in Jordanian male workers. Psychophysiology. 2012;49(2):172-177.
34. Almalki SA, Almojali AI, Alothman AS, Masuadi EM, Alaqeel MK. Burnout and its association with extracurricular activities among medical students in Saudi Arabia. International journal of medical education. 2017;8:144-150.
35. Almehdar AS. Direct and indirect effects of dispositional gratitude on physicians' professional quality of life and family satisfaction, ProQuest Information & Learning; 2019.
36. Almutairi AF, Salam M, Adlan AA, Alturki AS. Prevalence of severe moral distress among healthcare providers in Saudi Arabia. Psychology research and behavior management. 2019;12:107-115.
37. Alosaimi FD, Kazim AS, Almufleh AS, et al . Prevalence of stress and its determinants among residents in Saudi Arabia. Saudi Medical Journal. 2015;36(5):605-612
38. Altannir Y, Alnajjar W, Ahmad SO, et al. Assessment of burnout in medical undergraduate students in Riyadh, Saudi Arabia. BMC medical education. 2019;19(1):34.
39. Alvi SM, Ali SAZ, Jabeen F, Jabeen T, et al. Occupational stress as predictor of job satisfaction and burnout among doctors. Rawal Medical Journal. 2018;43(3):515-519.
40. Amamou B, Bannour AS, Ben Hadj Yahia M, Ben Nasr S, Ben Hadj Ali B. [High prevalence of burnout in the Tunisian units that support terminally ill patients]. The Pan African medical journal. 2014;19:9.
41. Amini A, Munesan MR, Kariman H, et al. Quality of Life in Emergency Medicine Specialists of Teaching Hospitals. Emergency (Tehran, Iran). 2014;2(3):134-137.
42. Amiri M, Khosravi A, Eghtesadi AR, et al. Burnout and its Influencing Factors among Primary Health Care Providers in the North East of Iran. PloS one. 2016;11(12):e0167648.

43. Anila A, Riaz W, L Ansar, Faisal Shabir. Analyzing stress amongst treating physicians of Sialkot- A cross sectional survey. Pakistan Journal of Medical and Health Sciences. 2018;12(3):993-995.
44. Anis-ul-Haque M, Khan S. Burnout and Organizational Sources of Social Support in Human Service Professions: A Comparison of Woman Doctors and Nurses. Journal of the Indian Academy of Applied Psychology. 2001;27(1):57-66.
45. Armon G, Melamed S, Shirom A, Shapira I. Elevated burnout predicts the onset of musculoskeletal pain among apparently healthy employees. Journal of occupational health psychology. 2010;15(4):399-408.
46. Armon G, Shirom A, Berliner S, Shapira I, Melamed S. A prospective study of the association between obesity and burnout among apparently healthy men and women. Journal of occupational health psychology. 2008;13(1):43-57.
47. Armon G, Shirom A, Shapira I, Melamed S. On the nature of burnout-insomnia relationships: a prospective study of employed adults. Vol 652008.
48. Arrizabalaga P, Valls-Llobet C. Female doctors: From incorporation to discrimination. Medicina Clinica. 2005;125(3):103-107.
49. Asghar AA, Faiq A, Shafique S, et al. Prevalence and Predictors of the Burnout Syndrome in Medical Students of Karachi, Pakistan. Cureus. 2019;11(6):e4879.
50. Ashtari Z, Farhady Y, Khodaee MR. Relationship between job burnout and work performance in a sample of Iranian mental health staff. African journal of psychiatry. 2009;12(1):71-74.
51. Avieli H, Ben-David S, Levy I. Predicting professional quality of life among professional and volunteer caregivers. Psychological trauma : theory, research, practice and policy. 2016;8(1):80-87.
52. Aziz AA, Kahlout BH, Bashir K. Female Physicians Suffer a Higher Burnout Rate: A 10-Year Experience of the Qatar EM Residency Training Programme. Journal of the College of Physicians and Surgeons--Pakistan : JCPSP. 2018;28(8):651-652.
53. Bahgat N. Burnout and depression in ICU staff members. Critical Care. 2018;22.
54. Bagherzadeh R, Taghizadeh Z, Mohammadi E, Kazemnejad A, Pourreza A, Ebadi A. Relationship of work-family conflict with burnout and marital satisfaction: cross-domain or source attribution relations? Health promotion perspectives. 2016;6(1):31-36.
55. Bahraini NH. Development and validation of the Professional Support Inventory for Mental Health Professionals, ProQuest Information & Learning; 2009.
56. Ball S. Strategies in genetic counseling: The challenge of the future, Vol 1. New York, NY: Human Sciences Press; 1988.
57. Bawakid K, Abdulrashid O. Burnout of Physicians Working in Primary Health Care Centers under Ministry of Health Jeddah, Saudi Arabia. Cureus. 2018; 11(9):e1877.
58. Belayachi J, Louriz M, Madani N, Amlaiky F, et al. Prevalence, characteristics, and associated factors to physician's conflicts in Emergency Department: A multicentric Moroccan study. Annals of Intensive Care. 2016;6
59. Ben-Zur H, Michael K. Burnout, social support, and coping at work among social workers, psychologists, and nurses: the role of challenge/control appraisals. Vol 452007.
60. Benight CC. Secondary trauma and burnout in military behavioral health providers: Beyond the battlefield. New York, NY: Palgrave Macmillan; 2016.
61. Bernat JL. How can neurologists avoid burnout? Neurology. 2017;88(8):726-727.
62. Bhatti AM. Massive brain drain of health force from Pakistan worrisome. Medical Forum Monthly. 2006;17(2):1
63. Bilal MS, Rana MH, Rahim S, Ali S. Psychological trauma in a relief worker--a case report from earthquake-struck areas of north Pakistan. 2007-Sep 2007. 1049-023X.
64. Boggs SD, Doll D, Luedi MM. Evaluation of Job Stress and Burnout Among Anesthesiologists Working in Academic Institutions in 2 Major Cities in Pakistan and the Global Perspective. Anesthesia and analgesia. 2019;129(2):e64.
65. Boran A, Shawaheen M, Khader Y, Amarin Z, Rice VH. Work-related stress among health professionals in northern Jordan. Occupational Medicine. 2012;62(2):145-147.
66. Brahem A, Ilahi S, Maoua M, et al. Syndrome dépressif et absentéisme chez le personnel soignant de la région de Sousse (Tunisie) = Depressive syndrome and absenteeism among health personnel in Sousse (Tunisia). Annales Médico-Psychologiques. 2016;174(7):557-563.
67. Burrows GD, Stanley R. Editorial. Stress and Health: Journal of the International Society for the Investigation of Stress. 2006;22(5):283-283.
68. Carmel S, Glick SM. Compassionate-empathic physicians: personality traits and social-organizational factors that enhance or inhibit this behavior pattern. Social science & medicine (1982). 1996;43(8):1253-1261.

69. Cevik AA, Holliman CJ, Yanturali S. [Emergency physicians and "burn out" syndrome]. Ulusal travma ve acil cerrahi dergisi = Turkish journal of trauma & emergency surgery : TJTES. 2003;9(2):85-89.
70. Chakroun WO, Rejeb I, Kammoun L, et al. [Evaluation of stress among emergency staff: survey in a Tunisian emergency department]. Vol 322013.
71. Charkhabi M. Do cognitive appraisals moderate the link between qualitative job insecurity and psychological-behavioral well-being? International Journal of Workplace Health Management. 2018;11(6):424-441.
72. Charkhabi M. Quantitative job insecurity and well-being: Testing the mediating role of hindrance and challenge appraisals. Frontiers in Psychology. 2019;9.
73. Chemali Z, Smati H, Johnson K, Borba CPC, Fricchione GL. Reflections from the Lebanese field: "First, heal thyself". Conflict and health. 2018;12:8.
74. Cicognani E, Pietrantoni L, Palestini L, Prati G. Emergency workers' quality of life: The protective role of sense of community, efficacy beliefs and coping strategies. Social Indicators Research. 2009;94(3):449-463.
75. Dachraoui F, Mghirbi A, Adhieb A, et al. Anxiety and depression in critial care staff of the university hospital of Monastir, Tunisia. Annals of Intensive Care. 2017;7(1):136.
76. de Beer L, Rothmann SJ, Pienaar J. A confirmatory investigation of a job demands-resources model using a categorical estimator. Psychological Reports. 2012;111(2):528-544.
77. de Beer LT, Pienaar J, Rothmann SJ. Work overload, burnout, and psychological ill-health symptoms: A three-wave mediation model of the employee health impairment process. Anxiety, Stress & Coping: An International Journal. 2016;29(4):387-399.
78. De Witte H, Pienaar J, De Cuyper N. Review of 30 years of longitudinal studies on the association between job insecurity and health and well-being: Is there causal evidence? Australian Psychologist. 2016;51(1):18-31.
79. Dehghan A, Hosseini SA, Rassfiani M, Dalv, H. Exploring perceptions of health caregivers on the causes of caregivers' occupational burnout in institutes of children with cerebral palsy: A qualitative study. Electronic physician. 2017;9(6):4516-4523.
80. Dehkardi MA, Azr, i AA, Hosseinai A. Middle age burnout: A potential development event. Developmental Psychology: Journal of Iranian Psychologists. 2012;8(31):279-290.
81. Demirci S, Yildirim YK, Ozsaran Z, Uslu R, Yalman D, Aras AB. Evaluation of burnout syndrome in oncology employees. Vol 272010.
82. Deosthalee PG. Are Indian expatriates in Sultanate of Oman under stress? Journal of Managerial Psychology. 2002;17(6):523-528.
83. Derbas A, Jahrami H, Al-Haddad M, Qaheri S. Survey of burnout amongst drug and alcohol unit staff. Bahrain Medical Bulletin. 2013;35(1):1-9.
84. Dewa CS, Loong D, Bonato S, Trojanowski L. The relationship between physician burnout and quality of healthcare in terms of safety and acceptability: a systematic review. BMJ open. 2017;7(6):e015141.
85. Dhillon GS, Dabbo S, Flower R, Adhikari N. Critical care in the developing world: A literature review. American Journal of Respiratory and Critical Care Medicine. 2010;181(1).
86. Dhahi SA. Prevalence of burnout among home health care staff members in Saudi Arabia, 2010. European Geriatric Medicine. 2012;3:S66
87. Doghmi N, Massou S, Balkhi H, Haimeur C, Drissi Kamili N. Le burn out, conséquences et solutions: Enquête chez les personnels d'anesthésie–réanimation de quatre hôpitaux universitaires marocains = The burn out, consequences and resolutions: Inquiry at the personnels of anaesthesia intensive care of four Moroccan university hospitals. Annales Médico-Psychologiques. 2013;171(10):700-704.
88. Dugani S, Afari H, Hirschhorn LR, et al. Prevalence and factors associated with burnout among frontline primary health care providers in low- and middle-income countries: A systematic review. Vol 22018.
89. Ebadi A, Froutan R, Malekzadeh J. The design and psychometric evaluation of the emergency medical services resilience scale (EMSRS). International emergency nursing. 2019;42:12-18.
90. Elayyan M, Rankin J, Chaarani MW. Factors affecting empathetic patient care behaviour among medical doctors and nurses: an integrative literature review. Eastern Mediterranean health journal = La revue de sante de la Mediterranee orientale = al-Majallah al-sihhiyah li-sharq al-mutawassit. 2018;24(3):311-318.
91. Elbarazi I, Loney T, Yousef S, Elias A. Prevalence of and factors associated with burnout among health care professionals in Arab countries: a systematic review. BMC health services research. 2017;17(1):491.

92. Elshaer NSM, Moustafa MSA, Aiad MW, Ramadan MIE. Job Stress and Burnout Syndrome among Critical Care Healthcare Workers. Alexandria Journal of Medicine. 2018;54(3):273-277.
93. Eriksson CB, Bjorck JP, Larson LC, et al. Social support, organisational support, and religious support in relation to burnout in expatriate humanitarian aid workers. Mental Health, Religion & Culture. 2009;12(7):671-686.
94. Ettner R, White T, Brown GR. Family and systems aggression toward therapists. International Journal of Transgenderism. 2010;12(3):139-143.
95. Essadi Z, Belbaraka R, Aloulou A, et al. Burnout in health caregivers: Experience of a Moroccan anti cancer center. European Journal of Cancer. 2013;49:S389-S390.
96. Fares J, Saadeddin Z, Al Tabosh H, et al. Extracurricular activities associated with stress and burnout in preclinical medical students. Journal of epidemiology and global health. 2016;6(3):177-185.
97. Firoozabadi A, Uitdewilligen S, Zijlstra FRH. Should you switch off or stay engaged? The consequences of thinking about work on the trajectory of psychological well-being over time. Journal of Occupational Health Psychology. 2018;23(2):278-288.
98. Fischer R, Van de Vliert E. Does climate undermine subjective well-being? A 58-nation study. Personality and Social Psychology Bulletin. 2011;37(8):1031-1041.
99. Fogarty L, Kim YM, Juon H-S, et al. Job satisfaction and retention of health-care providers in Afghanistan and Malawi. Human resources for health. 2014;12:11.
100. Frajerman A, Morvan Y, Krebs M-O, Gorwood P, Chaumette B. Burnout in medical students before residency: A systematic review and meta-analysis. European psychiatry : the journal of the Association of European Psychiatrists. 2019;55:36-42.
101. Froutan R, Khankeh HR, Fallahi M, Ahmadi F, Norouzi K. Pre-hospital burn mission as a unique experience: a qualitative study. Burns : journal of the International Society for Burn Injuries. 2014;40(8):1805-1812.
102. Gashmard R, Bagherzadeh R, Pouladi S, Akaberian S. Burnout and its related Demographic Factors among the Medical Staff working in Hospitals associated with Bushehr University of Medical Sciences. Puerto Rico health sciences journal. 2015;34(4):208-214.
103. Ghannam J, Afana A, Ho EY, Al-Khal A, Bylund CL. The impact of a stress management intervention on medical residents' stress and burnout. International Journal of Stress Management. 2019.
104. Ghazanfar H, Chaudhry MT, Asar ZU, Zahid U. Compassion Satisfaction, Burnout, and Compassion Fatigue in Cardiac Physicians Working in Tertiary Care Cardiac Hospitals in Pakistan. Cureus. 2018;10(10):e3416.
105. Ghezala HB, Bedoui N, Snouda S, Daoudi R, Kaddour M. The burnout syndrome in a Tunisian intensive care unit: What is the truth? Annals of Intensive Care. 2016;6.
106. Guveli H, Anuk D, Oflaz S, et al. Oncology staff: Burnout, job satisfaction and coping with stress. Psycho-Oncology. 2015;24(8):926-931
107. Hafeez S. Organizational status inconsistency and stress: A research note on women administrators in a developing society. Topics in Culture Learning. 1976;4:16-17.
108. Haghighi M, Gerber M. Does mental toughness buffer the relationship between perceived stress, depression, burnout, anxiety, and sleep? International Journal of Stress Management. 2019;26(3):297-305.
109. Halayem-Dhouib S, Zaghdoudi L, Zremdini R, Maalej I, Béchir MB, Labbène R. [Burnout among mental health professionals: A Tunisian experience]. Vol 582010.
110. Hamid AARM, Musa SA. The mediating effects of coping strategies on the relationship between secondary traumatic stress and burnout in professional caregivers in the UAE. Journal of mental health (Abingdon, England). 2017;26(1):28-35.
111. Hamidi Y, Eivazi Z. The relationships among employees' job stress, job satisfaction, and the organizational performance of Hamadan urban health centers. Social Behavior and Personality: An International Journal. 2010;38(7):963-968.
112. Haq Z, Iqbal Z, Rahman A. Job stress among community health workers: A multi-method study from Pakistan. International Journal of Mental Health Systems. 2008;2.
113. Hareli S, Karnieli-Miller O, Hermoni D, Eidelman S. Factors in the doctor-patient relationship that accentuate physicians' hurt feelings when patients terminate the relationship with them. Patient education and counseling. 2007;67(1):169-175.
114. Hassan-Bitar S, Narrainen S. 'Shedding light' on the challenges faced by Palestinian maternal health-care providers. Midwifery. 2011;27(2):154-159.
115. Iorio A, Palareti L, Poti S, Lapecorella M et.al. When health care professionals express themselves: Results overview of the qualitative phase of hero initiative. Blood Transfusion. 2016;14:s781

116.Jahrami H. A survey of burnout of the mental health occupational therapy staff in the Psychiatric Hospital, Bahrain. The British Journal of Occupational Therapy. 2009;72(10):458-464.
117.Jahrami H, Thomas A, Saif Z, et al. The relationship between burnout and job satisfaction among mental health workers in the psychiatric hospital, Bahrain. Arab Journal of Psychiatry. 2013;24(1):69-76.
118.Jamil A, Raja U, Darr W. Psychological contract types as moderator in the breach-violation and violation-burnout relationships. The Journal of psychology. 2013;147(5):491-515.
119.Javanshir E, Dianat I, Asghari-Jafarabadi M. Psychometric properties of the Iranian version of the Copenhagen Burnout Inventory. Health promotion perspectives. 2019;9(2):137-142.
120.Jordan P, Steingen U, Terschuren C, Harth V. The Maslach Burnout Inventory: A test dimensionality assessment via item response theory. TPM-Testing, Psychometrics, Methodology in Applied Psychology. 2018;25(1):101-120.
121.Kalboussi A, Chouchane A, Ferhi F, et al. Burnout Syndrome and its associated factors among Anesthesia Technicians in a Teaching Hospital in the central region of Tunisia. Annales Medico-Psychologiques. 2019
122.Kabir MJ, Heidari A, Etemad K, et al. Job Burnout, Job Satisfaction, and Related Factors among Health Care Workers in Golestan Province, Iran. Electronic physician. 2016;8(9):2924-2930.
123.Kacem I, Kalboussi H, Ayoub N, et al. burn-out chez les jeunes médecins: Étude réalisée dans la région de sousse = Burn-out among young doctors: Study conducted in the Sousse region. Annales Médico-Psychologiques. 2017;175(4):332-338.
124.Kakav MG, Mordi M, Charganeh SS, Yarmohammadi H. Studying the prevalence of occupational burnout in staff working at a hospital in the city of javanrood-Kermanshah (Iran). International Journal of Pharmacy and Technology. 2016;8(3):17891-17899.

125.Kalemoglu M, Keskin O. [Evaluation of stress factors and burnout in the emergency department staff]. Ulusal travma dergisi = Turkish journal of trauma & emergency surgery : TJTES. 2002;8(4):215-219.
126.Kazmi R, Amjad S, Khan D. Occupational stress and its effect on job performance. A case study of medical house officers of district Abbottabad. Journal of Ayub Medical College, Abbottabad : JAMC. 2008;20(3):135-139.
127.Keshvari M, Mohammadi E, Boroujeni AZ, Farajzadegan Z. Burnout: interpreting the perception of Iranian primary rural health care providers from working and organizational conditions. International journal of preventive medicine. 2012;3:S79-88.
128.Khaghanizadeh M, Vafadar Z, Salari M, Ebadi A. Effects of suggestions' system on decreasing job burn out among the staff. Journal of Psychology. 2008;12(2):213-226.
129.Khan J, Bernat JL. Preventing burnout increases the desirability of neurology as a career. Neurology. 2017;89(16):1658-1659.
130.Khan Ghazi E. Secondary trauma faced by health professionals giving end of life care to children with cancer in Pakistan. Pediatric Blood and Cancer. 2016;63:S227-S228
131.Khani PM. Study of job stress and job satisfaction in a group of psychologists at Iranian Welfare Organization. Psychological Research. 2008;10(3):99-110.
132.Khammarnia M. Letter to the editor. Australasian Medical Journal. 2014;7(11):445-447.
133.Khatiban M, Hosseini S, Bikmoradi A, Roshanaei G, Karampourian A. Occupational Burnout and Its Determinants among Personnel of Emergency Medical Services in Iran. Acta medica Iranica. 2015;53(11):711-716.
134.Khorshidian N, Hashemian S-S, Meftagh S-D, Najimi A. Burnout among the employees of health and therapy entities: Investigating the role of early maladaptive schemas and mental disorder symptoms. Anadolu Psikiyatri Dergisi. 2017;18(4):323-329.
135.Koenig C, Maguen S, Monroy J, Seal K.Compassion fatigue, professional burnout and self-care among multidisciplinary providers working in outpatient primary care settings. Journal of General Internal Medicine. 2013;28:S52-S53
136.Kortum E, Leka S, Cox T. Psychosocial risks and work-related stress in developing countries: Health impact priorities, barriers and solutions. International Journal of Occupational Medicine and Environmental Health. 2010;23(3):225-238.

137.Kotb AA, Mohamed KA-E, Kamel MH, Ismail MAR, Abdulmajeed AA. Evaluating the effect of an educational program on level of professional burnout among family physicians in the Faculty of Medicine—Suez Canal University. Arab Journal of Psychiatry. 2014;25(1):52-60.
138.Kotb AA, Mohamed KA-E, Kamel MH, Ismail MAR, Abdulmajeed AA. Comparison of burnout pattern between hospital physicians and family physicians working in Suez Canal University Hospitals. The Pan African medical journal. 2014;18:164.
139.Kurtz A. The needs of staff who care for people with a diagnosis of personality disorder who are considered a risk to others. Journal of Forensic Psychiatry & Psychology. 2005;16(2):399-422.
140.Kushnir T, Kushnir J, Sarel A, Cohen AH. Exploring physician perceptions of the impact of emotions on behaviour during interactions with patients. Family practice. 2011;28(1):75-81.
141.Kushnir T, Malkinson R, Ribak J. Teaching stress management skills to occupational and environmental health physicians and practitioners. A graduate-level practicum. Journal of occupational medicine : official publication of the Industrial Medical Association. 1994;36(12):1335-1340.
142.Maaroufi N, Zeineb A. Burnout in the emergency room. Annals of Intensive Care. 2016;6.
143.Malik A, Yamamoto S, Souares A, Sauerborn R. Safe and healthy work environments: An assessment of physicians' level of satisfaction in pakistan. Epidemiology. 2011;22:S273-S274
144.Mirza. AA, Badrek-Amoudi. A. H, Farooq. M. U, Senan. H. A, Aun. R. H, et.al Job satisfaction of surgeons working in hajj pilgrimage: A multicenter study. BMC Proceedings. 2016;10(4).
145.Malach-Pines A. The Burnout Measure, Short Version. International Journal of Stress Management. 2005;12(1):78-88.
146.Malakouti SK, Nojomi M, Salehi M, Bijari B. Job stress and burnout syndrome in a sample of rural health workers, behvarzes, in tehran, iran. Iranian journal of psychiatry. 2011;6(2):70-74.
147.Margalit APA, Glick SM, Benbassat J, Cohen A, Katz M. Promoting a biopsychosocial orientation in family practice: effect of two teaching programs on the knowledge and attitudes of practising primary care physicians. Medical teacher. 2005;27(7):613-618.
148.Martin U, Schinke SP. Organizational and individual factors influencing job satisfaction and burnout of mental health workers. Social Work in Health Care. 1998;28(2):51-62.
149.Masri R, Kadhum M, Farrell SM, Khamees Aa, Al-Taiar H, Molodynski A. Wellbeing and mental health amongst medical students in Jordan: a descriptive study. International review of psychiatry (Abingdon, England). 2019;1-7.
150.Massou A, Doghmi N, Belhaj A, Aboulaala K, et al. Survey on burn out in healthcare providers working in four Moroccan academic intensive care units. Annales Medico-Psychologiques. 2013;171(8):538-542.
151.Maswadi N, Khader YS, Abu Slaih A. Perceived Stress Among Resident Doctors in Jordanian Teaching Hospitals: Cross-Sectional Study. JMIR public health and surveillance. 2019;5(4):e14238.
152.Mazhar SB, Khan A, Bangash S. Job burnout among postgraduate residents- A comparison among different specialities. International Journal of Gynecology and Obstetrics. 2009; 107:S263.
153.McLean CP, a S, Dickstein BD, et al. Posttraumatic growth and posttraumatic stress among military medical personnel. Psychological Trauma: Theory, Research, Practice, and Policy. 2013;5(1):62-68.
154.Mohammed KA-M, Ali EG, Youssef IM, Fahmy MT, Haggag WE-l. Burnout and personality among Egyptian residents. Arab Journal of Psychiatry. 2013;24(2):148-160.
155.Mian I. A. Working with clinicians who work with parents of terrorists. Journal of the American Academy of Child and Adolescent Psychiatry. 2017;56(10):S72-S73
156.Moradi G, Mohraz M, Gouya MM, et al. Problems of providing services to people affected by HIV/AIDS: service providers and recipients perspectives. Eastern Mediterranean health journal = La revue de sante de la Mediterranee orientale = al-Majallah al-sihhiyah li-sharq al-mutawassit. 2015;21(1):20-28.
157.Musa SA, Hamid AA RM. Psychological problems among aid workers operating in Darfur. Social Behavior and Personality: An International Journal. 2008;36(3):407-416.
158.Mustafa OM. Health behaviors and personality in burnout: A third dimension. Medical Education Online. 2015;20.

159. Mustahsan SM. A Resident's Note. Journal of the College of Physicians and Surgeons--Pakistan : JCPSP. 2017;27(10):667.
160. Muzafar Y, Khan HH, Ashraf H, et al. Burnout and its Associated Factors in Medical Students of Lahore, Pakistan. Cureus. 2015;7(11):e390.
161. Nafiaa H, Ouanass A. Frequency of burn-out among Moroccan medical residents. European Psychiatry. 2018;48:S228
162. Naseer S, Raja U, Donia MBL. Effect of Perceived Politics and Perceived Support on Bullying and Emotional Exhaustion: The Moderating Role of Type A Personality. The Journal of psychology. 2016;150(5):606-624.
163. Nematpour S, Behrouzian F, Farashb, i S. Burnout assessment in medical interns and relationship with their coping strategy. Minerva Psichiatrica. 2017;58(2):97-102.
164. Nosratabadi M, Bagheri M, Fazilat MP. The mediation role of resiliency in the relationship of attribution styles and personality dimensions with job burnout in surgeons of Kerman city, Iran. Journal of Kerman University of Medical Sciences. 2015;22(6):690-704.
165. Omar A, Taha A. Beyond clinician burnout predisposing factors, what is the appropriate intervention? Trends in Anaesthesia and Critical Care. 2017;14:19-20.
166. Omar AS, Elmaraghi S, Mahmoud MSA, Khalil MA, Singh R, Ostrowski PJ. Impact of leadership on ICU clinicians' burnout. Vol 412015.
167. Ostrowski P, Omar A. Empowering the intensive care practitioners. Is it a burnout ameliorating intervention? Critical Care. 2016;20.
168. Palareti L, Poti S, Rosu A, Iorio A, Cassis F. When health care professionals express themselves: Results overview of the qualitative phase of Hero initiative. Haemophilia. 2016;22:13.
169. Penix EA, Kim PY, Wilk JE, Adler AB. Secondary traumatic stress in deployed healthcare staff. Psychological trauma : theory, research, practice and policy. 2019;11(1):1-9.
170. Phalkey R, Malik A, Yamamoto S, Manzoor A, Haque A, Sauerborn R. Are doctors safe at work in Pakistan? Tropical Medicine and International Health. 2011;16:349.
171. Protter B, Travin S. The significance of countertransference and related issues in a multiservice court clinic. Bulletin of the American Academy of Psychiatry & the Law. 1983;11(3):223-230.
172. Qidwai W, Beasley JW, Gomez-Clavelina FJ. The present status and future role of family doctors: A perspective from the International Federation of Primary Care Research Networks. Primary Health Care Research and Development. 2008;9(3):172-182.
173. Rad L. Caregiver burnout of Iranian Americans with chronically ill relatives, ProQuest Information & Learning; 2016.
174. Rad M, Shomoossi N, Rakhshani MH, Sabzevari MT. Psychological Capital and Academic Burnout in Students of Clinical Majors in Iran. Acta Facultatis Medicae Naissensis. 2017;34(4):311-319.
175. Rahimnia F, Sharifirad MS. Authentic leadership and employee well-being: The mediating role of attachment insecurity. Journal of Business Ethics. 2015;132(2):363-377.
176. Raja U, Javed Y, Abbas M. A time lagged study of burnout as a mediator in the relationship between workplace bullying and work–family conflict. International Journal of Stress Management. 2018;25(4):377-390.
177. Ravangard R, Dianat S, Shokrpour N. The Factors Affecting Hospital Employees' Turnover Intentions: A Case of Iran. The health care manager. 2019;38(2):166-178.
178. Rostami A, Ghazinour M, Nygren L, Nojumi M, Richter J. Health-related quality of life, marital satisfaction, and social support in medical staff in Iran. Applied Research in Quality of Life. 2013;8(3):385-402.
179. Salar A, Zare Sadegh, Miandoab Nazanin, Shahrakipour M. The survey of the relationship between job burnout and organizational commitment among zahedan medical sciences university staff members in 2016. Indian Journal of Public Health Research and Development. 2016;7(3):283-287.
180. Saban KL, Hogan TP, Defrino D, et al. Burnout and coping strategies of polytrauma team members caring for veterans with traumatic brain injury. Brain injury. 2013;27(3):301-309.
181. Sachs A. Boundary modifications in the treatment of people with dissociative disorders: a pilot study. Journal of trauma & dissociation : the official journal of the International Society for the Study of Dissociation (ISSD). 2013;14(2):159-169.
182. Salam A. The impact of work-related stress on medication errors by health care professionals in Saudi Arabian hospitals, ProQuest Information & Learning; 2017.
183. Santos MM, Qureshi MM, Budohoski KP, et al. The Growth of Neurosurgery in East Africa: Challenges. World neurosurgery. 2018;113:425-435.
184. Sargent P, Millegan J, Delaney E, et al. Health Care Provider Burnout in a United States Military Medical Center During a Period of War. Military medicine. 2016;181(2):136-142.

185. Savel RH, Munro CL. How much care is too much care? Vol 222013.
186. Seddigh R, Abdollahpour E, Azarnik S, Shariati B, Keshavarz-Akhlaghi A-A. Participating in university entrance exam despite repeated failure: a qualitative study of participants' experiences. International journal of medical education. 2016;7:345-353.
187. Sepehrmanesh Z, Ahmadv A, Akasheh G, Saei R. Prevalence of burnout in senior medical students. European Psychiatry. 2010;25.
188. Sevencan F, Cayir E, Uner S. Burnout status of interns and associated factors. Cahiers de sociologie et de demographie medicales. 2010;50(4):501-515.
189. Seyedhosseini J, Zaringhalam M, Farahm S, Mohammadrezaei N, et al. Job satisfaction of emergency medicine residents and specialists in Iran: A cross-sectional study. Trauma Monthly. 2018;23(5):e55963.
190. Shafiq MO, Khaja AF, Alshammari AN, Altayeb MA, Ghabban KM, Khoshhal KI. The journey of orthopaedic surgery from residency to fellowship: A cross-sectional study in the Gulf Cooperation Council countries. Journal of Taibah University Medical Sciences. 2019;14(2):131-138.
191. Shafiullah K, Khan S, Nisar S, Iftikhar-ud-Din, SS. Prevalence of depression among students at Bacha Khan Medical College, Mardan. Journal of Medical Sciences (Peshawar). 2016;24(4):202-205.
192. Shahid M. Psychosocial support programme to reduce stress among emergency physicians. Vol 572007.
193. Shamloo Z, Hashemian, Khoshsima H, Shahverdi A, et al. Validity and reliability of the persian version of the maslach burnout inventory (General Survey Version) in Iranian population. Iranian Journal of Psychiatry and Behavioral Sciences. 2017;11(2):e8168.
194. Shirazy M, Fayed A. Intensivist/patient ratios in closed ICUs in Alexandria, Egypt; an overview. Critical Care. 2016;20.
195. Shorer Y, Rabin S, Zlotnik M, Cohen N, Nadav M, Shiber A. Balint group as a means for Burnout prevention andi mprovement of therapist-patient relationship in general hospital- The Soroka experience.Vol 1552016.
196. Siddique SK, Javed S, Umm-e-Siddiqa. Professional life stress among medical doctors in government and private hospitals. Rawal Medical Journal. 2019;44(1):203-206
197. Siddiqui AA, Jamil M, Kaimkhani GM, et al. Burnout Among Orthopedic Surgeons and Residents in Pakistan. Cureus. 2018;10(8):e3096.
198. Sigsbee B, Bernat JL. Physician burnout: A neurologic crisis. Neurology. 2014;83(24):2302-2306.
199. Simon D, Hallit S, Amine Rayan, Imad J, Soufia M. Burnout syndrome among medical students in a lebanese medical school a questionnaire-based survey. Journal Medical Libanais. 2018;66(5):247-253.
200. Song S. Providers of displaced Syrian adolescents in the Jordanian refugee camps. Journal of the American Academy of Child and Adolescent Psychiatry. 2017;56(10):S72.
201. Stein DJ, Koenen KC, Friedman MJ, et al. Dissociation in posttraumatic stress disorder: Evidence from the World Mental Health Surveys. Biological Psychiatry. 2013;73(4):302-312.
202. Talaei A, Mokhber N, Mohammad-Nejad M, Samari A.A. Burnout and its related factors in staffs of university hospitals in Mashhad in 2006. Koomesh. 2008;9(3):237
203. Talih F, Daher M, Daou D, Ajaltouni J. Examining Burnout, Depression, and Attitudes Regarding Drug Use Among Lebanese Medical Students During the 4 Years of Medical School. Academic psychiatry : the journal of the American Association of Directors of Psychiatric Residency Training and the Association for Academic Psychiatry. 2018;42(2):288-296.
204. Talih F, Warakian R, Ajaltouni J, Shehab AAS, Tamim H. Correlates of Depression and Burnout Among Residents in a Lebanese Academic Medical Center: a Cross-Sectional Study. Academic psychiatry : the journal of the American Association of Directors of Psychiatric Residency Training and the Association for Academic Psychiatry. 2016;40(1):38-45.
205. Taqi AAA. Examination of the effects of personal characteristics and job stress on organizational commitment in the health sector in Kuwait. Journal of the Social Sciences. 2002;30(1):45-68.
206. Tarcan M, Hikmet N, Schooley B, Top M, Tarcan GY. An analysis of the relationship between burnout, socio-demographic and workplace factors and job satisfaction among emergency department health professionals. Applied nursing research : ANR. 2017;34:40-47.
207. Tatli O, Kupcuk O, Vahdati SS, Karaca Y. Assessment of shift programs of emergency department health personnel in four hospitals providing medical training in Turkey and Iran, a brief report. Trauma Monthly. 2018;23(4):e59074.
208. Teixeira R, Martins JC, Pinto SM. Palliative care in critical care units: A systematic literature review. Palliative Medicine. 2018;32(1):58

209. Tyson J. Compassion fatigue in the treatment of combat-related trauma during wartime. Clinical Social Work Journal. 2007;35(3):183-192.
210. Umer A, Naz F. Development and validation of self-report burnout inventory for doctors (SR-BOID). JPMA The Journal of the Pakistan Medical Association. 2016;66(6):717-721.
211. Van den Broeck A, Elst TV, er, et al. Job demands, job resources, burnout, work engagement, and their relationships: An analysis across sectors. Journal of Occupational and Environmental Medicine. 2017;59(4):369-376.
212. Veronese G, Pepe A, ro. Sense of coherence mediates the effect of trauma on the social and emotional functioning of Palestinian health providers. American Journal of Orthopsychiatry. 2014;84(5):597-606.
213. Veronese G, Pepe A, ro. Sense of Coherence as a Determinant of Psychological Well-Being Across Professional Groups of Aid Workers Exposed to War Trauma. Journal of interpersonal violence. 2017;32(13):1899-1920.
214. Voss Horrell SC, Holohan DR, Didion LM, Vance GT. 'Treating traumatized OEF/OIF veterans: How does trauma treament affect the clinician?': Correction to Voss Horrell et al (2011). Professional Psychology: Research and Practice. 2011;42(3):283-283.
215. Vyas-Doorgapersad S, Surujlal J. Alternative therapies for gender-based work wellness: Perceptions of employees. Gender & Behaviour. 2015;13(2):6685-6693.
216. Waheed K, Liaqat N, Ejaz S, et al. Burnout among gynaecological residents in lahore, Pakistan: A cross-sectional survey. Journal of the Pakistan Medical Association. 2017;67(9):1318-1322.
217. Walters TA, Matthews EP, Dailey JI. Burnout in Army health care providers. Military medicine. 2014;179(9):1006-1012.
218. Werneburg BL, Jenkins SM, Friend JL, et al. Improving resiliency in healthcare employees. American Journal of Health Behavior. 2018;42(1):39-50.
219. Whittaker-Howe S, Brown G, Williamson V, Greenberg N. The psychological health of remote area medics in Iraq. Occupational Medicine. 2017;67(9):666-671.
220. Wright ES. Time out for a career break. Professional nurse (London, England). 1994;10(3):198.
221. Yadollahi M, Razmjooei A, Jamali K, Niakan MH, Ghahraman Z. The relationship between professional quality of life (ProQol) and general health in Rajaee trauma hospital staff of Shiraz,Iran. Shiraz E Medical Journal. 2016;17(9):e39253.
222. Yehya A, Sankaranarayanan A, Alkhal A, et al. Job satisfaction and stress among healthcare workers in public hospitals in Qatar. Archives of environmental & occupational health. 2018:1-8.
223. Yesil A, Umit ErgUN, Cumhur A, Fatih E.R, Olun N, Aker A. Validity and reliability of the Turkish version of the professional quality of life scale. Noropsikiyatri Arsivi. 2010;47(2):111-117
224. Younis M, Obaidi AKA, Al-Nuaimi A. The workload of a psychiatric clinic at a general hospital in a post conflict area: A model from Iraq. Journal of Public Mental Health. 2014;13(1):51-58.
225. Yousef DA. Job satisfaction as a mediator of the relationship between job stressors and affective, continuance and normative commitment: A path analytical approach. International Journal of Stress Management. 2002;9(2):99-112.
226. Zadeh ZF, Ahmad KB. Number of working hours and male employees' psychological work-stress levels. Pakistan Journal of Psychological Research. 2008;23(1):29-36.
227. Zafar W, Khan UR, Siddiqui SA, Jamali S, Razzak JA. Workplace Violence and Self-reported Psychological Health: Coping with Post-traumatic Stress, Mental Distress, and Burnout among Physicians Working in the Emergency Departments Compared to Other Specialties in Pakistan. The Journal of emergency medicine. 2016;50(1):167-177.e161.
228. Zamani M, Zamani V. Financial stress during medical residency training: An experience from Iran. Neuropsychiatric Disease and Treatment. 2016;12.

Figure S5: PRISMA Flowchart

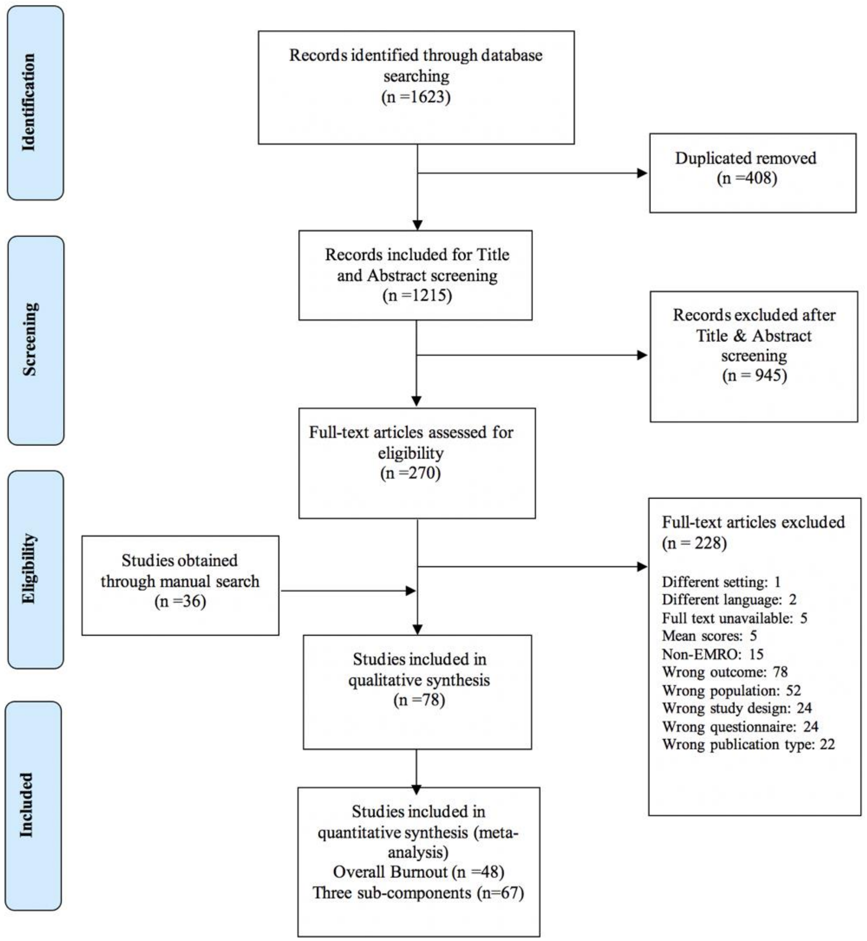

Table S6: Quality assessment of the individual studies

Study No	Included study citation	Was the research question or objective in this paper clearly stated? (1)	Was the study population clearly specified and defined? (2)	Was the participation rate of eligible persons at least 50%? (3)	Were inclusion and exclusion criteria for being in the study prespecified and applied uniformly to all participants? (4)	Was a sample size justification, power description, or variance and effect estimates provided? (5)	Were all the subjects selected or recruited from the same or similar populations (including the same time period)? (6)	Rating
1.	Abbas, Ahmad; 2019[1]	Y	Y	Y	Y	Y	Y	Good
2.	AbdAllah, AM; 2019 [2]	Y	Y	Y	N	Y	Y	Poor
3.	Abdo, SA; 2015 [3]	Y	Y	Y	Y	Y	Y	Good
4.	Abdulaziz, Salman; 2009 [4]	Y	Y	N/A	N	N	Y	Poor
5.	Abdulrahman, Mahera; 2018 [5]	Y	Y	Y	N	N	Y	Poor
6.	Abyad, Abdulrazak; 2016 [6]	Y	Y	Y	N	N	Y	Poor
7.	Agha, Adnan; 2014 [7]	Y	Y	Y	Y	N	Y	Fair
8.	Ahmadpanah, Mohammad; 2015 [8]	Y	Y	N/A	Y	N	Y	Fair
9.	Al-Dabbagh, Abdul Munem; 2019 [9]	Y	Y	Y	Y	N	Y	Fair
10.	A.Al-Subhi, Abdulrahman; 2020 [10]	Y	Y	Y	Y	Y	Y	Good
11.	Al-Dubai,Sami Abdo Radman; 2010[11]	Y	Y	Y	Y	N	Y	Fair
12.	Al-Haddad, Ahmed; 2020 [12]	Y	Y	Y	Y	N	Y	Fair
13.	Al-Hashemi, Tharaya; 2019 [13]	Y	Y	Y	N	Y	Y	Poor
14.	Al-Sareai, N.S; 2013 [14]	Y	Y	Y	N	N	Y	Poor

15.	Al-Youbi, Reem A; 2013 [15]	Y	Y	Y	N	N	Y	Poor
16.	Aldrees, Turki; 2013[16]	Y	Y	Y	Y	Y	Y	Good
17.	Aldrees, Turki; 2015 [17]	Y	Y	Y	Y	N	Y	Fair
18.	Aldress, Turki; 2017 [18]	Y	Y	Y	Y	N	Y	Fair
19.	Alhaffar, Bahaa Aldin; 2019 [19]	Y	Y	Y	Y	N	Y	Fair
20.	Alotaibi, Abdulaziz Khalid; 2019 [20]	Y	Y	Y	N	N	Y	Poor
21.	Alqahtani, Abdulghani M; 2019 [21]	Y	Y	N/A	N	N	Y	Poor
22.	Alsaawi, Abdulmoshen; 2014 [22]	Y	Y	Y	Y	N	Y	Fair
23.	Alsaawi, Abdulmoshen; 2019 [23]	Y	Y	Y	Y	N	Y	Fair
24.	Alsheikh, Khalid A; 2019 [24]	Y	Y	N	N	N	Y	Poor
25.	Alyamani, Alwaleed; 2018 [25]	Y	Y	Y	N	N	Y	Poor
26.	Arvandi, Zeinab; 2016 [26]	Y	Y	N/A	N	N	Y	Poor
27.	Ashkar, Khalil; 2010 [27]	Y	Y	Y	N	N	Y	Poor
28.	Bany Hamdan, Abdullah; 2019 [28]	Y	Y	Y	Y	N	Y	Fair
29.	Belayachi, Jihane; 2016 [29]	Y	Y	N/A	N	N	Y	Poor
30.	Ben Zid, A; 2018 [30]	Y	Y	Y	Y	N	Y	Good
31.	Benbrahim, Zineb; 2019 [31]	Y	Y	N	N	N	Y	Poor
32.	Bin Dahmash, Abdulmajeed; 2019 [32]	Y	Y	Y	N	N	Y	Poor
33.	Chaudhruy,Muhammad Ashraf; 2015 [33]	Y	Y	Y	N	N	Y	Poor
34.	El Kettani, Assiya; 2017 [34]	Y	Y	Y	Y	N	Y	Good

35.	Fauzia, A Khan; 2019 [35]	Y	Y	Y	Y	Y	Y	Good
36.	Farahat, Taghreed Mohamed; 2016 [36]	Y	Y	Y	N	N	Y	Poor
37.	Farahat, Tagraeed Mohamed; 2017 [37]	Y	Y	Y	N	N	Y	Poor
38.	Ghaida M.J, Al-Shoraian; 2011 [38]	Y	Y	Y	N	N	Y	Poor
39.	Hamdan, Motasem; 2017 [39]	Y	Y	Y	Y	N	Y	Fair
40.	Hameed, Tahir Kamal; 2018 [40]	Y	Y	N	Y	N	Y	Fair
41.	Hasan, Husain Isa; 2015 [41]	Y	Y	Y	Y	Y	Y	Good
42.	Hussein, H. Y; 2015 [42]	Y	Y	N/A	N	N	Y	Poor
43.	I, Kacem; 2017 [43]	Y	Y	Y	Y	N	Y	Good
44.	Jadoo, Saad Ahmed Ali; 2018 [44]	Y	Y	Y	N	N	Y	Poor
45.	Jalili, Mohammad; 2013 [45]	Y	Y	Y	N	N	Y	Poor
46.	Jamjoom, Roaa S; 2018 [46]	Y	Y	Y	N	N	Y	Poor
47.	Khalil, Ashkar; 2018 [47]	Y	Y	N	N	N	Y	Poor
48.	Kalani, Simin Dokht; 2017[48]	Y	Y	N	N/A	N/A	Y	Unclear
49.	Khan, Khadija Asim; 2020 [49]	Y	Y	Y	N	N	Y	Poor
50.	Kotb, Amany Ali; 2014 [50]	Y	Y	N/A	N	N	Y	Poor
51.	Liaqat, Naeem; 2019 [51]	Y	Y	N	N	N	Y	Poor
52.	Mohamed, Alorabi; 2019 [52]	Y	Y	Y	N	N	Y	Poor
53.	M, Oumaya; 2009 [53]	Y	Y	Y	N/A	N/A	N/A	Unclear
54.	M, Taei; 2010 [54]	Y	Y	N	N/A	N/A	Y	Unclear
55.	Mohammed,Almhana Ab dulhussein Abdulzahra; 2019 [55]	Y	Y	Y	N/A	N/A	N/A	Unclear
56.	Malik, Awais Amjad; 2016 [56]	Y	Y	Y	N	N	Y	Poor

57.	Marzouki, Hani; 2019 [57]	Y	Y	Y	N	N	Y	Poor
58.	Massou, Salah; 2013 [58]	Y	Y	Y	Y	N	Y	Good
59.	Mazhar, Syeda Batool; 2019 [59]	Y	Y	Y	Y	Y	Y	Good
60.	Mhamdi, Salah; 2018 [60]	Y	Y	Y	Y	N	Y	Good
61.	Moein, Neda; 2018 [61]	Y	Y	N/A	Y	N	Y	Unclear
62.	Mohammed,Khalid Abdul-Moez; 2014 [62]	Y	Y	Y	N	Y	Y	Poor
63.	Mohammed, Sura Bahjat; 2018 [63]	Y	Y	Y	N	Y	Y	Poor
64.	Mortada, Eman M; 2012 [64]	Y	Y	Y	N	Y	Y	Poor
65.	Ok, Abd EL Latief; 2018 [65]	Y	Y	Y	N	Y	Y	Poor
66.	Omr, Amr S; 2019 [66]	Y	Y	Y	N	N	Y	Poor
67.	Osman, Doaa MM; 2019 [67]	Y	Y	Y	N	N	Y	Poor
68.	Sadat-Ali, Mir; 2005 [68]	Y	Y	Y	N	N	Y	Poor
69.	Salem, Mohamed; 2018 [69]	Y	Y	Y	N	N	Y	Poor
70.	Salem, Rana; 2018 [70]	Y	Y	Y	Y	N	Y	Good
71.	Selaihem, Ahmed Abdullah; 2013 [71]	Y	Y	Y	N	N	Y	Poor
72.	Shams, Tarek; 2013 [72]	Y	Y	Y	N	N	Y	Poor
73.	Sheikhmoonesi, Fatemeh; 2017 [73]	Y	Y	Y	N	N	Y	Poor
74.	Sherbiny, El -E; 2017 [74]	Y	Y	Y	N	N	Y	Poor
75.	Soltanifar, Atefeh; 2018 [75]	Y	Y	Y	Y	N	Y	Fair
76.	Yousef, I. M; 2006 [76]	Y	Y	Y	Y	Y	Y	Good
77.	Zarei, Ehsan; 2019 [77]	Y	Y	Y	Y	N	Y	Fair
78.	Zubairi, Akbar Jaleel; 2016 [78]	Y	Y	N	N	N	Y	Poor

Y-Yes N-No N/A- Not applicable

Figure S7: Forest plot showing pooled prevalence across countries

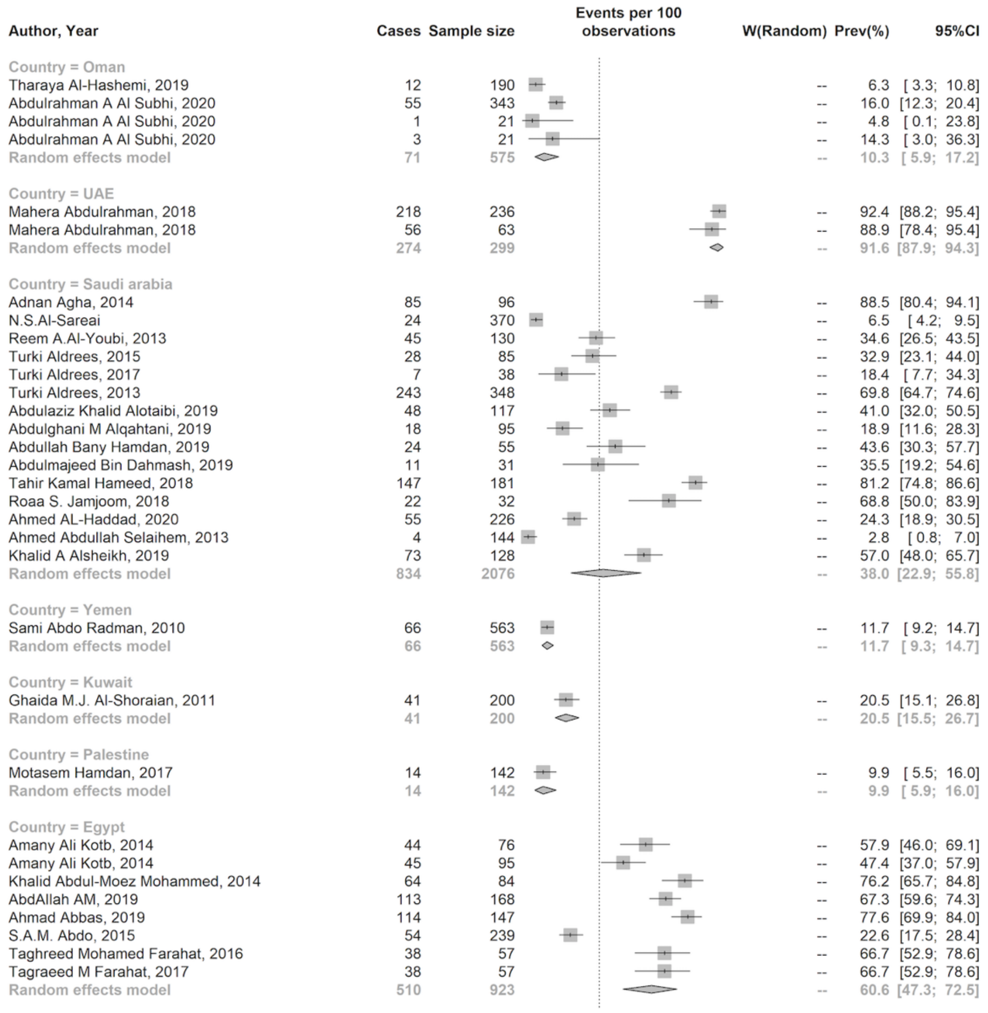

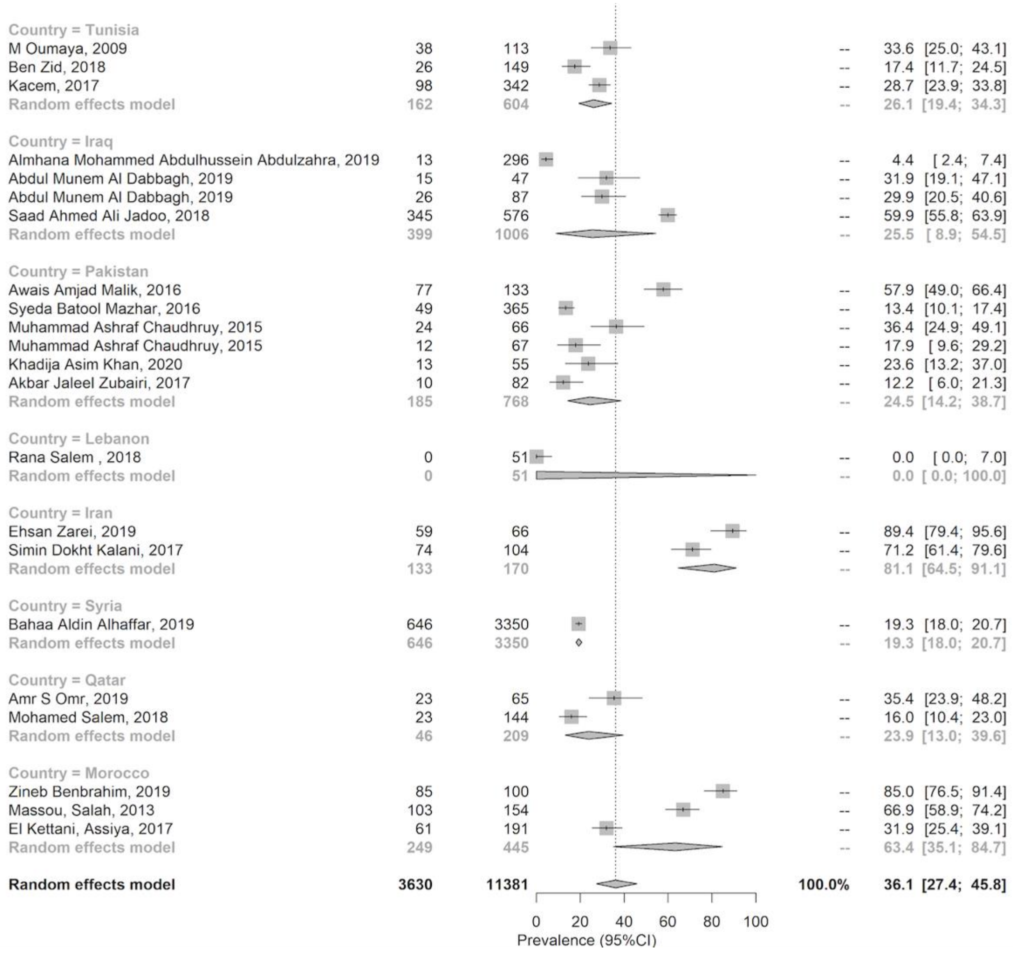
Country = Tunisia
M Oumaya, 2009 38 113 -- 33.6 [25.0; 43.1]
Ben Zid, 2018 26 149 -- 17.4 [11.7; 24.5]
Kacem, 2017 98 342 -- 28.7 [23.9; 33.8]
Random effects model 162 604 -- 26.1 [19.4; 34.3]
Country = Iraq
Almhana Mohammed Abdulhussein Abdulzahra, 2019 13 296 -- 4.4 [2.4; 7.4]
Abdul Munem Al Dabbagh, 2019 15 47 -- 31.9 [19.1; 47.1]
Abdul Munem Al Dabbagh, 2019 26 87 -- 29.9 [20.5; 40.6]
Saad Ahmed Ali Jadoo, 2018 345 576 -- 59.9 [55.8; 63.9]
Random effects model 399 1006 -- 25.5 [8.9; 54.5]
Country = Pakistan
Awais Amjad Malik, 2016 77 133 -- 57.9 [49.0; 66.4]
Syeda Batool Mazhar, 2016 49 365 -- 13.4 [10.1; 17.4]
Muhammad Ashraf Chaudhruy, 2015 24 66 -- 36.4 [24.9; 49.1]
Muhammad Ashraf Chaudhruy, 2015 12 67 -- 17.9 [9.6; 29.2]
Khadija Asim Khan, 2020 13 55 -- 23.6 [13.2; 37.0]
Akbar Jaleel Zubairi, 2017 10 82 -- 12.2 [6.0; 21.3]
Random effects model 185 768 -- 24.5 [14.2; 38.7]
Country = Lebanon
Rana Salem , 2018 0 51 -- 0.0 [0.0; 7.0]
Random effects model 0 51 -- 0.0 [0.0; 100.0]
Country = Iran
Ehsan Zarei, 2019 59 66 -- 89.4 [79.4; 95.6]
Simin Dokht Kalani, 2017 74 104 -- 71.2 [61.4; 79.6]
Random effects model 133 170 -- 81.1 [64.5; 91.1]
Country = Syria
Bahaa Aldin Alhaffar, 2019 646 3350 -- 19.3 [18.0; 20.7]
Random effects model 646 3350 -- 19.3 [18.0; 20.7]
Country = Qatar
Amr S Omr, 2019 23 65 -- 35.4 [23.9; 48.2]
Mohamed Salem, 2018 23 144 -- 16.0 [10.4; 23.0]
Random effects model 46 209 -- 23.9 [13.0; 39.6]
Country = Morocco
Zineb Benbrahim, 2019 85 100 -- 85.0 [76.5; 91.4]
Massou, Salah, 2013 103 154 -- 66.9 [58.9; 74.2]
El Kettani, Assiya, 2017 61 191 -- 31.9 [25.4; 39.1]
Random effects model 249 445 -- 63.4 [35.1; 84.7]
Random effects model 3630 11381 100.0% 36.1 [27.4; 45.8]
0 20 40 60 80 100
Prevalence (95%CI)

Table S8: Age and Gender wise prevalence of Burnout in the Middle East

Included Study Citation	Quality Assessment	Country	Age	Gender	Study Period	Response rate (%)	Category	Specialty/ Subcategory	Instrument cut-offs			Std cut offs used used	OB Definition category	Sample size	OB Prevalence (%)	EE Prevalence (%)	DP Prevalence (%)	PA Prevalence (%)
									EE	DP	PA	Y/N	1*-10*					
AbdAllah, AM; 2019 [2]	Poor	Egypt	.	M and F	1st of Jan 2017 - Apr of 2018.	.	SMP	All (Surgical Specialists and Medical Specialists)	.	.	.	Unclear	1	70	44.25	.	.	.
Al-Dabbagh, Abdul Munem; 2019 [9]	Fair	Iraq	<30 yrs:12 (9.0%) 30-39yrs:64 (47.7%) ≥40yrs:58 (43.3%)	M and F	Apr-June 2017	95.7	GMP	General medical practitioners (Family medical practitioners, General practitioners)	.	.	.	Unclear	NA	134	.	68.66	41.04	26.12
Al-Dabbagh, Abdul Munem; 2019 [9]	Fair	Iraq	.	M and F	Apr-June 2017	95.7	GMP	General medical practitioners (Family medical practitioners, General practitioners)	.	.	.	Unclear	NA	87	29.89	.	.	.
Al-Dabbagh, Abdul Munem; 2019 [9]	Fair	Iraq	.	M and F	Apr-June 2017	95.7	GMP	General medical practitioners (Family medical practitioners, General	.	.	.	Unclear	NA	47	31.91	.	.	.

								practitioners)									
Alsaawi, Abdulmoshen, 2019 [23]	Fair	Saudi Arabia	<25 yrs:2 (0.8%) 25–34 yrs: 161(60.8%) 35–44 yrs:76 (28.7%) 45–55 yrs:20 (7.5%) ≥55 yrs:6 (2.3%)	M and F	2015	60.8	SMP	Surgical Specialists (Emergency medicine)	≥3	≥2	≤4.1	N	NA	265	.	35.85	50.94	40.38
El - Sherbiny E, 2017 [74]	Poor	Saudi Arabia	.	M and F	Jul-Aug 2014	80	SMP	Surgical Specialists (Intensive care Physicians)	>30	>12	<33	N	1	8	12.50	62.50	12.50	37.50
Salem, Rana; 2018 [70]	Good	Lebanon	25-30yrs:9 (17.6%) 30-40 yrs:14 (27.5%) 40-60 yrs:22 (43.1%) >60:6 (11.8%)	M and F	.	73	SMP	Medical specialists (Oncology)	>27	>13	<30	N	1	51	0.00	33.33	13.73	68.63

A Al Subhi, Abdulrahman; 2020 [10]	Good	Oman	Mean:29 yrs	M and F	Apr 2018 to Apr 2019	100	SMP	Medical Specialists (Internal Medicine, Community medicine), OBGYN	>27	>13	<31	N	2	343	16.03	17.78	42.57	40.82
Abbas, Ahmad; 2019 [1]	Good	Egypt	Mean:29.47±5.49 years range:17–49 years	M and F	1 April - 30 Oct 2017	.	SMP	Surgical Specialists (Intensive care Physicians)	.	.	.	Unclear	1	147	77.55	38.78	70.07	51.02
AbdAllah, AM; 2019 [2]	Poor	Egypt	Range:25-29 Mean± SD: 27.46 (0.82).	M and F	1st of Jan 2017 - Apr of 2018.	.	SMP	All (Surgical Specialists and Medical Specialists)	.	.	.	Unclear	1	168	67.26	52.98	64.29	75.00
AbdAllah, AM; 2019 [2]	Poor	Egypt	.	M	1st of Jan 2017 - Apr of 2018.	.	SMP	All (Surgical Specialists and Medical Specialists)	.	.	.	Unclear	1	98	83.72	.	.	.
AbdAllah, AM; 2019 [2]	Poor	Egypt	.	F	1st of Jan 2017 - Apr of 2018.	.	SMP	All (Surgical Specialists and Medical Specialists)	.	.	.	Unclear	1	70	44.3	.	.	.
Abdo, SA; 2015 [3]	Good	Egypt	Mean:31.0 (6.0) yrs	M and F	01 Nov-30 Apr 2013	89.8	SMP	All	>27	>13	<31	N	3	239	22.59	39.75	22.59	99.16

Abdulaziz, Salman; 2009 [4]	Poor	Saudi Arabia	21-25 yrs: 19 (9.27%)<br>26-30 yrs: 162 (79.02%)<br>31-35 yrs: 21 (10.24%)<br>36 yrs +: 3 (1.46%)	M and F	May 2005	.	All	All	≥27	≥13	<31	N	NA	205	.	54.15	23.90	69.76
Abdulrahman, Mahera; 2018 [5]	Poor	UAE	.	M and F	May and Dec 2016	68	All	All	≥ 27	≥ 10	.	Y	Unclear	286	.	75.52	83.56	74.23
Abdulrahman, Mahera; 2018 [5]	Poor	UAE	.	F	May and Dec 2016	68	All	All	≥ 27	≥ 10	.	Y	Unclear	236	92.37	.	.	.
Abdulrahman, Mahera; 2018 [5]	Poor	UAE	.	M	May and Dec 2016	68	All	All	≥ 27	≥ 10	.	Y	Unclear	63	88.89	.	.	.
Abyad, Abdulrazak; 2016 [6]	Poor	Middle East (Iran, Saudi Arabia, Kuwait, Lebanon, Iraq,)	Mean: 47.4 years	M and F	.	71	GMP	General medical practitioners (Family medical practitioners)	>27	>10	<33	N	NA	500	.	44.00	30.00	28.80
Agha, Adnan; 2014 [7]	Fair	Saudi Arabia	<29 yrs:21<br>30-39 yrs:48<br>40-50 yrs:27	M and F	Aug - Oct 2012	NA	SMP	All	.	.	.	Y	2	96	88.54	68.75	63.54	38.54

Ahmadpanah, Mohammad; 2015 [8]	Fair	Iran	Range:26-49 yrs<br>Mean :32±5.06	M and F	Jan-Dec 2011	NA	GMP	General Practitioners	Unclear	Unclear	Unclear	Unclear	Unclear	100	.	15.00	15.00	10.00
Ahmed AL-Haddad, 2020 [12]	Fair	Saudi Arabia	25 <35 yrs:128<br>35 <45 yrs:59<br>45 <50 yrs:26<br>%0 and above :13	M and F	Mar 2018-Aug 2019	80.7	All	All	.	.	.	Unclear	1	226	24.34	47.35	51.33	40.27
Al-Dubai, Sami Abdo Radman; 2010 [11]	Fair	Yemen	Mean: 33.3 ±5.7 yrs<br>Range: 25-55 yrs	M and F	Dec 2006-July 2007	70.4	Medical doctors undefined	All	≥ 27	≥ 13	≤ 31	N	1	563	11.72	63.23	19.36	41.56
Al-Hashemi, Tharaya; 2019 [13]	Poor	Oman	(18–29):36 (18.9%)<br>(30–39):95 (50.0%)<br>(40–49):48 (25.3%)<br>≥50: 11 (6.3%)	M and F	Mar-May 2017	96	GMP	General medical practitioners (Primary health care physicians)	≥ 26	≥ 9	≤ 33	N	1	190	6.32	17.89	38.42	21.58
Al-Sareai, N.S; 2013 [14]	Poor	Saudi Arabia	26-60 yrs<br>Mean:39.8 yrs	M and F	Oct 2010-Jun 2011	94.9	GMP	All	≥ 26	≥ 9	≤ 33	N	1	370	6.49	29.46	15.68	19.73
Al-Sareai, N.S; 2013 [14]	Poor	Saudi Arabia	.	M	Oct 2010-Jun 2011	94.9	GMP	All	≥ 26	≥ 9	≤ 33	N	1	303	.	31.02	16.50	19.80

Al-Sareai, N.S; 2013 [14]	Poor	Saudi Arabia	.	F	Oct 2010-Jun 2011	94.9	GMP	All	≥ 26	≥ 9	≤ 33	N	1	67	.	22.39	11.94	19.40
Al-Youbi, Reem A; 2013 [15]	Poor	Saudi Arabia	Range:25-45 yrs Mean 30 ± 5yrs	M and F	3 consecutive months in 2010	65	SMP	General Paediatricians	.	.	.	Unclear	1	130	34.62	.	.	.
Al-Youbi, Reem A; 2013 [15]	Poor	Saudi Arabia	.	M	3 consecutive months in 2010	65	SMP	General Paediatricians	.	.	.	Unclear	1	58	39.66	.	.	.
Al-Youbi, Reem A; 2013 [15]	Poor	Saudi Arabia	.	F	3 consecutive months in 2010	65	SMP	General Paediatricians	.	.	.	Unclear	1	72	30.56	.	.	.
Aldrees, Turki; 2013 [16]	Good	Saudi Arabia	Mean:35 yrs	M and F	Oct and Nov 2010	74	All	All	≥27	≥13	<31	N	2	348	69.83	54.02	34.77	33.33
Aldrees, Turki; 2013 [16]	Good	Saudi Arabia	.	F	Oct and Nov 2010	74	All	All	.	.	.	N	2	96	79.17	.	.	.
Aldrees, Turki; 2013 [16]	Good	Saudi Arabia	.	M	Oct and Nov 2010	74	All	All	.	.	.	N	2	252	66.27	.	.	.
Aldrees, Turki; 2015 [17]	Fair	Saudi Arabia	Mean:29 yrs Range:25-40 yrs	M and F	Mar-May 2013	69	SMP	Medical Specialists (Oto-rhino-laryngology)	≥ 27	≥ 10	≥ 40	N	7	85	44.71	62.35	55.29	16.47

Aldrees, Turki; 2015 [17]	Fair	Saudi Arabia	Mean:29 yrs Range:25-40 yrs	M and F	Mar-May 2013	69	SMP	Medical Specialists (Oto-rhino-laryngology)	≥ 27	≥ 10	≥ 40	N	1	85	32.94	62.35	55.29	16.47
Aldrees, Turki; 2017 [18]	Fair	Saudi Arabia	28 ±1.9 yrs Range:25-32	M and F	Apr 2015	60	SMP	Surgical Specialists (Plastic Surgery)	≥ 27	≥ 13	≤ 31	N	7	38	47.37	71.05	50.00	28.95
Aldrees, Turki; 2017 [18]	Fair	Saudi Arabia	28 ±1.9 yrs Range:25-32	M and F	Apr 2015	60	SMP	Surgical Specialists (Plastic Surgery)	≥ 27	≥ 13	≤ 31	N	1	38	18.42	71.05	50.00	28.95
Alhaffar, Bahaa Aldin; 2019 [19]	Fair	Syria	21–25 yrs:1139 (34%) 26–30 yrs: 1994 (59.50%) 31–35 yrs: 217 (6.50%)	M and F	.	87.3	GMP	General medical practitioners (General practitioners)	>27	>9	<33	N	1	3350	19.28	77.88	54.60	13.67
Alhaffar, Bahaa Aldin; 2019 [19]	Fair	Syria	.	F	.	87.3	GMP	General medical practitioners (General practitioners)	>27	>9	<33	N	1	1477	.	71.63	54.03	12.12
Alhaffar, Bahaa Aldin; 2019 [19]	Fair	Syria	.	M	.	87.3	GMP	General medical practitioners (General practitioners)	>27	>9	<33	N	1	1873	.	82.81	54.99	14.90

Alotaibi, Abdulaziz Khalid; 2019 [20]	Poor	Saudi Arabia	.	M and F	Jan 2018	70	SMP	Surgical Specialists (Ophthalmology)	≥27	≥ 13	≤31	N	2	117	41.03	37.61	17.09	31.62
Alqahtani, Abdulghani M; 2019 [21]	Poor	Saudi Arabia	.	.	NA	NA	SMP	Surgical Specialists (Emergency medicine)	≥26	≥ 9	≤33	N	1	95	18.95	81.05	24.21	27.37
Alsaawi, Abdulmoshen, 2014 [22]	Fair	Saudi Arabia	35-44yrs:30 (56%)	M and F	.	74	SMP	Surgical Specialists (Emergency medicine)	≥27	≥10	≤33	Y	NA	53	.	39.62	39.62	32.08
Alsheikh, Khalid A; 2019 [24]	Poor	Saudi Arabia	Mean:28.4 (±2.5) years	M and F	Sept-Oct 2018	47.2	SMP	Surgical Speciality (Orthopaedics)	≥27	≥13	≤31	N	NA	142	.	50.00	39.44	39.44
Alsheikh, Khalid A; 2019 [24]	Poor	Saudi Arabia	.	M	Sept-Oct 2018	47.2	SMP	Surgical Speciality (Orthopaedics)	≥27	≥13	≤31	N	NA	128	57.03	.	.	.
Alsheikh, Khalid A; 2019 [24]	Poor	Saudi Arabia	.	F	Sept-Oct 2018	47.2	SMP	Surgical Speciality (Orthopaedics)	≥27	≥13	≤31	N	NA	14	50.00	.	.	.
Alyamani, Alwaleed; 2018 [25]	Poor	Saudi Arabia	Mean:28 yrs	M and F	15 May 2018 and 31 May 2018	66.67	SMP	All	>29	>11	<33	N	NA	200	.	12.50	51.00	31.50
Arvandi, Zeinab; 2016 [26]	Poor	Iran	46.7±6.7 yrs	M and F	NA	NA	SMP	All	.	.	.	NA	NA	143	.	28.67	5.59	36.36

Bany Hamdan, Abdullah, 2019 [28]	Fair	Saudi Arabia	.	M and F	June 2016-June 2017	62.8	SMP	Medical specialists (Oncology)	.	.	.	Unclear	Unclear	55	44.00	.	.	.
Belayachi, Jihane; 2016 [29]	Poor	Morocco	Mean:29.3 ±3.2	M and F	April-Oct 2010	NA	All	All	.	.	.	Unclear	Unclear	198	.	83.84	16.67	13.13
Ben Zid, A; 2018 [30]	Good	Tunisia	Mean (SD):29 yrs (2.36) 25-40 yrs	M and F	<2017	76.8	SMP	All	.	.	.	.	1	149	17.45	.	.	.
Benbrahim, Zineb; 2019 [31]	Poor	Morocco	.	M and F	Jan 2018	25	SMP	Medical Specialists (Oncology)	.	.	.	Unclear	1	100	85.00	57.00	44.00	20.00
Benbrahim, Zineb; 2019 [31]	Poor	Morocco	.	M	Jan 2018	25	SMP	Medical Specialists (Oncology)	.	.	.	Unclear	1	25	88.00	.	.	.
Benbrahim, Zineb; 2019 [31]	Poor	Morocco	.	F	Jan 2018	25	SMP	Medical Specialists (Oncology)	.	.	.	Unclear	1	75	84.00	.	.	.
Bin Dahmash, Abdulmajeed; 2019 [32]	Poor	Saudi Arabia	25-27 yrs:12 (41.4%) 28-30 yrs:12 (41.4%) 31-33 yrs:5 (17.2%)	M and F	Mar 2018	83.8	SMP	Surgical Specialists (Plastic Surgery)	>26	>12	<32	N	7	31	35.48	67.74	38.71	54.84
Chaudhruy, Muhammad Ashraf; 2015 [33]	.	Pakistan	Mean:35.10 ± 11.16.	M and F	Mar-Sept 2014.	.	GMP	General medical practitioners (General practitioners)	≥27	≥13	≥39	N	Unclear	133	.	21.80	16.54	25.56

Chaudhru y, Muhamm ad Ashraf; 2015 [33]	Poor	Pakist an	.	M	Mar - Sept 2014.	.	GMP	General medical practitioner s (General practitioner s)	≥27	≥13	≥40	N	1	66	36.36	.	.	.
Chaudhru y, Muhamm ad Ashraf; 2015	Poor	Pakist an	.	F	Mar - Sept 2014.	.	GMP	General medical practitioner s (General practitioner s)	≥27	≥13	≥41	N	1	67	17.91	.	.	.
Doaa MM Osman, 2019 [67]	Poor	Egypt	.	M and F	May - July 2017	86*	GMP	General medical practitioner s (Medical Doctors)	≥27	≥13	≤21	N	NA	134	.	59.70	38.81	10.45
El Kettani, Assiya, 2017 [34]	Good	Moro cco	Mean:26.7 yrs	M and F	2013-2014	63.7	SMP	All	>29	>11	<34	N	1	191	31.94	.	.	.
Farahat, Taghreed Mohamed ; 2016 [36]	Poor	Egypt	Mean:33.7 yrs	F	Aug 2014 - May of 2016	91.6	All	All	.		.	Uncl ear	Uncle ar	40	51.20	.	.	.
Farahat, Taghreed Mohamed ; 2016 [36]	Poor	Egypt	Mean:33.7 yrs	M	Aug 2014 - May of 2017	91.6	All	All	.		.	Uncl ear	Uncle ar	36	48.80	.	.	.
Farahat, Tagraeed Mohamed ; 2017 [37]	Poor	Egypt	.	F	Aug 2014 - May of 2019	91.6	All	All	>27	>13	<31	N	Uncle ar	40	.	77.50	77.50	47.50
Farahat, Tagraeed Mohamed ; 2017 [37]	Poor	Egypt	.	M	Aug 2014 - May of 2020	91.6	All	All	>27	>13	<31	N	Uncle ar	36	.	69.44	77.78	41.67

Fauzia A Khan, 2019 [35]	Good	Pakistan	.	M and F	2017	74.5	SMP	Surgical specialists (Anaesthesiology)	>30	>12	≤ 33	N	NA	447	.	39.37	68.46	50.34
Fauzia A Khan, 2019 [35]	Good	Pakistan	.	M	2017	74.5	SMP	Surgical specialists (Anaesthesiology)	>30	>12	≤ 33	N	NA	299	.	41.14	70.23	49.83
Fauzia A Khan, 2019 [35]	Good	Pakistan	.	F	2017	74.5	SMP	Surgical specialists (Anaesthesiology)	>30	>12	≤ 33	N	NA	148	.	35.81	64.86	51.35
Ghaida M.J. Al-Shoraian, 2011[38]	Poor	Kuwait	.	M and F	Aug 2010-Mar 2011	52.90	GMP	General medical practitioners (Family medical practitioners, Primary health care physicians)	≥27	≥10	≤33	Y	1	200	20.50	.	.	.
Ghaida M.J. Al-Shoraian, 2011 [38]	Poor	Kuwait	.	M and F	Aug 2010-Mar 2011	52.90	GMP	General medical practitioners (Family medical practitioners, Primary health care physicians)	≥27	≥10	≤33	Y	2	200	26.50	.	.	.

Ghaida M.J. Al-Shoraian, 2011[38]	Poor	Kuwait	.	M and F	Aug 2010-Mar 2011	52.90	GMP	General medical practitioners (Family medical practitioners, Primary health care physicians)	≥27	≥10	≤33	Y	10	200	22.00	.	.	.
Ghaida M.J. Al-Shoraian, 2011 [38]	Poor	Kuwait	<40 yrs:37 ≥ 40yrs:58	M and F	Aug 2010-Mar 2011	52.90	GMP	General medical practitioners (Primary health care physicians)	≥27	≥10	≤33	Y	1	95	36.84	63.16	65.26	61.05
Ghaida M.J. Al-Shoraian, 2011 [38]	Poor	Kuwait	<40 yrs:77 ≥ 40yrs:28	M and F	Aug 2010-Mar 2011	52.90	GMP	General medical practitioners (Family medical practitioners)	≥27	≥10	≤33	Y	1	105	5.71	19.05	27.62	33.33
Hamdan, Motasem; 2017 [39]	Fair	Palestine	.	M and F	Jul-Sept 2013	74.5	SMP	Surgical Specialists (Emergency medicine)	≥27	≥10	≤33	Y	1	142	9.86	71.83	31.69	31.69

Hameed, Tahir Kamal; 2018 [40]	Fair	Saudi Arabia	.	M and F	2013-2014	43	SMP	All (Surgical Specialists and Medical Specilaists)	≥27	≥ 10	.	Unclear	4	181	81.22	62.43	70.72	66.85
Hameed, Tahir Kamal; 2018 [40]	Fair	Saudi Arabia	.	M	2013-2014	43	SMP	All (Surgical Specialists and Medical Specilaists)	≥27	≥ 10	.	Unclear	4	75	73.33	.	.	.
Hameed, Tahir Kamal; 2018 [40]	Fair	Saudi Arabia	.	F	2013-2014	43	SMP	All (Surgical Specialists and Medical Specilaists)	≥27	≥ 10	.	Unclear	4	106	85.85	.	.	.
Hasan, Husain Isa; 2015 [41]	2015	Bahrain	Mean ±SD :30-40 ±9	M and F	.	87.7	SMP	All	.	.	.	Unclear	NA	202	.	43.07	26.73	22.28
Hasan, Husain Isa; 2015 [41]	2015	Bahrain	.	M	.	87.7	SMP	All	.	.	.	Unclear	NA	122	.	39.34	28.69	54.10
Hasan, Husain Isa; 2015 [41]	2015	Bahrain	.	F	.	87.7	SMP	All	.	.	.	Unclear	NA	79	.	49.37	24.05	48.10
Hussein, H. Y; 2015 [42]	Poor	UAE	<30 yrs:5 (4.9%) 30-40 yrs:38 (37.3%) >40 yrs:59 (57.8%)	M and F	.	.	GMP	General Medical Practitioners (Family medical practitioners)	.	.	.	Unclear	NA	102	.	42.16	13.73	22.55

I, Kacem; 2017[43]	Good	Tunisia	23-25:77 (22.5%) 26-30:245 (71.6%) >30:20 (5.8%)	M and F	.	52.7	GMP	Medical Interns	>29	>11	<34	N	1	342	28.65	.	.	.
I, Kacem; 2017 [43]	2017	Tunisia	.	M	.	52.7	GMP	Medical Interns	>29	>11	<34	N	1	162	30.25	.	.	.
I, Kacem; 2017 [43]	2017	Tunisia	.	F	.	52.7	GMP	Medical Interns	>29	>11	<34	N	1	180	27.22	.	.	.
Jadoo, Saad Ahmed Ali; 2018 [44]	Poor	Iraq	Mean ±SD: 40.43 ±8.59 Range:27-56 yrs ≥40 yrs:291 (50.5%) < 40 yrs:285 (49.5%)	M and F	Jan-Jun 2014	87.3	GMP	General medical practitioners (Medical Doctors)	≥27	.	.	Unclear	8	576	59.90	.	.	.
Jalili, Mohammad; 2013 [45]	Poor	Iran	Mean:33.6 yrs	M and F	Mar-Apr 2008	88	SMP	Surgical Specialists (Emergency medicine)	>27	>10	<33	Y	NA	158	.	36.71	38.75	76.13
Jamjoom, Roaa S; 2018 [46]	Poor	Saudi arabia	NA	M and F	May 2016	64%	SMP	General Paediatricians	>27	≥10	≤ 33	Y	1	32	68.75	43.75	71.88	40.63
Kalani, Simin Dokht; 2017 [48]	Unclear*	Iran	.	M and F	.	.	SMP	All	.	.	.	Unclear	Unclear	104	71.15	.	.	.
Khalil Ashkar, 2018 [47]	Poor	Lebanon	<40 yrs:51 (33.6%)	M and F	01 Aug-20 Sept 2013	30.20	SMP	All (Surgical Specialists and	≥27	>9	<33	N	NA	156	.	37.18	12.82	14.10

			≥40 yrs:101 (37.1%0					Medical Specilaists)										
Khalil Ashkar, 2018 [47]	Poor	Leba non	.	M	01 Aug-20 Sept 2013	30.20	SMP	All (Surgical Specialists and Medical Specilaists)	≥27	>9	<33	N	NA	95	.	33.68	14.74	15.79
Khalil Ashkar, 2018 [47]	Poor	Leba non	.	F	01 Aug-20 Sept 2013	30.20	SMP	All (Surgical Specialists and Medical Specilaists)	≥27	>9	<33	N	NA	56	.	44.64	10.71	10.71
Khalil, Ashkar; 2010 [27]	Poor	Leba non	24-30 yrs: 141 (91%) >30 yrs:14 (9%)	M and F	20 May - 20 Jun 2008	75	All	All	>27	>9	<33	N	NA	155	.	67.74	47.10	37.42
Khalil, Ashkar; 2010 [27]	Poor	Leba non	.	M	20 May - 20 Jun 2008	75	All	All	>27	>9	<33	N	NA	86	.	61.63	44.19	30.23
Khalil, Ashkar; 2010 [27]	Poor	Leba non	.	F	20 May - 20 Jun 2008	75	All	All	>27	>9	<33	N	NA	69	.	75.36	50.72	46.38
Khan, Khadija Asim; 2020 [49]	Poor	Pakist an	Mean:44.8 9 ± 8.50 Range:32-56 yrs	M and F	Jan-Jun 2017	.	SMP	OBGYN	≥27	≥ 10	≤33	Y	10	55	23.64	43.64	41.82	16.36
Kotb, Amany Ali; 2014 [50]	Poor	Egypt	.	M	.	.	All	All	≥27	≥13	≤ 31	N	6	76	57.89	.	.	.
Kotb, Amany Ali; 2014 [50]	Poor	Egypt	.	F	.	.	All	All	≥27	≥13	≤ 31	N	6	95	47.37	.	.	.

Liaqat, Naeem; 2019 [51]	Poor	Pakistan	Mean:44.89 ± 8.50 Range:31-58 yrs	M and F	Jan - Feb, 2017	36.6	SMP	Surgical Specialists (other types of surgery)	.	.	.	Unclear	NA	40	.	17.50	12.50	40.00
Liaqat, Naeem; 2019 [51]	Poor	Pakistan	.	M	Jan-Feb 2017	36.6	SMP	Surgical Specialists (other types of surgery)	.	.	.	Unclear	NA	34	.	17.65	14.71	35.29
Liaqat, Naeem; 2019 [51]	Poor	Pakistan	.	F	Jan-Feb 2017	36.6	SMP	Surgical Specialists (other types of surgery)	.	.	.	Unclear	NA	6	.	16.67	0.00	66.67
M Oumaya, 2009 [53]	Unclear*	Tunisia	.	M and F	.	85.6	GMP	General medical practitioners (Primary health care physicians)	.	.	.	Unclear	Unclear	113	33.63	35.40	21.24	39.82
M, Taei, 2010 [54]	Unclear*	Iran	.	M and F	2009	.	GMP	General Practitioners	.	.	.	Unclear	Unclear	150	.	27.33	94.67	74.00
Malik, Awais Amjad; 2016 [56]	Poor	Pakistan	.	M and F	.	66.5	SMP	Surgical Specialists	.	.	.	Unclear	10	133	57.89	50.38	49.62	53.38
Malik, Awais Amjad; 2016 [56]	Poor	Pakistan	.	M	.	66.5	SMP	Surgical Specialists	.	.	.	Unclear	10	98	48.98	.	.	.
Malik, Awais Amjad; 2016 [56]	Poor	Pakistan	.	F	.	66.5	SMP	Surgical Specialists	.	.	.	Unclear	10	35	34.29	.	.	.

Marzouki, Hani; 2019 [57]	Poor	Saudi Arabia	Mean:43.8 (±9.2)	M and F	Dec 2016 - Jun 2017	77	GMP	General medical practitioners (General practitioners)	>30	>12	<33	N	NA	77	.	9.09	23.38	42.86
Massou, Salah, 2013 [58]	Good	Morocco	.	M and F	.	.	SMP	Surgical specialists	>29	>11	<34	N	Unclear	154	66.88	48.05	20.78	42.86
Mazhar, Syeda Batool; 2019 [59]	Good	Pakistan	Mean:28.68 ±4.58 yrs 20-29:244, 66.9% 30-39:105, 28.8% >40: 14, 3.8	M and F	01 Oct 2016-31 Jan 2017	100	SMP	All	>27	>13	<31	N	1	365	13.42	38.36	27.40	56.99
Mazhar, Syeda Batool; 2019 [59]	Good	Pakistan	.	F	01 Oct 2016-31 Jan 2017	100	SMP	All	>27	>13	<31	N	1	238	.	41.18	.	.
Mazhar, Syeda Batool; 2019 [59]	Good	Pakistan	.	M	01 Oct 2016-31 Jan 2017	100	SMP	All	>27	>13	<31	N	1	127	.	33.07	.	.
Mhamdi, Salah, 2018 [60]	Good	Tunisia	Mean:40.83 yrs	M and F	7/6/05	72.19	SMP	Surgical Specialists (Anaesthesiology)	>29	>11	<34	N	NA	35	.	20.00	17.14	31.43
Moein, Neda; 2018 [61]	Unclear*	Iran	Mean:35.3 ±8.5 years	M and F	2017-2018	.	GMP	General Medical Practitioners (Family medical practitioners)	≥27	≥13	≥31	N	NA	NA	.	22.58	3.23	28.39

Mohamed, Alorabi; 2019 [52]	Poor	Egypt	<40yrs:33 (70.2%) >40 yrs:14 (29.8%)	M and F	.	90	SMP	Medical specialists (Oncology)	.	.	.	Unclear	NA	47	.	72.34	48.94	38.30
Mohammed, Almhana Abdulhussein Abdulzahra; 2019 [55]	Unclear*	Iraq	.	M and F	.	84	GMP	General medical practitioners (Medical Doctors)	.	.	.	Unclear	Unclear	296	4.39	52.70	14.19	26.01
Mohammed, Khalid Abdul-Moez; 2014 [62]	Poor	Egypt	.	M	2012	64.6	GMP	General medical practitioners (General practitioners)	.	.	.	Unclear	10	84	76.19	80.95	64.29	52.38
Mohammed, Sura Bahjat; 2018 [63]	Poor	Iraq	Mean:34.75 ± 7.5	M and F	2016	92	GMP	General medical practitioners (General practitioners)	≥27	≥10	≤16	N	NA	.	.	0	78.71	17.42
Mortada, Eman M; 2012 [64]	Poor	Egypt	Mean:36.9 ± 8.9 years	M and F	Jan - April 2012	.	SMP	All	≥27	≥ 10	≤33	Y	NA	250	.	37.6	27.60	17.20
Ok, Abd EL Latief, 2018 [65]	Poor	Egypt	Range:20 - 58 yrs. Mean: 39.45± 10.30	M and F	Oct-Dec 2016	.	SMP	All (Surgical Specialists and Medical Specilaists)	≥30	≥12	≤33	N	NA	134	.	55.97	58.21	23.13
Omr, Amr S; 2019 [66]	Poor	Qatar	.	M and F	.	.	SMP	Surgical Specialists (Intensive care Physicians)	>27	.	.	N	8	65	35.38	35.38		

Sadat-Ali, Mir; 2005 [68]	Poor	Saudi Arabia	Avg:45.72 ±6.82 Range:33-57 yrs	M and F	Sept 2003 - Oct 2004	67.6	SMP	Surgical Speciality (Orthopaedics)	.	.	.	Unclear	NA	102	.	34.31	40.20	11.76
Salem, Mohamed; 2018 [69]	Poor	Qatar	<40 yrs:39 (31.45%) ≥40 yrs:85 (68.55%)	M and F	.	90	GMP	General Medical Practitioners (Family medical practitioners)	≥27	≥13	≤31	N	10	144	15.97	11.81	29.17	58.33
Salem, Mohamed; 2018 [69]	Poor	Qatar	.	M	.	90	GMP	General Medical Practitioners (Family medical practitioners)	≥27	≥13	≤31	N	10	90	14.44	.	.	.
Salem, Mohamed; 2018 [69]	Poor	Qatar	.	F	.	90	GMP	General Medical Practitioners (Family medical practitioners)	≥27	≥13	≤31	N	10	49	20.41	.	.	.
Selaihem, Ahmed Abdullah; 2013 [71]	Poor	Saudi Arabia	Mean ±SD:37.08 ± 8.48 Range:25 to 62 years	M and F	Apr 2010.	72	GMP	General medical practitioners (Primary health care physicians)	>26	>9	<34	N	10	144	39.58	.	.	.
Selaihem, Ahmed Abdullah; 2013 [71]	Poor	Saudi Arabia	Mean ±SD:37.08 ± 8.48 Range:25 to 62 years	M and F	Apr 2010.	72	GMP	General medical practitioners (Primary health care physicians)	>26	>9	<34	N	1	144	2.78	.	.	.

Selaihem, Ahmed Abdullah; 2013 [71]	Poor	Saudi Arabia	Mean ±SD:37.08 ± 8.48 Range:25 to 62 years	M and F	Apr 2010.	72	GMP	General medical practitioners (Primary health care physicians)	>26	>9	<34	N	2	144	33.33	.	.	.
Selaihem, Ahmed Abdullah; 2013 [71]	Poor	Saudi Arabia	Mean ±SD:37.08 ± 8.48 Range:25 to 62 years	M and F	Apr 2010.	72	GMP	General medical practitioners (Primary health care physicians)	>26	>9	<34	N	NA	144	.	53.47	38.89	44.44
Selaihem, Ahmed Abdullah; 2013 [71]	Poor	Saudi Arabia	.	M	Apr 2010.	72	GMP	General medical practitioners (Primary health care physicians)	>26	>9	<34	N	NA	88	.	51.14	40.91	25.00
Selaihem, Ahmed Abdullah; 2013 [71]	Poor	Saudi Arabia	.	F	Apr 2010.	72	GMP	General medical practitioners (Primary health care physicians)	>26	>9	<34	N	NA	56	.	57.14	35.71	33.93
Shams, Tarek; 2013 [72]	Poor	Egypt	.	M and F	Mar-June 2011	73	SMP	Surgical specialists (Anaesthesiology)	.	.	.	Unclear	NA	134	.	45.52	41.04	42.54
Shams, Tarek; 2013 [72]	Poor	Egypt	.	M	Mar-June 2011	73	SMP	Surgical specialists (Anaesthesiology)	.	.	.	Unclear	NA	99	.	46.46	41.41	42.42
Shams, Tarek; 2013 [72]	Poor	Egypt	.	F	Mar-June 2011	73	SMP	Surgical specialists (Anaesthesiology)	.	.	.	Unclear	NA	35	.	42.86	40.00	42.86

Sheikhmoonesi, Fatemeh; 2017 [73]	Poor	Iran	.	M and F	2012	60	SMP	All	≥27	≥10	≤33	Y	1	106	.	4.72	16.04	28.30
Sheikhmoonesi, Fatemeh; 2017 [73]	Poor	Iran	.	M	2012	60	SMP	All	≥27	≥10	≤33	Y	NA	54	.	3.70	16.67	38.89
Sheikhmoonesi, Fatemeh; 2017 [73]	Poor	Iran	.	F	2012	60	SMP	All	≥27	≥10	≤33	Y	NA	52	.	5.77	15.38	55.77
Soltanifar, Atefeh; 2018 [75]	Fair	Iran	30-48 yrs Median:36 yrs	F	Dec 2016 - Jan 2017	75	SMP	Surgical Specialists (Emergency medicine)	≥ 27	≥ 10	≤ 33	Y	NA	77	.	42.86	11.69	19.48
Yousef I M Hosny, 2006 [76]	Good	Egypt	.	M and F	.	72.4	.	.	≥27	≥ 13	≤31	N	NA	84	.	75	60.71	27.38
Yousef I M Hosny, 2006 [76]	Good	Egypt	.	M	.	72.4	.	.	≥27	≥ 13	≤31	N	NA	64	.	75	62.50	28.13
Yousef I M Hosny, 2006 [76]	Good	Egypt	.	F	.	72.4	.	.	≥27	≥ 13	≤31	N	NA	20	.	75	55.00	25.00
Zarei, Ehsan; 2019 [77]	Fair	Iran	.	M and F	2018		GMP	General medical practitioners (General practitioners)	>27	>10	<31	N	1	66	89.39	.	.	.
Zubairi, Akbar Jaleel; 2016 [78]	Poor	Pakistan	.	M and F	1st - 31st May 2013	34	SMP	All	.	.	.	Unclear	2	82	74.39	59.76	37.80	31.71
Zubairi, Akbar Jaleel; 2016 [78]	Poor	Pakistan	.	M and F	1st - 31st May 2013	34	SMP	All	.	.	.	Unclear	1	82	12.20	.	.	.

Zubairi, Akbar Jaleel; 2016 [78]	Poor	Pakistan	.	M and F	1st - 31st May 2013	34	SMP	All	.	.	.	Unclear	10	82	41.46	.	.	.

Gender: **M**-Male, **F**-Female
Category: **GMP**- Generalist Medical Practitioners, **SMP**- Specialist Medical Practitioners
Standard cut-off used- **Y**-Yes, **N**-No, **N/A**- Not Applicable
OB Definition Category:

1- High EE and High DP and Low PA
2- Any of the 3 (High EE or High DP or Low PA)
3- High EE+Low PA
4- High EE+ High DP
5- High DP or Low PA
6- High EE + (High DP or Low PA)
7- Low PA
8- High EE
9- High DP
10- 2 of the 3

Appendix S9: Metanalaysis Outputs

#Burnout: DP domain
##
DP: Prevalence in the EMR
##

Number of studies combined: k = 70

	proportion	95%-CI
Fixed effect model	0.4245	[0.4164; 0.4327]
Random effects model	0.3783	[0.3183; 0.4422]
Prediction interval		[0.0620; 0.8485]

Quantifying heterogeneity:
tau^2 = 1.2190; tau = 1.1041; I^2 = 97.9%; H = 6.90

Test of heterogeneity:

Q	d.f.	p-value	Test
1922.83	69	0	Wald-type
2572.40	69	0	Likelihood-Ratio

##
DP: Prevalence by country
##

Number of studies combined: k = 70

Results for subgroups (random effects model):

	k	proportion	95%-CI
Country = Oman	2	0.4109	[0.3698; 0.4532]
Country = Iran	7	0.2081	[0.0586; 0.5258]
Country = UAE	2	0.4750	[0.0741; 0.9110]
Country = Saudi arabia	8	0.4294	[0.3015; 0.5674]
Country = Yemen	1	0.1936	[0.1630; 0.2283]
Country = Saudi Arabia	11	0.3953	[0.3029; 0.4958]

Country = Lebanon	3	0.2191	[0.0942; 0.4309]
Country = Morocco	3	0.2547	[0.1486; 0.4009]
Country = Pakistan	8	0.3202	[0.2015; 0.4678]
Country = Kuwait	2	0.4582	[0.2171; 0.7207]
Country = Palestine	1	0.3169	[0.2457; 0.3978]
Country = Egypt	13	0.5782	[0.4490; 0.6975]
Country = Tunisia	2	0.2027	[0.1455; 0.2751]
Country = Iraq	3	0.4294	[0.1505; 0.7617]
Country = Syria	1	0.5460	[0.5291; 0.5628]
Country = Middle-East (Iran, Saudi Arabia ...	1	0.3000	[0.2614; 0.3416]
Country = Bahrain	1	0.2673	[0.2108; 0.3326]
Country = Qatar	1	0.2917	[0.2233; 0.3710]

	tau^2	tau	Q	I^2
Country = Oman	0	0	0.87	0.0%
Country = Iran	3.6677	1.9151	199.30	97.8%
Country = UAE	3.0101	1.7350	111.91	98.3%
Country = Saudi arabia	0.5690	0.7544	77.98	91.7%
Country = Yemen	--	--	0.00	--
Country = Saudi Arabia	0.4440	0.6663	205.14	94.3%
Country = Lebanon	0.6890	0.8301	46.31	91.8%
Country = Morocco	0.3140	0.5604	26.73	89.0%
Country = Pakistan	0.7300	0.8544	198.93	95.4%
Country = Kuwait	0.6009	0.7752	27.01	92.7%
Country = Palestine	--	--	0.00	--
Country = Egypt	0.8475	0.9206	213.50	95.4%
Country = Tunisia	0	0	0.28	0.0%
Country = Iraq	1.6072	1.2678	209.16	98.4%
Country = Syria	--	--	0.00	--
Country = Middle-East (Iran, Saudi Arabia ...	--	--	0.00	--
Country = Bahrain	--	--	0.00	--
Country = Qatar	--	--	0.00	--

Test for subgroup differences (random effects model):

	Q	d.f.	p-value
Between groups	406.22	17	< 0.0001

```
################################################################################
DP: Prevalence in the EMR by cut off
################################################################################

Number of studies combined: k = 70

Results for subgroups (random effects model):
                    k proportion           95%-CI  tau^2    tau      Q   I^2
DP_cutoff = Lower    7     0.3123 [0.2073; 0.4409] 0.5248 0.7244 270.44 96.9%
DP_cutoff = 10      13     0.4722 [0.3282; 0.6209] 1.1805 1.0865 393.12 96.7%
DP_cutoff = .       24     0.3963 [0.2813; 0.5241] 1.6054 1.2670 521.21 97.5%
DP_cutoff = Higher  26     0.3368 [0.2571; 0.4270] 0.9358 0.9674 518.20 97.1%

Test for subgroup differences (random effects model):
                   Q d.f. p-value
Between groups   3.41    3  0.3326



################################################################################
DP: Prevalence in the EMR by Gender
################################################################################

Number of studies combined: k = 25

Results for subgroups (random effects model):
                 k proportion           95%-CI  tau^2    tau      Q   I^2
Gender = Female  12     0.3448 [0.2203; 0.4950] 1.1055 1.0514 162.24 95.6%
Gender = Male    13     0.4034 [0.2814; 0.5388] 0.9443 0.9718 291.15 97.0%

Test for subgroup differences (random effects model):
                   Q d.f. p-value
Between groups   0.35    1  0.5527


################################################################################
DP: Prevalence in the EMR by professional category
################################################################################
```

```
Number of studies combined: k = 98

Results for subgroups (random effects model):
                 k proportion           95%-CI  tau^2    tau      Q   I^2
Category = GMP  32     0.4178 [0.3185; 0.5244] 1.4777 1.2156 832.10 99.2%
Category = SMP  66     0.4282 [0.3782; 0.4796] 0.6752 0.8217 847.20 95.3%

Test for subgroup differences (random effects model):
                    Q d.f. p-value
Between groups   0.03    1  0.8622


################################################################################
DP: Prevalence in the EMR by profesional subcategory
################################################################################

Number of studies combined: k = 98

Results for subgroups (random effects model):
                                              k proportion           95%-CI
Subcat = General Practitioners               31     0.4248 [0.3224; 0.5340]
Subcat = Surgical Specialists                40     0.4332 [0.3594; 0.5102]
Subcat = Medical Specialists                 17     0.4107 [0.3466; 0.4780]
Subcat = General Paediatricians               4     0.4415 [0.2988; 0.5945]
Subcat = OBGYN                                4     0.3790 [0.2432; 0.5369]
Subcat = Other specialists not elsewhere ...  1     0.2500 [0.1442; 0.3974]
Subcat = Psychiatrists                        1     0.6049 [0.4952; 0.7051]
                                             tau^2    tau      Q   I^2
Subcat = General Practitioners               1.5018 1.2255 814.85 99.2%
Subcat = Surgical Specialists                0.9236 0.9610 648.17 96.5%
Subcat = Medical Specialists                 0.2789 0.5281 107.03 89.6%
Subcat = General Paediatricians              0.3237 0.5690  15.95 84.7%
Subcat = OBGYN                               0.3600 0.6000  35.05 86.2%
Subcat = Other specialists not elsewhere ...     --     --   0.00    --
Subcat = Psychiatrists                           --     --   0.00    --

Test for subgroup differences (random effects model):
                     Q d.f. p-value
Between groups   15.97    6  0.0139
```

```
##########################################################################################
DP: Prevalence in the EMR by specialty
##########################################################################################
Number of studies combined: k = 66

Results for subgroups (random effects model):
                                                  k proportion           95%-CI
Specialty = Emergency medicine                    7     0.3226 [0.2376; 0.4213]
Specialty = Oto-rhino-laryngology                 3     0.5012 [0.4177; 0.5846]
Specialty = Plastic Surgery                       3     0.4673 [0.3750; 0.5619]
Specialty = Ophthalmology                         2     0.3792 [0.1193; 0.7336]
Specialty = Anaesthesiology                      14     0.5096 [0.4082; 0.6103]
Specialty = Medical Interns                       1     0.7093 [0.6591; 0.7549]
Specialty = Not reported                         19     0.4108 [0.3018; 0.5293]
Specialty = Oncology                              4     0.3706 [0.2335; 0.5323]
Specialty = Orthopaedics                          2     0.3975 [0.3380; 0.4603]
Specialty = General Surgery                       2     0.4464 [0.2617; 0.6471]
Specialty = Internal Medicine                     2     0.4074 [0.2295; 0.6135]
Specialty = Dermatology                           1     0.5556 [0.4464; 0.6596]
Specialty = Radiology                             1     0.4526 [0.3833; 0.5239]
Specialty = Neurology                             1     0.5062 [0.3987; 0.6131]
Specialty = other types of surgery                2     0.1351 [0.0743; 0.2333]
Specialty = Internal Medicine, Community me ...   1     0.4257 [0.3743; 0.4786]
Specialty = Intensive care Physicians             1     0.7007 [0.6219; 0.7692]


                                                   tau^2    tau      Q   I^2
Specialty = Emergency medicine                    0.2785 0.5277  47.95 88.5%
Specialty = Oto-rhino-laryngology                 0.0403 0.2008   5.41 45.2%
Specialty = Plastic Surgery                            0      0   1.12  0.0%
Specialty = Ophthalmology                         1.1401 1.0678  56.28 96.5%
Specialty = Anaesthesiology                       0.5440 0.7376 135.17 93.9%
Specialty = Medical Interns                           --     --   0.00    --
Specialty = Not reported                          1.0524 1.0258 232.57 95.3%
Specialty = Oncology                              0.3739 0.6115  15.42 84.9%
Specialty = Orthopaedics                               0      0   0.01  0.0%
Specialty = General Surgery                       0.3116 0.5582  18.43 89.3%
```

```
Specialty = Internal Medicine                  0.3280 0.5727  20.50 90.4%
Specialty = Dermatology                            --     --   0.00    --
Specialty = Radiology                            --     --   0.00    --
Specialty = Neurology                            --     --   0.00    --
Specialty = other types of surgery                 0      0   0.08  0.0%
Specialty = Internal Medicine, Community me ...     --     --   0.00    --
Specialty = Intensive care Physicians               --     --   0.00    --

Test for subgroup differences (random effects model):
                     Q d.f.  p-value
Between groups   146.87   16 < 0.0001



################################################################################
DP: Prevalence in Egypt
################################################################################
> Country level

Number of studies combined: k = 13

                     proportion           95%-CI
Fixed effect model       0.4871 [0.4623; 0.5120]
Random effects model     0.5782 [0.4490; 0.6975]
Prediction interval             [0.1426; 0.9186]

Quantifying heterogeneity:
 tau^2 = 0.8475; tau = 0.9206; I^2 = 95.4%; H = 4.66

Test of heterogeneity:
      Q d.f.  p-value             Test
 213.50   12 < 0.0001        Wald-type
 264.37   12 < 0.0001 Likelihood-Ratio

> Prevalence by gender

Number of studies combined: k = 7

Results for subgroups (random effects model):
```

```
                  k proportion           95%-CI  tau^2    tau     Q   I^2
Gender = Male     4     0.6104 [0.4745; 0.7311] 0.2395 0.4894 17.95 78.5%
Gender = Female   3     0.5863 [0.3859; 0.7618] 0.3673 0.6060 10.35 70.9%

Test for subgroup differences (random effects model):
                  Q d.f. p-value
Between groups   0.04    1  0.8416


################################################################################
DP: Prevalence in Iraq
################################################################################

> Country level
Number of studies combined: k = 3

                     proportion           95%-CI
Fixed effect model       0.4608 [0.4252; 0.4969]
Random effects model     0.4294 [0.1505; 0.7617]
Prediction interval             [0.0000; 1.0000]

Quantifying heterogeneity:
 tau^2 = 1.6072; tau = 1.2678; I^2 = 98.4%; H = 8.00

Test of heterogeneity:
      Q d.f.  p-value             Test
 209.16    2 < 0.0001        Wald-type
 277.08    2 < 0.0001 Likelihood-Ratio

################################################################################
DP: Prevalence in Pakistan
################################################################################

Number of studies combined: k = 8

                     proportion           95%-CI
Fixed effect model       0.4329 [0.4061; 0.4601]
Random effects model     0.3202 [0.2015; 0.4678]
Prediction interval             [0.0482; 0.8143]
```

```
Quantifying heterogeneity:
 tau^2 = 0.7300; tau = 0.8544; I^2 = 95.4%; H = 4.66

Test of heterogeneity:
      Q d.f.  p-value             Test
 198.93    7 < 0.0001         Wald-type
 231.97    7 < 0.0001 Likelihood-Ratio


> Prevalence by gender

Number of studies combined: k = 4

Results for subgroups (random effects model):
                  k proportion            95%-CI  tau^2     tau      Q   I^2
Gender = Male     2     0.4007 [0.0942; 0.8111] 1.6747 1.2941 27.33 93.0%
Gender = Female   2     0.1934 [0.0029; 0.9517] 5.2976 2.3016  0.00 82.9%

Test for subgroup differences (random effects model):
                    Q d.f. p-value
Between groups   0.18    1  0.6745


################################################################################
DP: Prevalence in Saudi Arabia
################################################################################
> Country level

Number of studies combined: k = 19

                     proportion           95%-CI
Fixed effect model       0.3940 [0.3761; 0.4122]
Random effects model     0.4086 [0.3316; 0.4904]
Prediction interval             [0.1307; 0.7606]

Quantifying heterogeneity:
 tau^2 = 0.4942; tau = 0.7030; I^2 = 93.8%; H = 4.01
```

```
Test of heterogeneity:
      Q d.f.  p-value             Test
 283.78   18 < 0.0001        Wald-type
 328.91   18 < 0.0001 Likelihood-Ratio


> Prevalence by gender

Results for subgroups (random effects model):
                  k proportion           95%-CI  tau^2    tau     Q   I^2
Gender = Male     2     0.2652 [0.1312; 0.4631] 0.3591 0.5992 22.15 91.0%
Gender = Female   2     0.2178 [0.0936; 0.4289] 0.4058 0.6370  9.05 78.7%

Test for subgroup differences (random effects model):
                    Q d.f. p-value
Between groups   0.15    1  0.7006



################################################################################
DP: Prevalence in Iran
################################################################################
> Country level

Number of studies combined: k = 7

                     proportion           95%-CI
Fixed effect model       0.2896 [0.2607; 0.3202]
Random effects model     0.2081 [0.0586; 0.5258]
Prediction interval             [0.0013; 0.9809]

Quantifying heterogeneity:
 tau^2 = 3.6677; tau = 1.9151; I^2 = 97.8%; H = 6.68

Test of heterogeneity:
      Q d.f.  p-value             Test
 199.30    6 < 0.0001        Wald-type
 456.95    6 < 0.0001 Likelihood-Ratio
```

```
> Prevalence by gender

Number of studies combined: k = 3

Results for subgroups (random effects model):
                k proportion           95%-CI tau^2 tau    Q  I^2
Gender = Female   2     0.1318 [0.0835; 0.2018]     0   0 0.37 0.0%
Gender = Male     1     0.1667 [0.0891; 0.2903]    --  -- 0.00   --

Test for subgroup differences (random effects model):
                  Q d.f. p-value
Between groups   0.38    1  0.5385


#####################################################################################
DP: Prevalence in  Morocco
#####################################################################################

Number of studies combined: k = 3

                    proportion           95%-CI
Fixed effect model       0.2412 [0.2039; 0.2827]
Random effects model     0.2547 [0.1486; 0.4009]
Prediction interval             [0.0001; 0.9993]

Quantifying heterogeneity:
 tau^2 = 0.3140; tau = 0.5604; I^2 = 89.0%; H = 3.02

Test of heterogeneity:
     Q d.f.  p-value             Test
 26.73    2 < 0.0001        Wald-type
 26.37    2 < 0.0001 Likelihood-Ratio
```

#Burnout: EE domain

##

EE: Prevalence in the EMR

##

Number of studies combined: k = 70

	proportion	95%-CI
Fixed effect model	0.5190	[0.5108; 0.5272]
Random effects model	0.4426	[0.3799; 0.5072]
Prediction interval		[0.0826; 0.8750]

Quantifying heterogeneity:

tau^2 = 1.1722; tau = 1.0827; I^2 = 97.9%; H = 6.84

Test of heterogeneity:

Q	d.f.	p-value	Test
2147.42	69	0	Wald-type
3160.52	69	0	Likelihood-Ratio

###

EE: Prevalence by country

###

Number of studies combined: k = 70

Results for subgroups (random effects model):

	k	proportion	95%-CI
Country = Oman	2	0.1782	[0.1480; 0.2131]
Country = Iran	7	0.2309	[0.1449; 0.3473]
Country = UAE	2	0.6037	[0.3580; 0.8063]
Country = Saudi arabia	8	0.6141	[0.5092; 0.7094]
Country = Yemen	1	0.6323	[0.5917; 0.6712]
Country = Saudi Arabia	11	0.3705	[0.2670; 0.4875]
Country = Lebanon	3	0.4653	[0.2934; 0.6459]

Country = Morocco	3	0.6497	[0.4429; 0.8123]
Country = Pakistan	7	0.3822	[0.2852; 0.4896]
Country = Kuwait	2	0.3889	[0.1366; 0.7190]
Country = Palestine	1	0.7183	[0.6389; 0.7861]
Country = Egypt	13	0.6076	[0.5122; 0.6954]
Country = Tunisia	2	0.3106	[0.1942; 0.4572]
Country = Iraq	3	0.0778	[0.0004; 0.9437]
Country = Syria	1	0.7788	[0.7644; 0.7925]
Country = Middle-East (Iran, Saudi Arabia ...	1	0.4400	[0.3971; 0.4839]
Country = Qatar	2	0.2101	[0.0904; 0.4158]
Country = Bahrain	1	0.4307	[0.3641; 0.4999]

	tau^2	tau	Q	I^2
Country = Oman	0	0	0.00	0.0%
Country = Iran	0.5359	0.7320	44.51	92.2%
Country = UAE	0.4961	0.7044	35.23	94.4%
Country = Saudi arabia	0.3102	0.5569	51.19	86.3%
Country = Yemen	--	--	0.00	--
Country = Saudi Arabia	0.6198	0.7873	167.47	95.9%
Country = Lebanon	0.3807	0.6170	34.07	90.4%
Country = Morocco	0.5258	0.7251	49.14	93.9%
Country = Pakistan	0.3019	0.5494	44.07	91.5%
Country = Kuwait	0.9553	0.9774	36.86	94.7%
Country = Palestine	--	--	0.00	--
Country = Egypt	0.4484	0.6696	128.11	92.1%
Country = Tunisia	0.0161	0.1270	2.83	12.9%
Country = Iraq	18.5482	4.3068	9.47	99.7%
Country = Syria	--	--	0.00	--
Country = Middle-East (Iran, Saudi Arabia ...	--	--	0.00	--
Country = Qatar	0.4377	0.6616	14.81	86.7%
Country = Bahrain	--	--	0.00	--

Test for subgroup differences (random effects model):

	Q	d.f.	p-value
Between groups	857.49	17	< 0.0001

##

EE: Prevalence in the EMR by cut off
###

Number of studies combined: k = 70

Results for subgroups (random effects model):

	k	proportion	95%-CI	tau^2	tau	Q	I^2
EE_cutoff = Lower	5	0.4920	[0.2719; 0.7153]	1.1246	1.0605	120.66	96.9%
EE_cutoff = 27	24	0.4011	[0.2755; 0.5412]	1.9326	1.3902	436.05	98.4%
EE_cutoff = .	23	0.4958	[0.4084; 0.5836]	0.7044	0.8393	320.04	95.4%
EE_cutoff = Higher	18	0.4102	[0.3080; 0.5209]	0.8782	0.9371	1108.94	97.9%

Test for subgroup differences (random effects model):

	Q	d.f.	p-value
Between groups	2.14	3	0.5448

Details on meta-analytical method:
- Random intercept logistic regression model
- Maximum-likelihood estimator for tau^2
- Logit transformation
- Clopper-Pearson confidence interval for individual studies
- Continuity correction of 0.5 in studies with zero cell frequencies
  (only used to calculate individual study results)

###
EE: Prevalence in the EMR by Gender
###

Number of studies combined: k = 27

	proportion	95%-CI
Random effects model	0.4746	[0.3750; 0.5763]
Prediction interval		[0.0911; 0.8907]

Quantifying heterogeneity:
 tau^2 = 1.0967; tau = 1.0472; I^2 = 97.5%; H = 6.34

Quantifying residual heterogeneity:
 I^2 = 97.2% [96.6%; 97.7%]; H = 5.95 [5.38; 6.57]

Test of heterogeneity:

```
      Q d.f.  p-value           Test
  886.79   26 < 0.0001        Wald-type
 1106.79   26 < 0.0001 Likelihood-Ratio
```

Results for subgroups (random effects model):

```
                   k proportion           95%-CI  tau^2    tau      Q   I^2
Gender = Female  13     0.4826 [0.3512; 0.6166] 0.9165 0.9573 242.41 96.0%
Gender = Male    14     0.4666 [0.3232; 0.6157] 1.2610 1.1230 642.26 97.7%
```

Test for subgroup differences (random effects model):

```
                    Q d.f. p-value
Between groups   0.02    1  0.8768
```

###
EE: Prevalence in the EMR by profesional category
###

Number of studies combined: k = 80

Results for subgroups (random effects model):

```
                  k proportion           95%-CI  tau^2    tau       Q   I^2
Category = GMP  25     0.4179 [0.2754; 0.5756] 2.5606 1.6002 1170.30 99.1%
Category = SMP  55     0.4978 [0.4358; 0.5599] 0.8275 0.9097 1236.81 95.5%
```

Test for subgroup differences (random effects model):

```
                    Q d.f. p-value
Between groups   0.86    1  0.3546
```

###
EE: Prevalence in the EMR by professional subcategory
###
Number of studies combined: k = 80

Results for subgroups (random effects model):

```
                                         k proportion           95%-CI  tau^2    tau
Subcat = General Practitioners          23     0.4076 [0.2596; 0.5744] 2.6443 1.6261
Subcat = Surgical Specialists           31     0.4803 [0.4022; 0.5594] 0.7508 0.8665
```

```
Subcat = Medical Specialists                  16     0.5254 [0.3944; 0.6530] 1.1149 1.0559
Subcat = General Paediatricians                3     0.6157 [0.4118; 0.7856] 0.4580 0.6768
Subcat = OBGYN                                4     0.5207 [0.3272; 0.7081] 0.6072 0.7792
Subcat = General Paediatricians                1     0.4762 [0.3567; 0.5985]      --     --
Subcat = Other specialists not elsewhere ...   1     0.1591 [0.0778; 0.2979]      --     --
Subcat = Psychiatrists                        1     0.6667 [0.5575; 0.7604]      --     --
                                             Q   I^2
Subcat = General Practitioners              1101.49 99.1%
Subcat = Surgical Specialists                708.53 95.3%
Subcat = Medical Specialists                 463.31 96.6%
Subcat = General Paediatricians               30.31 88.3%
Subcat = OBGYN                               46.80 91.4%
Subcat = General Paediatricians                0.00    --
Subcat = Other specialists not elsewhere ...    0.00    --
Subcat = Psychiatrists                       0.00    --

Test for subgroup differences (random effects model):
                   Q d.f. p-value
Between groups   27.95    7  0.0002

#####################################################################################
EE: Prevalence in the EMR by Specialty
#####################################################################################

Number of studies combined: k = 55

Results for subgroups (random effects model):
                                          k proportion          95%-CI
Specialty = Emergency medicine                  7     0.5926 [0.4048; 0.7568]
Specialty = Oto-rhino-laryngology               2     0.7032 [0.5849; 0.7995]
Specialty = Plastic Surgery                   2     0.6957 [0.5778; 0.7924]
Specialty = Ophthalmology                       2     0.5894 [0.3014; 0.8269]
Specialty = Anaesthesiology                     6     0.3946 [0.2747; 0.5286]
Specialty = Medical Interns                   1     0.4157 [0.3647; 0.4685]
Specialty = Not reported                     18     0.4204 [0.3303; 0.5162]
Specialty = Oncology                         4     0.5753 [0.4304; 0.7083]
Specialty = Orthopaedics                      2     0.4245 [0.3196; 0.5368]
Specialty = General Surgery                     2     0.5826 [0.2154; 0.8764]
Specialty = Neurology                        1     0.8519 [0.7570; 0.9139]
```

```
Specialty = Internal Medicine                      2     0.6030 [0.3410; 0.8168]
Specialty = Dermatology                            1     0.7654 [0.6612; 0.8451]
Specialty = Radiology                              1     0.6474 [0.5769; 0.7120]
Specialty = other types of surgery                 1     0.1750 [0.0858; 0.3241]
Specialty = Internal Medicine, Community me ...    1     0.1778 [0.1409; 0.2220]
Specialty = Intensive care Physicians              2     0.3774 [0.3146; 0.4445]

                                                tau^2    tau      Q   I^2
Specialty = Emergency medicine                 1.0044 1.0022 153.62 96.2%
Specialty = Oto-rhino-laryngology              0.0811 0.2847   4.61 57.2%
Specialty = Plastic Surgery                         0      0   0.09  0.0%
Specialty = Ophthalmology                      0.7199 0.8485  45.54 95.7%
Specialty = Anaesthesiology                    0.3728 0.6106  29.32 84.7%
Specialty = Medical Interns                        --     --   0.00    --
Specialty = Not reported                       0.6288 0.7930 191.55 91.8%
Specialty = Oncology                           0.2877 0.5363  17.66 81.8%
Specialty = Orthopaedics                       0.0703 0.2652   5.88 66.2%
Specialty = General Surgery                    1.3369 1.1563  70.29 97.2%
Specialty = Neurology                              --     --   0.00    --
Specialty = Internal Medicine                  0.5733 0.7572  40.40 95.1%
Specialty = Dermatology                            --     --   0.00    --
Specialty = Radiology                              --     --   0.00    --
Specialty = other types of surgery                 --     --   0.00    --
Specialty = Internal Medicine, Community me ...    --     --   0.00    --
Specialty = Intensive care Physicians               0      0   0.22  0.0%

Test for subgroup differences (random effects model):
                    Q d.f.  p-value
Between groups   240.49   16 < 0.0001

#####################################################################################
EE: Prevalence in Egypt
#####################################################################################
> Country level

Number of studies combined: k = 13

                  proportion           95%-CI
Fixed effect model       0.5264 [0.5015; 0.5511]
```

```
Random effects model     0.6076 [0.5122; 0.6954]
Prediction interval             [0.2498; 0.8780]

Quantifying heterogeneity:
 tau^2 = 0.4484; tau = 0.6696; I^2 = 92.1%; H = 3.57

Test of heterogeneity:
      Q d.f.  p-value             Test
 128.11   12 < 0.0001        Wald-type
 145.30   12 < 0.0001 Likelihood-Ratio


> Prevalence by Gender

Number of studies combined: k = 7

Results for subgroups (random effects model):
                  k proportion           95%-CI  tau^2    tau     Q   I^2
Gender = Male     4     0.6888 [0.5415; 0.8057] 0.3293 0.5738 26.19 81.5%
Gender = Female   3     0.6588 [0.4547; 0.8172] 0.3835 0.6193 10.43 70.1%

Test for subgroup differences (random effects model):
                    Q d.f. p-value
Between groups   0.07    1  0.7983

#################################################################################
EE: Prevalence in Iraq
#################################################################################

Number of studies combined: k = 3

                     proportion           95%-CI
Fixed effect model       0.3351 [0.3020; 0.3700]
Random effects model     0.0778 [0.0004; 0.9437]
Prediction interval             [0.0000; 1.0000]

Quantifying heterogeneity:
 tau^2 = 18.5482; tau = 4.3068; I^2 = 99.7%; H = 19.82
```

```
Test of heterogeneity:
      Q d.f.  p-value             Test
   9.47    2   0.0088        Wald-type
 367.75    2 < 0.0001 Likelihood-Ratio

##################################################################################
EE: Prevalence in Iran
##################################################################################
> Country level

Number of studies combined: k = 7

                     proportion           95%-CI
Fixed effect model       0.2565 [0.2288; 0.2862]
Random effects model     0.2309 [0.1449; 0.3473]
Prediction interval             [0.0381; 0.6948]

Quantifying heterogeneity:
 tau^2 = 0.5359; tau = 0.7320; I^2 = 92.2%; H = 3.57

Test of heterogeneity:
     Q d.f.  p-value             Test
 44.51    6 < 0.0001        Wald-type
 61.60    6 < 0.0001 Likelihood-Ratio

> Prevalence by Gender

Number of studies combined: k = 3

Results for subgroups (random effects model):
                  k proportion           95%-CI  tau^2    tau     Q   I^2
Gender = Female   2     0.1835 [0.0356; 0.5777] 1.4999 1.2247 15.43 88.1%
Gender = Male     1     0.0370 [0.0093; 0.1364]     --     --  0.00    --

Test for subgroup differences (random effects model):
                    Q d.f. p-value
Between groups   2.28    1  0.1312
```

##
EE: Prevalence in Morocco
##

Number of studies combined: k = 3

```
                     proportion           95%-CI
Fixed effect model       0.6571 [0.6121; 0.6994]
Random effects model     0.6497 [0.4429; 0.8123]
Prediction interval             [0.0000; 1.0000]
```

Quantifying heterogeneity:
tau^2 = 0.5258; tau = 0.7251; I^2 = 93.9%; H = 4.05

Test of heterogeneity:

```
     Q d.f.  p-value             Test
 49.14    2 < 0.0001        Wald-type
 56.14    2 < 0.0001 Likelihood-Ratio
```

##
EE: Prevalence in Pakistan
##

> Country level

Number of studies combined: k = 7

```
                     proportion           95%-CI
Fixed effect model       0.3920 [0.3654; 0.4193]
Random effects model     0.3822 [0.2852; 0.4896]
Prediction interval             [0.1186; 0.7398]
```

Quantifying heterogeneity:
tau^2 = 0.3019; tau = 0.5494; I^2 = 91.5%; H = 3.44

Test of heterogeneity:

```
     Q d.f.  p-value             Test
 44.07    6 < 0.0001        Wald-type
 48.62    6 < 0.0001 Likelihood-Ratio
```

```
> Prevalence by gender

Number of studies combined: k = 5

Results for subgroups (random effects model):
                  k proportion           95%-CI  tau^2    tau    Q   I^2
Gender = Male     3     0.3315 [0.2341; 0.4459] 0.0879 0.2966 7.98 67.9%
Gender = Female   2     0.3912 [0.3437; 0.4408]      0       0 1.10  0.0%

Test for subgroup differences (random effects model):
                    Q d.f. p-value
Between groups   0.93    1  0.3341


####################################################################################
EE: Prevalence in Saudi Arabia
####################################################################################
> Country level

Number of studies combined: k = 19

                     proportion           95%-CI
Fixed effect model       0.4492 [0.4309; 0.4677]
Random effects model     0.4700 [0.3734; 0.5689]
Prediction interval             [0.1223; 0.8495]

Quantifying heterogeneity:
 tau^2 = 0.7282; tau = 0.8533; I^2 = 95.8%; H = 4.88

Test of heterogeneity:
      Q d.f.  p-value             Test
 275.78   18 < 0.0001        Wald-type
 349.30   18 < 0.0001 Likelihood-Ratio


> Prevalence by gender
```

Number of studies combined: k = 4

Results for subgroups (random effects model):

```
                 k proportion           95%-CI  tau^2    tau     Q   I^2
Gender = Male     2     0.3979 [0.2687; 0.5432] 0.1476 0.3842 11.71 82.9%
Gender = Female   2     0.3827 [0.1745; 0.6451] 0.5244 0.7241 14.76 86.8%
```

Test for subgroup differences (random effects model):

```
                   Q d.f. p-value
Between groups   0.01    1  0.9184
```

##

EE: Prevalence in Lebanon

##

> Country level

Number of studies combined: k = 3

Quantifying heterogeneity:

tau^2 = 0.3807; tau = 0.6170; I^2 = 90.4%; H = 3.24

Test of heterogeneity:

```
     Q d.f.  p-value             Test
 34.07    2 < 0.0001        Wald-type
 36.09    2 < 0.0001 Likelihood-Ratio
```

> Prevalence by gender

Number of studies combined: k = 4

Results for subgroups (random effects model):

```
                 k proportion           95%-CI  tau^2    tau     Q   I^2
Gender = Male     2     0.4739 [0.2872; 0.6682] 0.2893 0.5379 13.76 85.7%
Gender = Female   2     0.6118 [0.3822; 0.8006] 0.3813 0.6175 11.82 83.5%
```

Test for subgroup differences (random effects model):

```
                   Q d.f. p-value
Between groups   0.79    1  0.3740
```

#Burnout: PA domain

##
PA: Prevalence in the EMR
##

Number of studies combined: k = 70

	proportion	95%-CI
Fixed effect model	0.3314	[0.3237; 0.3392]
Random effects model	0.3657	[0.3150; 0.4195]
Prediction interval		[0.0804; 0.7917]

Quantifying heterogeneity:
tau^2 = 0.8801; tau = 0.9382; I^2 = 96.9%; H = 5.72

Test of heterogeneity:

Q	d.f.	p-value	Test
1878.95	69	0	Wald-type
2657.18	69	0	Likelihood-Ratio

##
PA: Prevalence in the EMR by country
##

Number of studies combined: k = 70

Results for subgroups (random effects model):

	k	proportion	95%-CI
Country = Oman	2	0.3068	[0.1891; 0.4565]
Country = Iran	7	0.3686	[0.1987; 0.5788]
Country = UAE	2	0.4808	[0.1574; 0.8211]
Country = Saudi arabia	8	0.3239	[0.2627; 0.3917]
Country = Yemen	1	0.4156	[0.3756; 0.4568]
Country = Saudi Arabia	13	0.3539	[0.2625; 0.4573]
Country = Lebanon	3	0.3706	[0.1513; 0.6605]

```
Country = Morocco                          3     0.2342 [0.1230; 0.4001]
Country = Pakistan                         7     0.3886 [0.2832; 0.5056]
Country = Kuwait                           2     0.4690 [0.2845; 0.6624]
Country = Palestine                        1     0.3169 [0.2457; 0.3978]
Country = Egypt                           13     0.4614 [0.2717; 0.6629]
Country = Tunisia                          2     0.3784 [0.3039; 0.4590]
Country = Iraq                             2     0.2605 [0.2212; 0.3040]
Country = Syria                            1     0.1367 [0.1255; 0.1488]
Country = Middle-East (Iran, Saudi Arabia ...   1     0.2880 [0.2500; 0.3293]
Country = Bahrain                          1     0.2228 [0.1707; 0.2853]
Country = Qatar                            1     0.5833 [0.5013; 0.6610]
```

```
                                         tau^2    tau      Q   I^2
Country = Oman                          0.1920 0.4382  19.56 89.9%
Country = Iran                          1.2835 1.1329 182.56 96.7%
Country = UAE                           1.2973 1.1390  70.88 97.2%
Country = Saudi arabia                  0.1150 0.3391  19.92 69.0%
Country = Yemen                             --     --   0.00    --
Country = Saudi Arabia                  0.5889 0.7674 264.13 95.4%
Country = Lebanon                       1.0588 1.0290  48.48 95.4%
Country = Morocco                       0.4306 0.6562  39.39 91.1%
Country = Pakistan                      0.3598 0.5998  66.44 92.9%
Country = Kuwait                        0.2885 0.5371  14.99 86.9%
Country = Palestine                         --     --   0.00    --
Country = Egypt                         2.2601 1.5034 245.44 97.7%
Country = Tunisia                            0      0   0.80  0.0%
Country = Iraq                               0      0   0.00  0.0%
Country = Syria                             --     --   0.00    --
Country = Middle-East (Iran, Saudi Arabia ...     --     --   0.00    --
Country = Bahrain                           --     --   0.00    --
Country = Qatar                             --     --   0.00    --

Test for subgroup differences (random effects model):
                    Q d.f.  p-value
Between groups   422.81   17 < 0.0001
```

##

PA: Prevalence in the EMR by cut off
##

Number of studies combined: k = 70

Results for subgroups (random effects model):

	k	proportion	95%-CI	tau^2	tau	Q	I^2
PA_cutoff = 33	16	0.2761	[0.2224; 0.3372]	0.2935	0.5417	196.48	89.3%
PA_cutoff = .	25	0.3799	[0.3017; 0.4648]	0.7473	0.8645	511.54	95.6%
PA_cutoff = Lower	24	0.4320	[0.3181; 0.5536]	1.4373	1.1989	1008.71	98.6%
PA_cutoff = Higher	5	0.3161	[0.2247; 0.4243]	0.2251	0.4744	26.79	83.2%

Test for subgroup differences (random effects model):

	Q	d.f.	p-value
Between groups	7.63	3	0.0543

##
PA: Prevalence in the EMR by gender
##

Number of studies combined: k = 25

Results for subgroups (random effects model):

	k	proportion	95%-CI	tau^2	tau	Q	I^2
Gender = Female	12	0.3208	[0.2297; 0.4279]	0.5722	0.7564	252.89	91.9%
Gender = Male	13	0.3285	[0.2555; 0.4109]	0.3704	0.6086	303.05	92.5%

Test for subgroup differences (random effects model):

	Q	d.f.	p-value
Between groups	0.01	1	0.9053

##
PA: Prevalence in the EMR by professional category
##
Number of studies combined: k = 92

```
Results for subgroups (random effects model):
                 k proportion           95%-CI  tau^2    tau       Q   I^2
Category = GMP  31     0.2829 [0.2279; 0.3452] 0.6333 0.7958  892.82 97.2%
Category = SMP  61     0.2994 [0.2464; 0.3584] 1.0606 1.0298 1272.43 96.6%

Test for subgroup differences (random effects model):
                    Q d.f. p-value
Between groups   0.16    1  0.6910



################################################################################
PA: Prevalence in the EMR by professional subcategory
################################################################################

Number of studies combined: k = 92

ts for subgroups (random effects model):
                                 k proportion           95%-CI  tau^2    tau
Subcat = General Practitioners   31     0.2829 [0.2279; 0.3452] 0.6333 0.7958
Subcat = Surgical Specialists    38     0.3984 [0.3379; 0.4622] 0.6110 0.7817
Subcat = Medical Specialists     16     0.1885 [0.1185; 0.2863] 1.1504 1.0726
Subcat = General Paediatricians   3     0.1914 [0.0965; 0.3441] 0.3911 0.6254
Subcat = OBGYN                    3     0.1259 [0.0926; 0.1689]      0      0
Subcat = Psychiatrists            1     0.0370 [0.0120; 0.1086]     --     --
                                      Q   I^2
Subcat = General Practitioners  892.82 97.2%
Subcat = Surgical Specialists   654.57 95.0%
Subcat = Medical Specialists    281.16 95.6%
Subcat = General Paediatricians  13.62 80.5%
Subcat = OBGYN                    0.89  0.0%
Subcat = Psychiatrists            0.00    --

Test for subgroup differences (random effects model):
                    Q d.f.  p-value
Between groups   66.38    5 < 0.0001
```

##
PA: Prevalence in the EMR by specialty
##

Number of studies combined: k = 61

Results for subgroups (random effects model):

	k	proportion	95%-CI
Specialty = Emergency medicine	7	0.3259	[0.1961; 0.4893]
Specialty = Oto-rhino-laryngology	3	0.1514	[0.1122; 0.2012]
Specialty = Plastic Surgery	3	0.3664	[0.2443; 0.5086]
Specialty = Ophthalmology	2	0.4213	[0.2878; 0.5674]
Specialty = Anaesthesiology	13	0.4621	[0.4238; 0.5009]
Specialty = Medical Interns	1	0.5320	[0.4791; 0.5842]
Specialty =	15	0.2682	[0.1508; 0.4307]
Specialty = Oncology	4	0.2511	[0.0709; 0.5958]
Specialty = Orthopaedics	2	0.2315	[0.0899; 0.4790]
Specialty = General Surgery	2	0.1841	[0.1177; 0.2764]
Specialty = Internal Medicine	2	0.1472	[0.1254; 0.1720]
Specialty = Dermatology	1	0.1358	[0.0768; 0.2288]
Specialty = Radiology	1	0.0579	[0.0323; 0.1015]
Specialty = Neurology	1	0.0494	[0.0187; 0.1243]
Specialty = other types of surgery	2	0.3784	[0.2756; 0.4933]
Specialty = Internal Medicine, Community me ...	1	0.4082	[0.3574; 0.4610]
Specialty = Intensive care Physicians	1	0.5102	[0.4298; 0.5901]

	tau^2	tau	Q	I^2
Specialty = Emergency medicine	0.8007	0.8948	129.89	95.1%
Specialty = Oto-rhino-laryngology	0	0	0.72	0.0%
Specialty = Plastic Surgery	0.1393	0.3732	6.14	52.0%
Specialty = Ophthalmology	0.1547	0.3933	14.26	86.1%
Specialty = Anaesthesiology	0.0302	0.1738	25.29	50.4%
Specialty = Medical Interns	--	--	0.00	--
Specialty = Not reported	1.9353	1.3912	339.52	96.7%
Specialty = Oncology	2.1465	1.4651	52.86	95.3%
Specialty = Orthopaedics	0.5868	0.7660	20.29	90.4%
Specialty = General Surgery	0.0820	0.2864	6.69	66.3%
Specialty = Internal Medicine	0	0	0.21	0.0%
Specialty = Dermatology	--	--	0.00	--

```
Specialty = Radiology                          --     --   0.00    --
Specialty = Neurology                          --     --   0.00    --
Specialty = other types of surgery              0      0   0.17  0.0%
Specialty = Internal Medicine, Community me ...     --     --   0.00    --
Specialty = Intensive care Physicians          --     --   0.00    --

Test for subgroup differences (random effects model):
                    Q d.f.  p-value
Between groups   383.98   16 < 0.0001

################################################################################
PA: Prevalence in Egypt
################################################################################

> Country level

Number of studies combined: k = 13

                     proportion           95%-CI
Fixed effect model       0.4665 [0.4418; 0.4914]
Random effects model     0.4614 [0.2717; 0.6629]
Prediction interval             [0.0268; 0.9638]

Quantifying heterogeneity:
 tau^2 = 2.2601; tau = 1.5034; I^2 = 97.7%; H = 6.66

Test of heterogeneity:
      Q d.f.  p-value             Test
 245.44   12 < 0.0001        Wald-type
 626.00   12 < 0.0001 Likelihood-Ratio


> Prevalence by gender

Number of studies combined: k = 7

Results for subgroups (random effects model):
                  k proportion           95%-CI  tau^2    tau    Q   I^2
Gender = Male     4     0.4135 [0.3274; 0.5052] 0.0777 0.2787 8.55 55.3%
```

```
Gender = Female   3     0.4105 [0.3164; 0.5117]      0       0 2.76  0.0%

Test for subgroup differences (random effects model):
                  Q d.f. p-value
Between groups   0.00    1  0.9653

#################################################################################
PA: Prevalence in Iraq
#################################################################################

Number of studies combined: k = 3

                     proportion           95%-CI
Fixed effect model       0.2243 [0.1957; 0.2558]
Random effects model     0.2265 [0.1808; 0.2799]
Prediction interval             [0.0134; 0.8631]

Quantifying heterogeneity:
 tau^2 = 0.0374; tau = 0.1935; I^2 = 60.3%; H = 1.59

Test of heterogeneity:
    Q d.f. p-value             Test
 7.62    2  0.0222        Wald-type
 7.86    2  0.0196 Likelihood-Ratio

#################################################################################
PA: Prevalence in Pakistan
#################################################################################

> Country level

Number of studies combined: k = 7

                     proportion           95%-CI
Fixed effect model       0.4693 [0.4418; 0.4970]
Random effects model     0.3886 [0.2832; 0.5056]
Prediction interval             [0.1075; 0.7703]

Quantifying heterogeneity:
```

```
 tau^2 = 0.3598; tau = 0.5998; I^2 = 92.9%; H = 3.75

Test of heterogeneity:
     Q d.f.  p-value             Test
 66.44    6 < 0.0001        Wald-type
 76.29    6 < 0.0001 Likelihood-Ratio


> Prevalence by gender

Number of studies combined: k = 3

Results for subgroups (random effects model):
                  k proportion           95%-CI tau^2 tau    Q  I^2
Gender = Male     2     0.4835 [0.4302; 0.5371]     0   0 2.53 0.0%
Gender = Female   1     0.5135 [0.4333; 0.5930]    --  -- 0.00   --

Test for subgroup differences (random effects model):
                    Q d.f. p-value
Between groups   0.37    1  0.5433

################################################################################
PA: Prevalence in Saudi Arabia
################################################################################

> Country level

Number of studies combined: k = 21

                     proportion           95%-CI
Fixed effect model       0.3538 [0.3373; 0.3706]
Random effects model     0.3446 [0.2804; 0.4151]
Prediction interval             [0.1123; 0.6861]

Quantifying heterogeneity:
 tau^2 = 0.4400; tau = 0.6633; I^2 = 93.0%; H = 3.79

Test of heterogeneity:
      Q d.f.  p-value             Test
```

```
 290.32   20 < 0.0001        Wald-type
 330.83   20 < 0.0001 Likelihood-Ratio

> Prevalence by gender

Number of studies combined: k = 4

Results for subgroups (random effects model):
                  k proportion           95%-CI  tau^2    tau    Q   I^2
Gender = Male     2     0.2097 [0.1722; 0.2529]      0      0 1.11  0.0%
Gender = Female   2     0.2602 [0.1722; 0.3729] 0.0580 0.2408 3.28 39.8%

Test for subgroup differences (random effects model):
                    Q d.f. p-value
Between groups   0.91    1  0.3407

################################################################################
PA: Prevalence in Morocco
################################################################################>


Number of studies combined: k = 3

                     proportion           95%-CI
Fixed effect model       0.2478 [0.2102; 0.2897]
Random effects model     0.2342 [0.1230; 0.4001]
Prediction interval             [0.0000; 0.9998]

Quantifying heterogeneity:
 tau^2 = 0.4306; tau = 0.6562; I^2 = 91.1%; H = 3.35

Test of heterogeneity:
     Q d.f.  p-value             Test
 39.39    2 < 0.0001        Wald-type
 41.73    2 < 0.0001 Likelihood-Ratio

################################################################################
PA: Prevalence in Iran
################################################################################
```

> Country level

Number of studies combined: k = 7

```
                     proportion           95%-CI
Fixed effect model       0.4289 [0.3967; 0.4617]
Random effects model     0.3686 [0.1987; 0.5788]
Prediction interval             [0.0251; 0.9298]
```

Quantifying heterogeneity:
tau^2 = 1.2835; tau = 1.1329; I^2 = 96.7%; H = 5.51

Test of heterogeneity:

```
      Q d.f.  p-value             Test
 182.56    6 < 0.0001        Wald-type
 228.32    6 < 0.0001 Likelihood-Ratio
```

> Prevalence by gender

Number of studies combined: k = 3

Results for subgroups (random effects model):

```
                  k proportion           95%-CI  tau^2    tau     Q   I^2
Gender = Female   2     0.3541 [0.1467; 0.6363] 0.6215 0.7883 16.95 88.5%
Gender = Male     1     0.3889 [0.2691; 0.5238]     --     --  0.00    --
```

Test for subgroup differences (random effects model):

```
                  Q d.f. p-value
Between groups   0.05    1  0.8200
```

##
PA: Prevalence in Lebanon
##

> Country level

Number of studies combined: k = 3

```
                   proportion           95%-CI
Fixed effect model       0.3177 [0.2718; 0.3674]
Random effects model     0.3706 [0.1513; 0.6605]
Prediction interval             [0.0000; 1.0000]

Quantifying heterogeneity:
 tau^2 = 1.0588; tau = 1.0290; I^2 = 95.4%; H = 4.66

Test of heterogeneity:
     Q d.f.  p-value             Test
 48.48    2 < 0.0001        Wald-type
 57.24    2 < 0.0001 Likelihood-Ratio


> Prevalence by gender

Number of studies combined: k = 4

Results for subgroups (random effects model):
                  k proportion           95%-CI  tau^2    tau     Q   I^2
Gender = Male     2     0.2236 [0.1385; 0.3403] 0.1112 0.3334  5.23 62.3%
Gender = Female   2     0.2492 [0.0755; 0.5744] 0.9044 0.9510 15.93 88.1%

Test for subgroup differences (random effects model):
                    Q d.f. p-value
Between groups   0.03    1  0.8545
```

References:

1 Abbas A, Ali A, Bahgat SM, Shouman W. Prevalence, associated factors, and consequences of burnout among ICU healthcare workers: An Egyptian experience. The Egyptian Journal of Chest Diseases and Tuberculosis. 2019;68:514.

2 AbdAllah A, El-Hawy L. BURNOUT AND HEALTH RELATED QUALITY OF LIFE AMONG RESIDENT PHYSICIANS IN ZAGAZIG UNIVERSITY HOSPITALS. Egyptian Journal of Occupational Medicine. 2019;43:189-204.

3 Abdo S, El-Sallamy R, El-Sherbiny A, Kabbash I. Burnout among physicians and nursing staff working in the emergency hospital of Tanta University, Egypt. East Mediterr Health J. 2016;21:906-15.

4 Abdulaziz S, Baharoon S, Al Sayyari A. Medical residents’ burnout and its impact on quality of care. The clinical teacher. 2009;6:218-24.

5 Abdulrahman M, Nair SC, rasekhar, Farooq MM, Al Kharmiri A, Al Marzooqi F, et al. Burnout and depression among medical residents in the United Arab Emirates: A Multicenter study. Journal of family medicine and primary care. 2018;7:435-41.

6 Abdulrazak Abyad BA, Abbas Ali Masour. Frequency of Job-Related Burn-out in Family Physicians working in General / Family Practice in the Middle East. Middle East Journal of Family Medicine. 2016;14.

7 Agha A, Mordy A, Anwar E, Saleh N, Rashid I, Saeed M. Burnout among middle-grade doctors of tertiary care hospital in Saudi Arabia. Work (Reading, Mass). 2015;51:839-47.

8 Ahmadpanah M, Torabian S, Dastore K, Jahangard L, Haghighi M. Association of occupational burnout and type of personality in Iranian general practitioners. Work (Reading, Mass). 2015;51:315-9.

9 Al Dabbagh AM, Hayyawi AH, Kochi MS. Burnout Syndrome among Physicians Working in Primary Health Care Centers in Baghdad, Al-Rusafa Directorate, Iraq. SCOPUS IJPHRD CITATION SCORE. 2019;10:484.

10 Al Subhi A, Al Lawati H, Shafiq M, Al Kindi S, Al Subhi M, Al Jahwari A. Prevalence of burnout of residents in oman medical specialty board: A cross-sectional study in Oman. Journal of Musculoskeletal Surgery and Research. 2020;4:136-40.

11 Al-Dubai SAR, Rampal KG. Prevalence and associated factors of burnout among doctors in Yemen. Journal of occupational health. 2010;52:58-65.

12 Al-Haddad A, Al-Omar F, Al-Khaleel A, Al-Khalaf A. Prevalence of burnout syndrome and its related risk factors among physicians working in primary health care centers of the Ministry of Health, Al Ahsa region, Saudi Arabia, 2018-2019. Journal of family medicine and primary care. 2020;9:571.

13 Al-Hashemi T, Al-Huseini S, Al-Alawi M, Al-Balushi N, Al-Senawi H, Al-Balushi M, et al. Burnout Syndrome Among Primary Care Physicians in Oman. Oman medical journal. 2019;34:205-11.

14 Al-Sareai NS, Al-Khaldi YM, Mostafa OA, Abdel-Fattah MM. Magnitude and risk factors for burnout among primary health care physicians in Asir Province, Saudi Arabia. Eastern Mediterranean health journal = La revue de sante de la Mediterranee orientale = al-Majallah al-sihhiyah li-sharq al-mutawassit. 2013;19:426-34.

15 Al-Youbi RA, Jan MM. Burnout syndrome in pediatric practice. Oman medical journal. 2013;28:252-4.

16 Aldrees TM, Aleissa S, Zamakhshary M, Badri M, Sadat-Ali M. Physician well-being: prevalence of burnout and associated risk factors in a tertiary hospital, Riyadh, Saudi Arabia. Annals of Saudi medicine. 2013;33:451-6.
17 Aldrees T, Badri M, Islam T, Alqahtani K. Burnout Among Otolaryngology Residents in Saudi Arabia: A Multicenter Study. Journal of surgical education. 2015;72:844-8.
18 Aldrees T, Hassouneh B, Alabdulkarim A, Asad L, Alqaryan S, Aljohani E, et al. Burnout among plastic surgery residents. National survey in Saudi Arabia. Saudi medical journal. 2017;38:832-6.
19 Alhaffar BA, Abbas G, Alhaffar AA. The prevalence of burnout syndrome among resident physicians in Syria. J Occup Med Toxicol. 2019;14:31-.
20 Alotaibi AK, Alsalim A, Alruwaili F, Almubarak A, Alhamzah A, eri, et al. Burnout during ophthalmology residency training: A national survey in Saudi Arabia. Saudi journal of ophthalmology : official journal of the Saudi Ophthalmological Society. 2019;33:130-4.
21 Alqahtani AM, Awadalla NJ, Alsaleem SA, Alsamghan AS, Alsaleem MA. Burnout Syndrome among Emergency Physicians and Nurses in Abha and Khamis Mushait Cities, Aseer Region, Southwestern Saudi Arabia. TheScientificWorldJournal. 2019;2019:4515972.
22 Alsaawi A, Alrajhi K, Albaiz S, Al-Sultan M, Alsalamah M, Qureshi S, et al. Risk of burnout among emergency physicians at a tertiary care centre in Saudi Arabia. Journal of Hospital Administration. 2014;3:20.
23 Alsaawi A, Alrajhi K, AlRasheed N, AlSabhan A, AlTamimi D, Alsalamah M. Burnout among Emergency Physicians in Saudi Arabia: A Cross-sectional Study. Journal of Health Informatics in Developing Countries. 2019;13.
24 Alsheikh KA, Alhabradi FA, Almalik FF, Alsalim AA, Ahmed FE, Alhandi AA. Burnout syndrome among orthopedic surgery residents in Saudi Arabia: A multicenter study. Journal of Musculoskeletal Surgery and Research. 2019;3:184.
25 Alyamani A, Alyamani L, Altheneyan F, Aldhali S, Albaker K, Alshaalan A, et al. Prevalence of burnout among residents at king Abdulaziz Medical City in Riyadh, Saudi Arabia. International Journal of Medical Research & Health Sciences. 2018;7:37-40.
26 Arv, i Z, Emami A, Zarghi N, Alavinia SM, Shirazi M, et al. Linking medical faculty stress/burnout to willingness to implement medical school curriculum change: a preliminary investigation. Journal of evaluation in clinical practice. 2016;22:86-92.
27 Ashkar K, Romani M, Musharrafieh U, Chaaya M. Prevalence of burnout syndrome among medical residents: experience of a developing country. Postgraduate medical journal. 2010;86:266-71.
28 Bany Hamdan A, Alshammary S, Javison S, Tamani J, AlHarbi M. Burnout Among Healthcare Providers in a Comprehensive Cancer Center in Saudi Arabia. Cureus. 2019;11:e3987.
29 Belayachi J, Rkain I, Rkain H, Madani N, Amlaiky F, Zekraoui A, et al. Burnout Syndrome in Moroccan Training Resident: Impact on Quality of Life. Iranian journal of public health. 2016;45:260-2.
30 Ben Zid A, Homri W, Ben Romdhane I, Bram N, Labbane R. Burnout chez les résidents en médecine tunisiens : À propos de 149 cas = Burnout in Tunisian medical residents: About 149 cases. L'Encéphale: Revue de psychiatrie clinique biologique et thérapeutique. 2018;44:337-42.

31 Benbrahim Z, Ibtihal A, Boujarnija R, Atassi M, Amaadour L, Oualla K, et al. Burnout among Moroccan Oncologists: A National Survey. Global Journal on Quality and Safety in Healthcare. 2019;2:85-8.
32 Bin Dahmash A, Alhadlaq AS, Alhujayri AK, Alkholaiwi F, Alosaimi NA. Emotional Intelligence and Burnout in Plastic Surgery Residents: Is There a Relationship? Plastic and reconstructive surgery Global open. 2019;7:e2057.
33 Chaudhry MA, Khokhar MM, Waseem M, Alvi ZZ, ul Haq AI. Prevalence and associated factors of burnout among military doctors in Pakistan. Pakistan Armed Forces Medical Journal. 2015;65:669-73.
34 El Kettani A, Serhier Z, Othmani MB, Agoub M, Battas O. [Evaluation of burnout syndrome among doctors in training at the Ibn Rochd University Hospital, Casablanca]. The Pan African medical journal. 2017;27:243.
35 Khan FA, Shamim MH, Ali L, Taqi A. Evaluation of Job Stress and Burnout Among Anesthesiologists Working in Academic Institutions in 2 Major Cities in Pakistan. Anesth Analg. 2019;128:789-95.
36 Farahat TM, Hegazy NN, Mohamed DH. Burnout among physicians in Qalubia primary health care facilities, Egypt. Journal of Family Medicine and Health Care. 2016;2:1-5.
37 Farahat TM, Hegazy NN, Mohamed DH. Burnout and quality of life among physicians in primary healthcare facilities in Egypt: a cross-sectional study. Menoufia Medical Journal. 2017;30:789.
38 Al-Shoraian GMJ, Hussain N, Alajmi MF, Kamel MI, El-Shazly MK. Burnout among family and general practitioners. Alexandria Journal of Medicine. 2011;47:359-64.
39 Hamdan M, Hamra AaA. Burnout among workers in emergency Departments in Palestinian hospitals: prevalence and associated factors. BMC health services research. 2017;17:407.
40 Hameed TK, Masuadi E, Al Asmary NA, Al-Anzi FG, Al Dubayee MS. A study of resident duty hours and burnout in a sample of Saudi residents. BMC medical education. 2018;18:180.
41 Hasan HI, Nooh Y, Alsayyad AS. Prevalence and factors affecting burnout among secondary care doctors in Bahrain-a cross sectional study. International Journal of Medical Research & Health Sciences. 2015;4:401-6.
42 Hussein H, Al Faisal W, Wasfy A, Monsef N, AbdulRahim W, El Sawaf E, et al. Burnout among primary health care physicians in Dubai health authority Dubai-UAE. Age. 2016;30:4.9.
43 Kacem I, Kalboussi H, Ayoub N, Brahem A, Maoua M, Boughattas W, et al. burn-out chez les jeunes médecins: Étude réalisée dans la région de sousse. [Burn-out among young doctors: Study conducted in the Sousse region.]. Annales Médico-Psychologiques. 2017;175:332-8.
44 Jadoo SAA, Dastan I, Al-Samarrai MAM, Yaseen SM, Torun P. Predictors of emotional exhaustion among physicians from Iraq-a descriptive cross-sectional multicentre study. Journal of ideas in Health. 2018;1:42-9.
45 Jalili M, Sadeghipour Roodsari G, Bassir Nia A. Burnout and Associated Factors among Iranian Emergency Medicine Practitioners. Iranian journal of public health. 2013;42:1034-42.
46 Jamjoom RS, Park YS. Assessment of pediatric residents burnout in a tertiary academic centre. Saudi medical journal. 2018;39:296-300.

47 Musharrafieh U. Prevalence of Burnout in Lebanese Practicing Physicians: Experience of a Developing Country. 2019;66:260-6.

48 Kalani S, Azad Fallah P, Oreyzi HR, Azizkhani R, Adibi P. Prevalence of burnout syndrome among the residents in Isfahan University of Medical Sciences, Isfahan, Iran. JOURNAL OF ISFAHAN MEDICAL SCHOOL (IUMS). 2017;35:993-9.

49 Khan K, Ejaz, S., Naeem, N.- i-K., & Khan, A. Burnout Syndrome and its Risk Factors among Gynecology Consultants. Journal of Aziz Fatimah Medical & Dental College. 2020;2:17-21.

50 Kotb AA, Mohamed KA-E, Kamel MH, Ismail MAR, Abdulmajeed AA. Comparison of burnout pattern between hospital physicians and family physicians working in Suez Canal University Hospitals. Arab Journal of Psychiatry. 2014;25:167-79.

51 Liaqat N, Dar SH, Waheed K. Burn out among the paediatric surgeons of Pakistan. Journal of Pakistan Medical Association. 2019;69:1730-3.

52 Alorabi M, Ghali R, Bolus D. Cross-sectional study of burnout among a group of Egyptian oncologists at Ain Shams University2015.

53 Oumaya M, Ghanmi L, Baccar Y, Khédira B, Salah K, Bouzid R. P03-259 Burnout or professional exhaustion syndrome among primary care Tunisian doctors. European Psychiatry - EUR PSYCHIAT. 2009;24.

54 Taei M, Safi zadeh H, Divsalar K. Frequency of Burnout among General Practitioners of Kerman City, 2008. Journal of Kerman University of Medical Sciences. 2010;17:268-76.

55 Almhana M, Abutiheen A, Al-Haidary A. Prevalence of Burnout among physicians in Kerbala, Iraq. Indian Journal of Public Health Research and Development. 2019;10:1001.

56 Malik AA, Bhatti S, Shafiq A, Khan RS, Butt UI, Bilal SM, et al. Burnout among surgical residents in a lower-middle income country - Are we any different? Annals of medicine and surgery (2012). 2016;9:28-32.

57 Marzouki HZ, Alhodaly H, Binrahma D, Alzahrani A, Alsubhi Y, Aljiffry M, et al., editors. Burnout and job satisfaction among physicians in a Saudi tertiary care center: a cross-sectional study2019.

58 Massou S, Doghmi N, Belhaj A, Aboulaala K, Azendour H, Haimeur C, et al. Enquête sur le syndrome d'épuisement professionnel chez les personnels d'anesthésie réanimation de quatre hôpitaux universitaires marocains = Survey on burn out in healthcare providers working in four Moroccan academic intensive care units. Annales Médico-Psychologiques. 2013;171:538-42.

59 Mazhar SB, Gilani S, Ain QT, Khan S. High burn out among doctors working in a tertiary care hospital; a wakeup call. JPMA The Journal of the Pakistan Medical Association. 2019;69:349-54.

60 Mhamdi S, Nakhli MS, Kahloul M, Latrech N, Rejeb MB, Khadhraoui M, et al. [Burnout prevalence in Tunisian anesthesia and intensive care units]. The Pan African medical journal. 2018;31:111.

61 Moein N, Ahmadzadeh G, Safaeeyan A. Job Burnout Among Family Physicians in Rural Areas of Isfahan Province. Hospital Practices and Research. 2018;3:98-103.

62 Mohammed KA-M, Ali EG, Youssef IM, Fahmy MT, Haggag WE-l. Depression and burnout among residents. Arab Journal of Psychiatry. 2014;25:40-51.

63 Mohammed SB, Hassan BA, Younis MS. Job satisfaction and burnout among Iraqi physicians: Insight from university hospital surveys. Arab Journal of Psychiatry. 2018;29:48-56.
64 Mortada E, Seifi O, editors. Clinicians ' Burnout and Career Satisfaction Across the Major Specialties in Zagazig University Hospitals2012.
65 Ok AEL, Em M, Aa E, Ae S. Burnout Syndrome among h ealthcare providers in different hospitals in Minia city. Egyptian Journal of Occupational Medicine. 2018;42:21-31.
66 Omar AS, Shouman Y, Sudarsanan S. Burnout signals are alarming worldwide: the active role of leadership. Qatar Med J. 2019;2019:52.
67 Osman D, Abdlrheem S. Burnout and Job Satisfaction among Healthcare Providers in Aswan University Hospital, Upper Egypt. Journal of High Institute of Public Health. 2019;49:64-72.
68 Sadat-Ali M, Al-Habdan IM, Al-Dakheel DA, Shriyan D. Are orthopedic surgeons prone to burnout? Saudi medical journal. 2005;26:1180-2.
69 Salem M, Taher M, Alsaadi H, Alnema A, Al-Abdulla S. Prevalence and determinants of burnout among primary healthcare physicians in Qatar. World Family Medicine Journal: Incorporating the Middle East Journal of Family Medicine. 2018;99:1-7.
70 Salem R, Akel R, Fakhri G, Tfayli A. Burnout among Lebanese Oncologists: Prevalence and Risk Factors. Asian Pacific journal of cancer prevention : APJCP. 2018;19:2135-9.
71 Selaihem AA. Prevalence of burnout amongst physicians working in primary care in Riyadh military hospital, Saudi Arabia. Int J Med Sci Public Health. 2013;2:410-9.
72 Shams T, El-Masry R. Job Stress and Burnout among Academic Career Anaesthesiologists at an Egyptian University Hospital. Sultan Qaboos University medical journal. 2013;13:287-95.
73 Sheikhmoonesi F, Khani F, Khademloo M, Saravi SFB. Prevalence of burnout among resident physicians in Mazandaran University of Medical Sciences, Iran. Global Journal of Health Science. 2017;9:50.
74 Sherbiny EE, Khashaba E, Abdelhady A. BURNOUT AMONG INTENSIVE CARE WORKERS IN A TERTIARY CARE HOSPITAL IN SAUDI ARABIA. Egyptian Journal of Occupational Medicine. 2017;41:289-306.
75 Soltanifar A, Pishbin E, Attaran Mashhadi N, Najaf Najafi M, Siahtir M. Burnout among female emergency medicine physicians: A nationwide study. Emergency medicine Australasia : EMA. 2018;30:517-22.
76 Yousef I.M. HAO, Elsayed O.I. and Ali. E.G. Burnout Syndrome among Resident Physician in Suez Canal University Hospital. Current psychiatry. 2006;13:24- 42.
77 Zarei E, Ahmadi F, Sial MS, Hwang J, Thu PA, Usman SM. Prevalence of Burnout among Primary Health Care Staff and Its Predictors: A Study in Iran. International journal of environmental research and public health. 2019;16.
78 Zubairi AJ, Noordin S. Factors associated with burnout among residents in a developing country. Annals of medicine and surgery. 2016;6:60-3.
